# Chemically Mediated Artificial Electron Transport Chain

**DOI:** 10.1021/acscentsci.4c00165

**Published:** 2024-04-10

**Authors:** Yu-Dong Yang, Qian Zhang, Lhoussain Khrouz, Calvin V. Chau, Jian Yang, Yuying Wang, Christophe Bucher, Graeme Henkelman, Han-Yuan Gong, Jonathan L. Sessler

**Affiliations:** † Department of Chemistry, The University of Texas at Austin, 105 East 24th Street, Stop A5300, Austin, Texas 78712-1224, United States; ‡ ENSL, CNRS, Laboratoire de Chimie UMR 5182, Laboratoire de Chimie, Lyon 69364, France; § College of Chemistry, Beijing Normal University, No. 19, XinJieKouWai St, HaiDian District, Beijing 100875, P. R. China

## Abstract

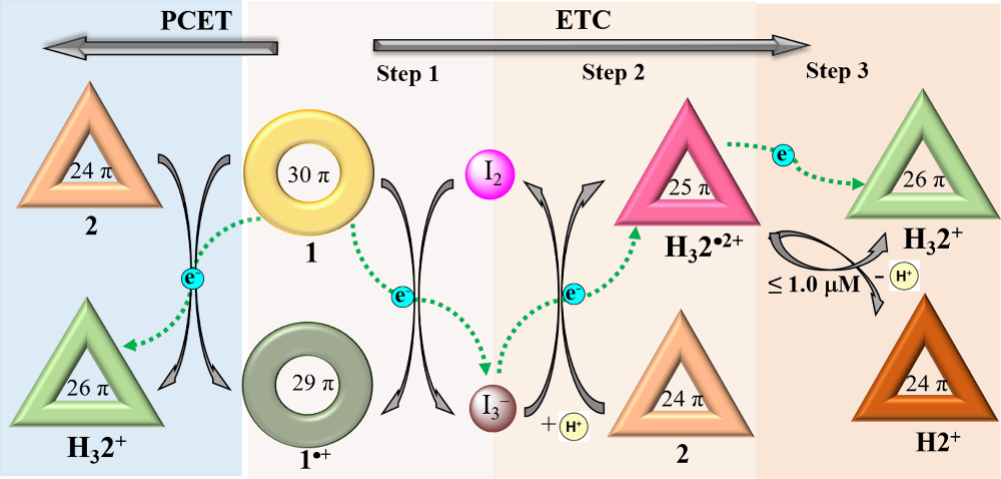

Electron transport chains (ETCs) are ubiquitous in nearly all living systems. Replicating the complexity and control inherent in these multicomponent systems using ensembles of small molecules opens up promising avenues for molecular therapeutics, catalyst design, and the development of innovative energy conversion and storage systems. Here, we present a noncovalent, multistep artificial electron transport chains comprising cyclo[8]pyrrole (**1**), a *meso*-aryl hexaphyrin(1.0.1.0.1.0) (naphthorosarin **2**), and the small molecules I_2_ and trifluoroacetic acid (TFA). Specifically, we show that 1) electron transfer occurs from **1** to give I_3_^–^ upon the addition of I_2_, 2) proton-coupled electron transfer (PCET) from **1** to give **H**_**3**_**2**^**•2+**^ and **H**_**3**_**2**^**+**^ upon the addition of TFA to a dichloromethane mixture of **1** and **2**, and 3) that further, stepwise treatment of **1** and **2** with I_2_ and TFA promotes electron transport from **1** to give first I_3_^–^ and then **H**_**3**_**2**^**•2+**^ and **H**_**3**_**2**^**+**^. The present findings are substantiated through UV-vis-NIR, ^1^H NMR, electron paramagnetic resonance (EPR) spectroscopic analyses, cyclic voltammetry studies, and DFT calculations. Single-crystal structure analyses were used to characterize compounds in varying redox states.

## Abstract 2

Presented is an example of a proton-coupled electron transfer (PCET) system comprising an artificial electron transport chain (ETC) that can be regulated by small molecules or concentration control.

## Introduction

Small molecules, such as nicotinamide adenine dinucleotide (NADH), flavin adenine dinucleotide (FADH_2_), ubiquinone, cytochrome c, chlorophyll, and pheophytin, play critical roles in biological electron transport chains (ETCs).^1−8^ Efforts to modulate these molecules and their function have led inter alia to the development of porphyrin analogues as photosensitizers for photodynamic therapy,^9−11^ strategies for enhancing reactive oxygen species (ROS) concentrations,^12−14^ the use of metformin, a Complex I inhibitor, to reduce tumorigenesis and treat other conditions,^15−18^ as well as the construction of artificial photosynthesis systems^19−21^ and dye-sensitized solar cells.^22−24^

Proton-coupled electron transfer (PCET) plays a critical role in a multitude of redox reactions, spanning from biological electron transport chains to numerous artificial catalyst and energy transfer systems.^25−29^ Numerous examples of electron transfer between two redox active entities are now known.^30−38^ Moreover, three-component electron transfers have been observed in some catalysis-focused reaction systems,^39−41^ particularly in organic photocatalysis.^42−44^ However, thermally driven electron transport chains mediated by small molecules and modulated through environmental changes (e.g., concentration) are far less explored, and the development of such systems constitutes an unmet challenge. This lack of development may reflect the difficulty associated with controlling complex, freestanding chemical systems and the corresponding stepwise orchestration of electron flow.

**Scheme 1:**
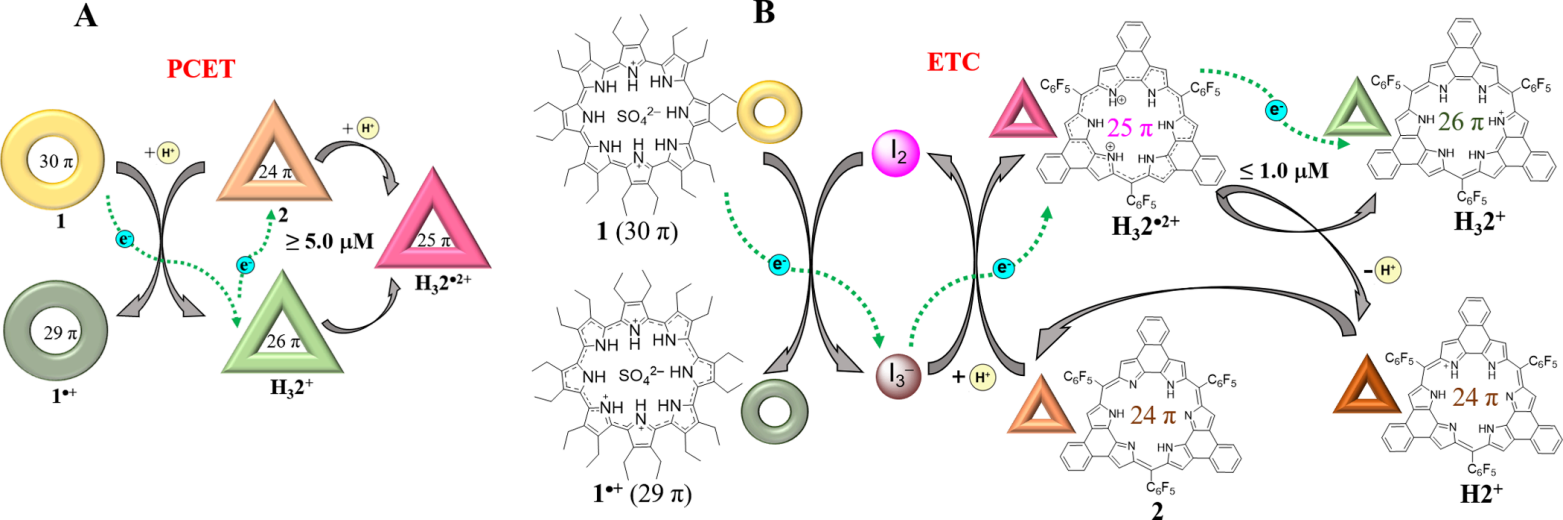
Schematic Representations of Non-Covalent Multi-Component Electron Transport Composed of 1, I_2_, and 2. (A) PCET from 1 to give H_3_2^+^ and H_3_2^•2+^. (B) ETC Involving 1, I_2_, and 2

While macrocycles have been widely employed in the development of electron transfer systems and related materials, only a limited number of examples involving macrocycle-based PCET are known.^45−47^ Notably, to our knowledge, there is no reported demonstration of PCET occurring between two macrocycles. Here, we report a novel PCET system between two macrocycles, cyclo[8]pyrrole **1** and *meso*-aryl hexaphyrin (1.0.1.0.1.0) (naphthorosarin **2**), upon the addition of TFA, as well as demonstrate a chemical mediated artificial electron transfer chain consisting of **1**, **2**, I_2_, and trifluoroacetic acid (TFA) (Scheme 1). We show that the reaction stoichiometry, along with the presence or absence of TFA, may be used to control the electron transfer events within this multicomponent system. Specifically, oxidation of the formal 30 π-electron (30 π) neutral aromatic form of **1** with I_2_ leads to the formation of the corresponding radical species **1**^**•+**^ (29 π), along with I_3_^–^. On the other hand, PCET between **1** and **2** is seen with the participation of TFA. Depending on the molar ratio of **1**, **2**, and the added TFA, different reduced forms of **2**, namely a radical **H**_**3**_**2**^**•2+**^ (25 π) and an aromatic cation **H**_**3**_**2**^**+**^ (26 π), are produced from the initial triprotonated antiaromatic species **H**_**3**_**2**^**3+**^ (24 π) (counteranion = CF_3_CO_2_^–^) (Scheme 1A). Adding I_2_ to a mixed solution of **1** and **2** in dichloromethane, leads to the selective formation of a one-electron oxidized form of **1** (**1**^**•+**^) and I_3_^–^ without affecting **2**. Upon addition of TFA to the solution, I_3_^–^ acts as an electron carrier and produces **H**_**3**_**2**^**•2+**^ while undergoing concomitant oxidation back to I_2_. The radical dication **H**_**3**_**2**^**•2+**^ disproportionates to **H2**^**+**^ (24 π) and **H**_**3**_**2**^**+**^ (26 π) at low concentrations (≤1.0 μM), a conversion that involves further electron transfer steps (Scheme 1B). The ability to control PCET between two macrocycles and associated thermally driven ETC processes through multiple redox steps and two different electron carriers (I_3_^–^ and **H**_**3**_**2**^**•2+**^), as demonstrated here, is expected to lead to an increased understanding of complex biological redox-based signaling, as well as small molecule therapeutic design and advances in energy conversion and storage.

## Results and Discussion

### Electron Transfer from 1 to I_2_

**Figure 1:**
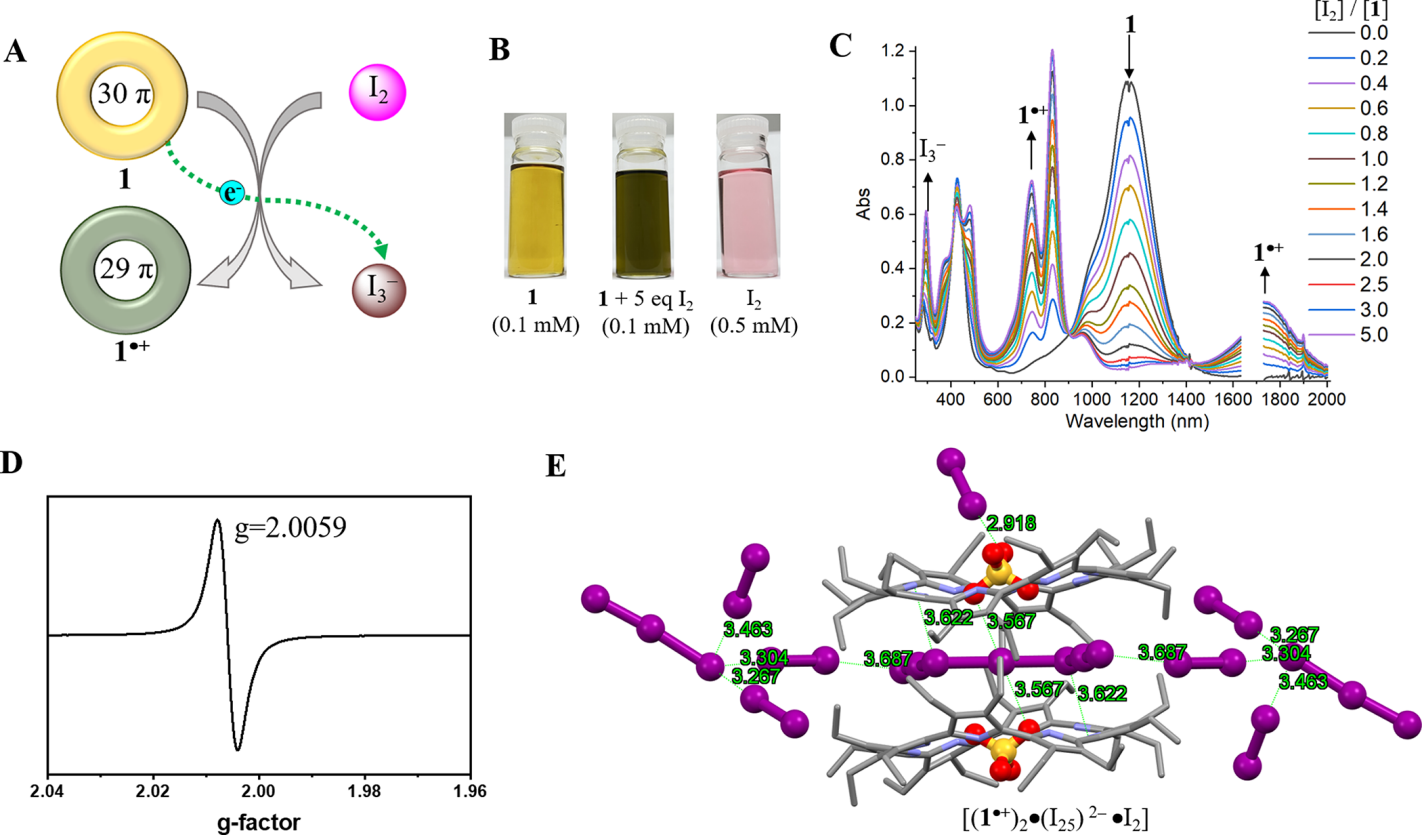
Solution and solid-state studies of the electron transfer from **1** to I_**2**_. (**A**) Schematic representation of the proposed electron transfer from **1** to I_2_. (**B**) Photographs of dichloromethane solutions of **1** (0.1 mM), a mixture of **1** (0.1 mM) and 5.0 eq of I_2_, and I_2_ (0.5 mM) alone. (**C**) UV-vis-NIR spectral changes seen when a solution of **1** (10 μM) in dichloromethane is titrated with I_2_. (**D**) EPR spectrum of **1** (0.1 mM) recorded in the presence of 5.0 eq of I_2_ in dichloromethane. (**E**) Side view of the iodic complex of **1**^**•+**^ seen in the solid-state structure of single crystals of [(**1**^**•+**^)_2_•(I_25_) ^2–^•I_2_]. Hydrogen atoms have been omitted for the sake of clarity.

Cyclo[8]pyrrole **1** is an expanded porphyrin that is relatively easy to oxidize.^48^ Cyclic voltammetry (CV) studies revealed a very low value for the first one-electron oxidation of **1**^**•+**^/**1** process (*E*_1/2_ = −0.12 V versus Fc^+^/Fc) along with good reversibility. This led us to explore whether I_2_, which has a more positive reduction potential I_2_/I_3_^–^ (*E*_pc_ = +0.05 V versus Fc^+^/Fc) determined under the same conditions, could be used to oxidize **1**. (Note: See Table S5 for a listing of redox potentials relevant to the present study). Adding 5 molar equiv (eq) of I_2_ into a solution of **1** (0.1 mM in dichloromethane) caused the orange color of the initial solution to change to brown-green immediately (Figure 1B). Further UV-vis-NIR spectral titrations revealed a decrease in the characteristic absorption maximum of **1** (λ_max_ = 1150 nm), as well as the simultaneous appearance of shoulders at 744 and 832 nm and a broad peak at 1750 nm. These latter features were readily assigned to **1**^**•+**^ based on independent preparations (see Figures S5–8, S39). A peak at λ_max_ = 296 nm ascribed to I_3_^–^ was also seen after addition (Figure 1C). The equilibrium constants corresponding to the reaction between **1** and I_2_ were calculated to be *K*_*a1*_ = (2.5 ± 0.2) × 10^5^ M^–1^, *K*_*a2*_ = (1.0 ± 0.1) × 10^5^ M^–1^, and *K*_*a3*_ = (3.5 ± 0.3) × 10^6^ M^–1^ for a 2:3 binding interaction for complex **1**^**•+**^•I_3_^–^ as inferred from a UV-vis-NIR spectroscopic Job plots and associated titration studies (Figure S3–4). Subsequent ^1^H NMR spectral studies in dichloromethane-*d*_*2*_ revealed the disappearance of the ethyl group signals for **1** upon the addition of 5 molar eq of I_2_ (Figure S9). An electron paramagnetic resonance (EPR) spectral analysis of a 1:5 mixture of **1** and I_2_ in dichloromethane confirmed the presence of a strong signal at g = 2.0059 as expected for the formation of an organic radical species (Figure 1D). Collectively, these findings were taken as evidence that electron transfer from **1** to I_2_ occurs to produce the one-electron oxidized radical form **1**^**•+**^.

An X-ray diffraction analysis of the iodic complex of **1**^**•+**^ provided additional evidence for the proposed electron transfer between **1** and I_2_. Single crystals of [(**1**^**•+**^)_2_•(I_25_)^2–^•I_2_] containing a polymeric iodine cluster were obtained by exposing a 1.0 mM dichloromethane solution of **1** containing 10 molar eq of I_2_ to *n*-hexane vapor for 2 days at 298 K. The resulting structure is shown in Figure 1E. Conversion of **1** to radical **1**^**•+**^ led to a loss in bond length uniformity (Figure S30) as reflected in the difference between the shortest and longest interpyrrole C–C bond (Δd) within the macrocyclic core (Δd = 0.03 Å vs 0.10 Å for **1** and **1**^**•+**^, respectively). In addition, a greater deviation from the mean plane (defined by all core atoms) is seen in **1**^**•+**^ (0.51 Å) as compared to **1** (0.38 Å).^48^ The overall complex consists of two molecules of **1**^**•+**^ bound to an I_25_^2–^ cluster (a collection of 25 atoms that share two total negative charges) and one molecule of I_2_, a structure that is stabilized by presumed halogen-π and anion-π interactions. The closest iodine-to-pyrrole distances are on the order of 3.6–3.9 Å (Figure S31).

### PCET from 1 to 2

Naphthorosarin **2** exhibits distinctive redox reactivity upon protonation (cf. Table S5 in the Supporting Information). For instance, the (24 π) antiaromatic species (**2**) can be converted to the triply protonated state **H**_**3**_**2**^**3+**^ or, separately, reduced to give **H**_**3**_**2**^**•2+**^ (25 π) or **H**_**3**_**2**^**+**^ (26 π) upon treatment with certain acids F_3_CSO_3_H, HCl, or HI, respectively.^47^ In dichloromethane and in the presence of 20 molar eq of TFA, the two one-electron reduction potentials of **H**_**3**_**2**^**3+**^ and **H**_**3**_**2**^**•2+**^ at +0.41 and +0.02 (*E*_1/2_, versus Fc^+^/Fc), respectively, is more positive than the one-electron oxidation potential of **1**^**•+**^/**1** (*E*_1/2_ = −0.10 V, versus Fc^+^/Fc) (Table S5). We thus postulated that **1** would be able to reduce the triply protonated form of naphthorosarin **2** (**H**_**3**_**2**^**3+**^) to the corresponding 25 or 26 π form.

**Figure 2:**
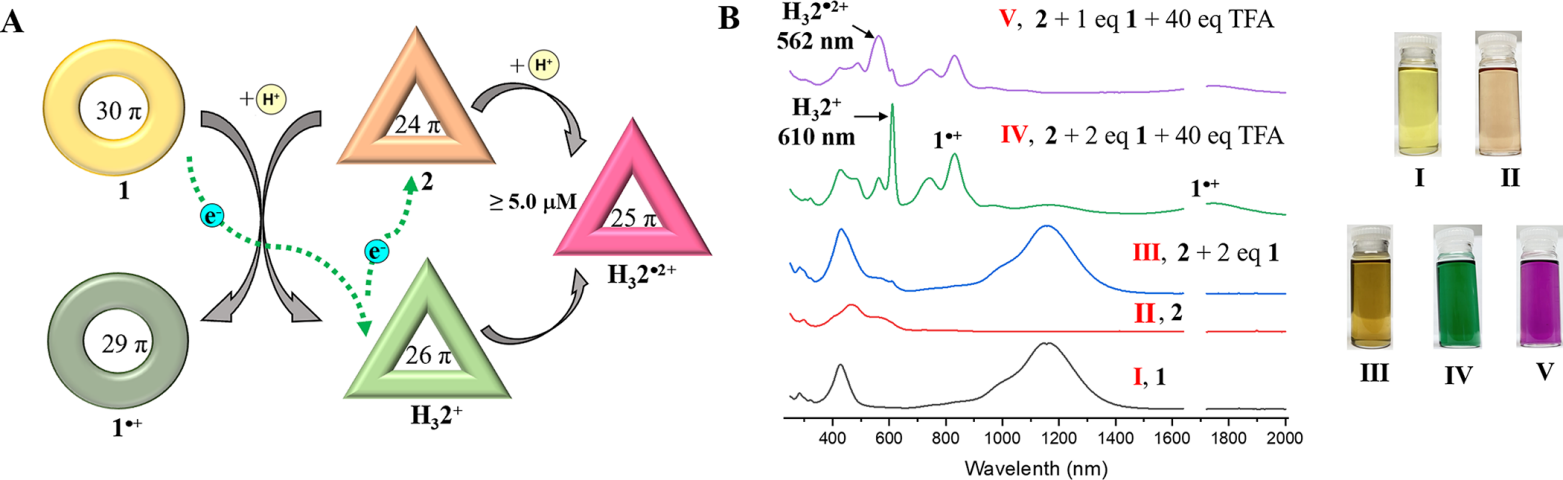
Solution studies of electron transfer from **1** to **2**. (**A**) Schematic representation of electron transfers from **1** to give **H**_**3**_**2**^**+**^ and **H**_**3**_**2**^**•2+**^. (**B**) UV–vis-NIR spectra and photographs of various samples: **I**) **1** (5.0 μM), **II**) **2** (5.0 μM), **III**) a mixture of **2** (5.0 μM) and 2.0 eq **1**, **IV**) a mixture of **2** (5.0 μM), 2.0 eq 1, and 40 eq of TFA, and **V**) a mixture of **2** (5.0 μM), 1.0 eq **1**, and 40 eq of TFA in dichloromethane.

To test this hypothesis, UV-vis-NIR spectral studies were carried out. Mixing **2** with one or two molar eq of **1** in dichloromethane did not result in obvious color or spectral changes. However, addition of 40 molar equiv of TFA to these solutions led to an immediate color change from the initial light brown to pink and green, respectively. In addition to features corresponding to **1**^**•+**^ resulting from oxidation of **1**, new absorption peaks at 562 and 620 nm were observed (Figure 2B). These signals correspond to the 25 π (**H**_**3**_**2**^**•2+**^) and 26 π (**H**_**3**_**2**^**+**^) species as reported previously.^47^ Adding ≥20, or even 40, molar equiv of TFA into solutions of **2** or **1** alone produced only minor changes in the absorption spectral features, a finding that leads us to suggest that the observed changes reflect PCET between **1**, **2**, and TFA rather than simple protonation effects (Figure S11–13).

Further UV-vis-NIR spectral titrations were performed by adding TFA portion-wise into a 1:1 solution of **1** and **2** (Figure S15). In this titration, **H**_**3**_**2**^**+**^ was observed as the primary product after the addition of 5.0 molar eq of TFA, with **H**_**3**_**2**^**•2+**^ being formed upon the addition of further TFA. This finding is consistent with **H**_**3**_**2**^**+**^ being oxidized back to **H**_**3**_**2**^**•2+**^ by **H**_**3**_**2**^**3+**^ in the presence of an excess of TFA and a deficit of **1**. In an effort to simplify the underlying interactions, a second set of titrations was performed (Figure S16a-c); this was done by adding TFA into a 2:1 solution of **1** and **2**. Under these conditions, a redox reaction between **H**_**3**_**2**^**+**^ and **2** is precluded due to the presence of **1** in excess. Indeed, upon completion of the titration, **H**_**3**_**2**^**+**^ was observed as the primary product. Collectively, these observations are taken as evidence of equilibrium-driven interconversions between the three limiting redox states of **2**, namely the 24 π, 25 π, and 26 π species, and the oxidized and reduced forms of **1** as shown in equations (Equats.) 1 and 2. The corresponding equilibrium constants were calculated as *K*_*1*_ = (1.5 ± 0.2) × 10^18^ M^–2^, and *K*_*2*_ = (7.6 ± 0.5) × 10^5^ M, respectively (Figure S16 d-g). This allowed the approximate populations of **2**, **H**_**3**_**2**^**•2+**^, and **H**_**3**_**2**^**+**^ to be determined; gratifyingly, the resulting values were found concordant with those calculated based on the change in the absorption intensities of the respective species (Figure S16).


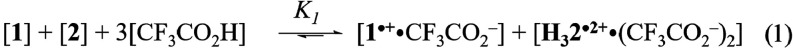
1


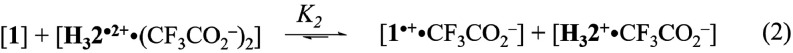
2

**Figure 3:**
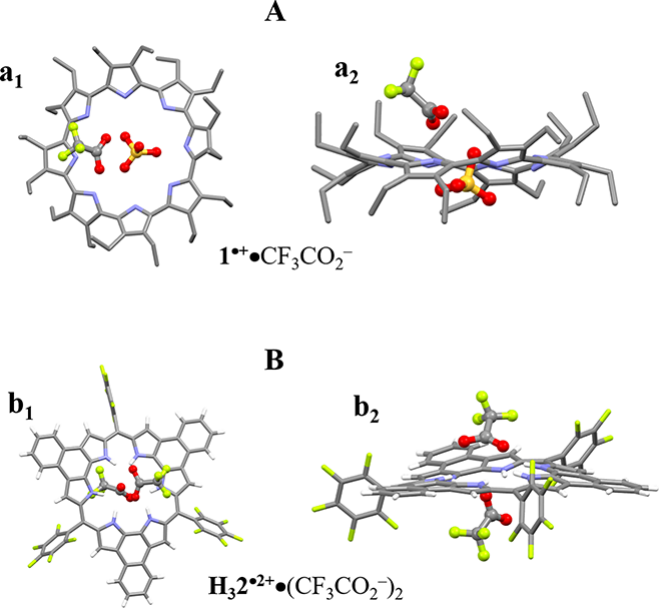
Solid-state studies of electron transfer from 1 to 2. (**A**) and (**B**) Top (**a**_**1**_, **b**_**1**_) and side views (**a**_**2**_, **b**_**2**_) of the complexes **1**^**•+**^•CF_3_CO_2_^–^ and **H**_**3**_**2**^**•2+**^•(CF_3_CO_2_^–^)_2_ seen in single crystals of [**1**^**•+**^•CF_3_CO_2_^–^] and [**H**_**3**_**2**^**•2+**^•(CF_3_CO_2_^–^)_2_•CHCl_3_], respectively. Solvents molecules and some hydrogen atoms have been omitted for clarity.

^1^H NMR spectral studies of **2** (0.5 mM in dichloromethane-*d*_2_) containing 1.0 or 2.0 molar eq of **1** revealed no discernible signals when recorded in the presence 20 molar eq of **TFA** (Figures S17 and 18). An EPR spectral analysis of this solution revealed a strong signal at g = 2.000 ascribed to the radical species **1**^**•+**^ and **H**_**3**_**2**^**•2+**^ (Figure S19). Exposure of the mixed solution consisting of **2** (0.5 mM in dichloromethane), **1** (1.0 or 2.0 equiv), and TFA (20 equiv) to ethyl acetate vapor for 2 days yielded single crystals of [**1**^**•+**^•CF_3_CO_2_^–^] in the case of both solutions. Structural analysis of the resulting complex [**1**^**•+**^•CF_3_CO_2_^–^], revealed that the conformation of **1**^**•+**^ was similar to that of [(**1**^**•+**^)_2_•(I_25_)^2–^•I_2_]. One molecule of CF_3_CO_2_^–^ was found bound outside of the cavity, through apparent N–H–O hydrogen bonds and CF−π interactions (Figure 3A). Exposure of the above solutions to *n*-hexane vapor for 2 days led to the formation of small dark crystals, which following recrystallization from CHCl_3_, gave single crystals of [**H**_**3**_**2**^**•2+**^•(CF_3_CO_2_^–^)_2_•CHCl_3_]. The deviation from the mean plane (defined by all core atoms) is 0.27 Å in **H**_**3**_**2**^**•2+**^, which is comparable to what is seen in **2** (0.23 Å) (Figure 3B).

Considered in concert, these results collectively provide support for the conclusion that electron transfer from **1** to **2** is coordinated with the protonation of **2** by TFA. Both electron transfer and protonation occur when all three compounds are involved. This cooperative-like function differs from classical PCET where both the electron and proton are transferred to or from the same compound.

### Electron Transport Chain Composed of 1, I_2_, and 2

**Figure 4:**
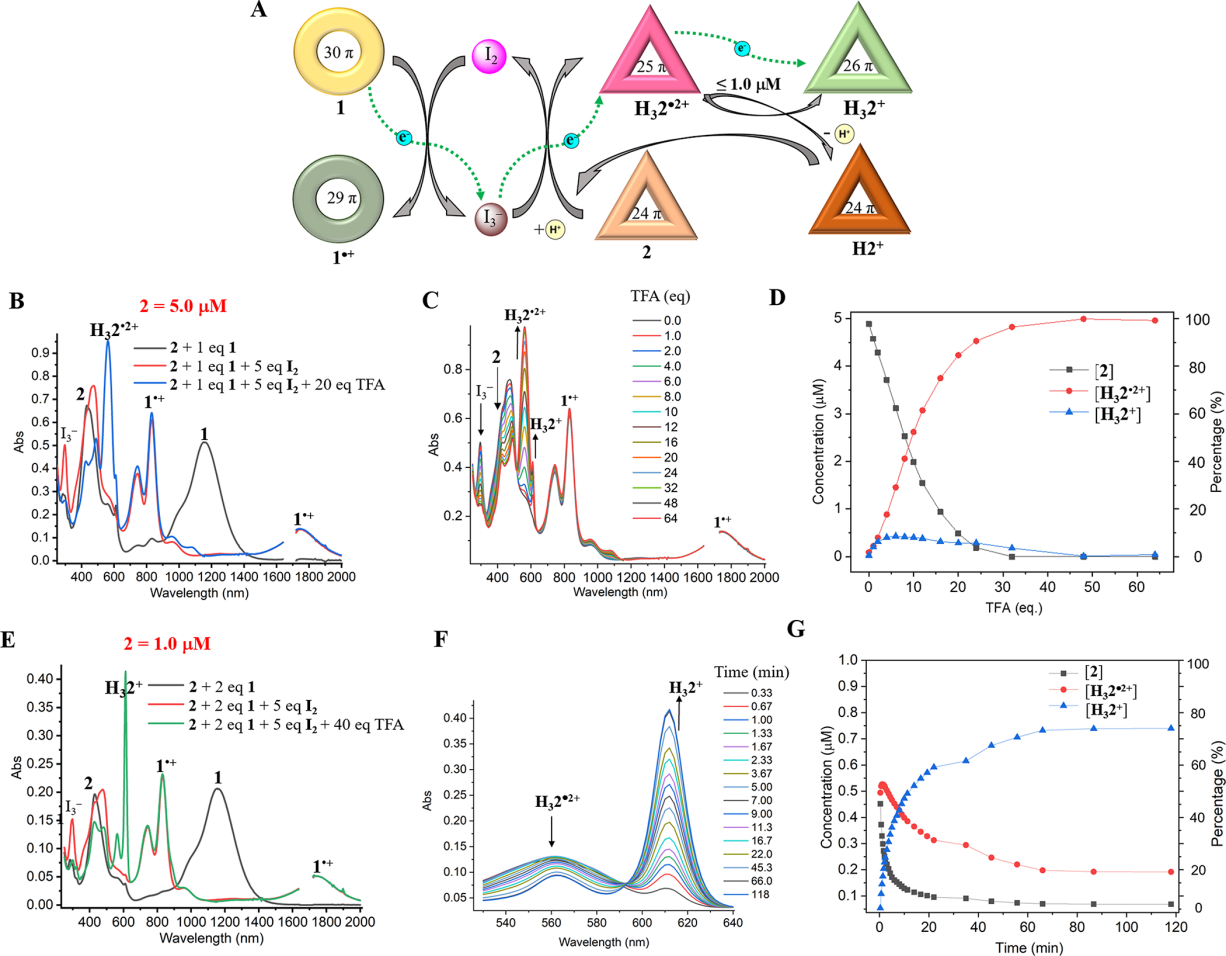
Solution studies of electron transfer reactions involving **1**, I_2_, and 2. (**A**) Schematic representation of the proposed electron transport chain. (**B**) UV-vis-NIR spectra for mixed solutions of **2** (5.0 μM) and 1.0 eq 1 recorded upon the stepwise addition of 5.0 eq of I_2_ and 20 eq of TFA in dichloromethane. (**C**) UV-vis-NIR spectral titrations of TFA into a mixture of **2** (5.0 μM), 1.0 eq **1**, and 5.0 eq I_2_. (**D**) Plot of the concentration and percentage of **2**, **H**_**3**_**2**^**•2+**^, and **H**_**3**_**2**^**+**^ vs TFA based on the data presented in C. (**E**) UV-vis-NIR spectra for mixed solutions of **2** (1.0 μM) and 2.0 eq **1** recorded upon the stepwise addition of 10 eq of I_2_ and 40 eq of TFA. (**F**) Time-dependent UV-vis-NIR spectra of a mixed solution of **2** (1.0 μM), 2.0 molar eq of **1**, 10 molar eq of I_2_, and 40 molar eq of TFA. (**G**) Plot of concentration and percentage of **2**, **H**_**3**_**2**^**•2+**^, and **H**_**3**_**2**^**+**^ vs time based on the data in part F.

In an acidic environment (20 mM TFA in dichloromethane), the reduction potential of I_2_/I_3_^–^ (*E*_pc_ = +0.05 V versus Fc^+^/Fc) obtained from cyclic voltammetry studies, is more positive than the oxidation potential of **1**^**•+**^/**1** (*E*_1/2_ = −0.10 V versus Fc^+^/Fc). Likewise, the oxidation potential of I_2_/I_3_^–^ (*E*_pa_ = +0.25 V versus Fc^+^/Fc) is more negative than the reduction potential of **H**_**3**_**2**^**3+**^/**H**_**3**_**2**^**•2+**^ (*E*_1/2_ = +0.41 V versus Fc^+^/Fc) but more positive than the reduction potential of **H**_**3**_**2**^**•2+**^/**H**_**3**_**2**^**+**^ (*E*_1/2_ = +0.02 V versus Fc^+^/Fc) (Table S5 in the Supporting Information). These values, in conjunction with the above predicative studies, led us to test whether **1**, **I**_**2**_, and **2** in concert would serve as a noncovalent electron transport chain subject to chemical modulation by TFA. With this consideration in mind, a mixed solution of **1** (5.0 μM), **2** (1.0 equiv), and I_2_ (5.0 equiv) in dichloromethane was prepared and analyzed using UV–vis-NIR spectroscopy. The results showed that **1** was oxidized to **1**^**•+**^ with the characteristic absorption peak at 296 nm for I_3_^–^ appearing concurrently. However, under these conditions no changes in the absorption features for naphthorosarin **2** were seen (Figure 4B). These observations are interpreted in terms of the electron transfer between **1** and I_2_.

Upon adding TFA to the above mixture, a discernible color change (from light brown to pink) and a significant increase in the intensity of peak at 562 nm characteristic of the reduced 25 π form **H**_**3**_**2**^**•2+**^ was seen. A decrease in the intensity of the 296 nm band corresponding to I_3_^–^ was also observed, but no changes in the absorption features for **1**^**•+**^ were noted (Figure 4B). The spectra and concentration changes for **2**, **H**_**3**_**2**^**•2+**^, and **H**_**3**_**2**^**+**^ seen upon treatment with TFA are shown in Figure 4C, 4D. As **2** did not display any apparent absorption change in the presence of I_2_ (5.0 equiv), either in the presence or absence of TFA (Figure S21), we interpret these results in terms of conversion of **2** to **H**_**3**_**2**^**•2+**^ mediated by oxidation of I_3_^–^ by **H**_**3**_**2**^**3+**^. An increase in the concentration of **H**_**3**_**2**^**•2+**^ is also observed when TFA is added to a mixture of **2** (5.0 μM), I_2_ (3.5 equiv), and I_3_^–^ (1.0 eq, as its tetrabutylammonium (TBA^+^) salt) (Figure S22). We thus suggest that under these conditions the I_3_^–^ produced as a byproduct of the oxidation of **1** acts as an electron carrier and transports electrons to **2** upon protonation, as shown in Figure 4A.

With regard to the second step in the proposed electron transfer chain, we note that I_3_^–^ on its own is unable to reduce **H**_**3**_**2**^**•2+**^ to **H**_**3**_**2**^**+**^ as inferred from their respective redox potentials. However, as shown in Figures 4C and 4D, when subjected to titration with 1.0–4.0 molar eq of TFA, a small amount of the 26 π species **H**_**3**_**2**^**+**^, characterized by an absorption maximum at 612 nm, is also observed. The level of this species does not increase as the concentration of **H**_**3**_**2**^**•2+**^ is raised (>1.0 μM) (Figure 4D). We thus suggest that the observed **H**_**3**_**2**^**+**^ comes from the disproportionation of **H**_**3**_**2**^**•2+**^ at low concentrations. The disproportionation mentioned here necessarily involves a further electron transfer process. Concentration dependent UV-vis-NIR spectroscopic studies of crystalline **H**_**3**_**2**^**•2+**^•(CF_3_CO_2_^–^)_2_ dissolved in dichloromethane revealed that upon dilution, the percentage of **H**_**3**_**2**^**•2+**^ decreased, whereas that of **H**_**3**_**2**^**+**^ increased. Essentially all of the **H**_**3**_**2**^**•2+**^ disproportionates to produce a 1:1 mixture of **H**_**3**_**2**^**+**^ and the protonated 24 π species, **H2**^**+**^, when the concentration of all species is ≤1.0 μM, as deduced from UV-vis-NIR spectroscopic analyses (Figure S23) and DFT calculations (Tables S6, S7).

In a separate experiment, **2** (1.0 μM), **1** (2.0 equiv), and I_2_ (10 equiv) were mixed in dichloromethane. Under these conditions only **1**^**•+**^ and I_3_^–^ are generated; however, the subsequent addition of 40 molar eq of TFA produces the reduced 26 π species, **H**_**3**_**2**^**+**^, within 60 min as reflected in the production of its characteristic absorption feature at 612 nm (Figure 4E). Time-dependent UV-vis-NIR spectroscopic studies revealed that **2** was reduced to **H**_**3**_**2**^**•2+**^ first and then further to **H**_**3**_**2**^**+**^ via the disproportionation of **H**_**3**_**2**^**•2+**^ (Figure 4F, 4G). Increasing the concentration of **2** by 10- to 100-fold promotes aggregation and stabilization of **H**_**3**_**2**^**•2+**^. This prevents its disproportionation and is presumably promoted by the weak π–π intersubunit interactions seen in the solid-state structure of **H**_**3**_**2**^**•2+**^•(CF_3_CO_2_^–^)_2_, with the result that little if any **H**_**3**_**2**^**+**^ is produced (Figures S27 and S28). In contrast, under low concentration conditions (e.g., 1.0 μM), the electron carrier I_3_^–^ transports an electron to **H**_**3**_**2**^**•2+**^, which undergoes disproportionation to give **H2**^**+**^ and **H**_**3**_**2**^**+**^. DFT calculations (vide infra) support this conclusion (Tables S6, S7). [Note: I_3_^–^ is unable to reduce **H**_**3**_**2**^**•2+**^ to **H**_**3**_**2**^**+**^.] The net result is an electron transport chain with three steps involving multiple redox active species, as illustrated in Figure 4A.

**Figure 5:**
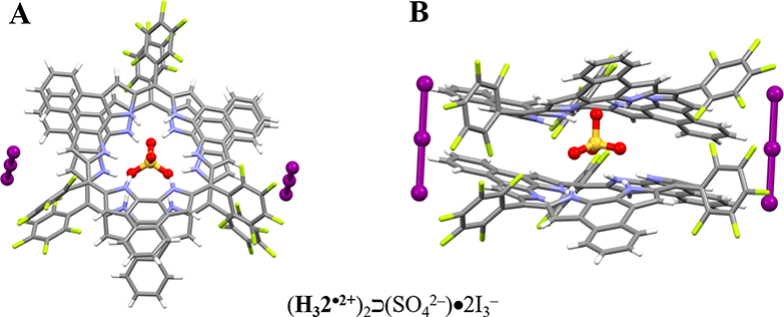
Single crystal structure of (H_3_2^•2+^)_2_⊃SO_4_^2–^)•2I_3_^–^. (A) Top and (B) side views of (H_3_2^•2+^)_2_⊃SO_4_^2–^)•2I_3_^–^ seen in single crystals of [(H_3_2^•2+^)_2_⊃(SO_4_^2–^)•2I_3_^–^•2.5I_2_•6.25H_2_O]. Some solvent molecules and hydrogen atoms have been omitted for the sake of clarity.

Exposing a dichloromethane solution of **1**, I_2_ (5.0 equiv), **2** (0.5 or 1.0 equiv), and 20 molar eq of TFA to ethyl acetate vapor for 2 days produced single crystals of [**1**^**•+**^•CF_3_CO_2_^–^] and [(**H**_**3**_**2**^**•2+**^)_2_**⊃**(SO_4_^2–^)•2I_3_^–^•2.5I_2_•6.25H_2_O] as a separable mixture. A core dimer (**H**_**3**_**2**^**•2+**^)_2_**⊃**(SO_4_^2–^) was found in the resulting structure [(**H**_**3**_**2**^**•2+**^)_2_**⊃**(SO_4_^2–^)•2I_3_^–^•2.5I_2_•6.25H_2_O] wherein the sulfate anion is sandwiched between the two **H**_**3**_**2**^**•2+**^ subunits and held in place via presumed N–H–O hydrogen bonds and possible π–π interactions. The two **H**_**3**_**2**^**•2+**^ subunits are identical and quasi-planar as reflected in a mean plane deviation of 0.58 Å (Figure 5).

### DFT Calculations

**Figure 6:**
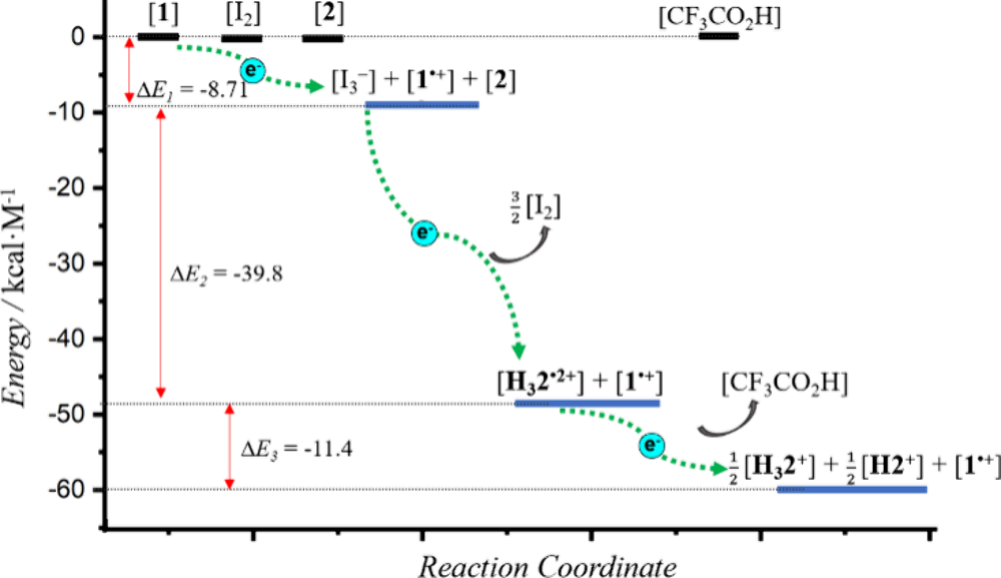
Optimized relative-energy profiles for complexes produced from **1**, I_2_, and **2** generated along the proposed electron transport chain. Their optimized geometries and relative energies were obtained by DFT calculations using the 6-311+G(d,p)-D3 and def2-TZVPD basis sets (Note: The CF_3_CO_2_^–^ counteranions have been omitted for clarity).

Further support for the suggestion that **1**, I_2_, and **2** interact to form an electron transport chain came from theoretical calculations. Based on the single crystal structures reported in this study and previous reports,^47,48^ the lowest energies in dichloromethane for **1**, I_2_, **2**_,_ TFA, and their ion species were calculated via DFT methods using the Gaussian 09 program.^49^ The resulting reaction energies (Δ*E*) are given in Figure 6 and Table S6, S7. It was found that the reaction energies of the relevant species decreased along the proposed electron transfer sequence, leading us to infer that the experimentally observed electron transport events are driven by thermodynamics.

## Conclusions

Presented here is an unusual example of a PCET system and an artificial electron transport chain that consists of multiple redox active chemical components, including cyclo[8]pyrrole (**1**), naphthorosarin (**2**), and I_2_. The electron transfer events within these ensembles can be regulated by treatment with small molecules, such as TFA, or via concentration control. Depending on the molar ratio of **1**, **2**, and added TFA, different reduced forms of **2**, namely **H**_**3**_**2**^**•2+**^ and **H**_**3**_**2**^**+**^ are produced via the PCET process. Transport of electrons from **1** to give I_3_^–^ could be induced by the treatment with I_2_. At high concentrations (≥5.0 μM) the I_3_^–^ produced in this way transfers electrons to the protonated form of **2** to produce **H**_**3**_**2**^**•2+**^. At low concentrations (e.g., 1.0 μM), **H**_**3**_**2**^**•2+**^ undergoes further disproportionation-mediated electron transport to give **H2**^**+**^ and **H**_**3**_**2**^**+**^. The level of specificity we show here and the demonstration of a *bona fide* electron transport chain are not readily replicated when three redox active components are mixed. Rather, direct electron transfer from the best electron donor to the best electron acceptor occurs directly in marked contrast to what is observed in, e.g., the respiratory electron transport chain. Nevertheless, in preliminary work, we have found that I_2_ can be replaced by (*tris*-(4-bromophenyl)ammoniumyl hexachloroantimonate, [(*p*-BrC_6_H_4_)_3_N^**•**^]^+^•[SbCl_6_]^−^), which acts as an effective mediator to promote electron transfer from cyclo[8]pyrrole (**1**) to naphthorosarin (**2**) (Figure S40). This finding lends support to the suggestion that the present findings may be readily generalizable, provided the systems in question are the product of appropriate design. More broadly, we believe that our results can serve as a useful framework for the design and development of novel artificial electron transport systems with potential applications in various fields such as energy storage and conversion, catalysis, and electronics.

## Supporting Information Available

Experimental details, NMR spectroscopic analyses, single-crystal X-ray diffraction studies, etc (PDF)
Crystallographic information (CIF)
Crystallographic information (CIF)
Crystallographic information (CIF)
Crystallographic information (CIF)

## Supplementary Material

oc4c00165_si_001.pdf

oc4c00165_si_002.cif

oc4c00165_si_003.cif

oc4c00165_si_004.cif

oc4c00165_si_005.cif
