# Supplementary material for: Chemically Mediated Artificial Electron Transport Chain
Source: ACS Cent Sci. 2024 Apr 10;10(6):1148–55. doi: 10.1021/acscentsci.4c00165 (PMC11212131; doi:10.1021/acscentsci.4c00165)
Supplement: Supplementary file 1 — oc4c00165_si_001.pdf [file oc4c00165_si_001.pdf]

Supplementary Materials for

Chemically Mediated Artificial Electron Transport Chain

Yu-Dong Yang, Qian Zhang, Lhoussain Khrouz, Calvin V. Chau, Jian Yang, Yu-Ying Wang, Christophe Bucher, Graeme Henkelman, Han-Yuan Gong, Jonathan L. Sessler

Corresponding author Email: christophe.bucher@ens-lyon.fr (C.B.), henkelman@utexas.edu (G. H.), hanyuangong@bnu.edu.cn (H.-Y.G.) sessler@cm.utexas.edu (J.L.S.).

The PDF file includes:

Materials and Methods
Calculations of Species Concentrations
Figs. S1 to S40
Tables S1 to S7
References

Materials and Methods

Materials

Deuterated solvents were purchased from Cambridge Isotope Laboratory (Andover, MA). Spectral grade dichloromethane and all reagents were purchased commercially (Fisher) and used without further purification.

Instruments

NMR spectra were recorded on a Varian Mercury 400 instrument. The ^{1}H NMR chemical shifts are referenced to residual solvent signals, namely CD_2Cl_2: δ_H = 5.32 ppm. UV-Vis-NIR spectra were measured on a Varian Cary 5000 spectrophotometer. EPR studies were carried out at room temperature using a Bruker E500 spectrometer operating at X-band (9.4 GHz) with rectangular cavity (ST520). The instrument settings were as follows: microwave power, 69 mW; modulation amplitude, 1 G and 100 KHz modulation frequency. Cyclic voltammetric (CV) studies were carried out on a CH Instrument Model 660D electrochemical system and recorded at a three-electrode configuration consisting of a glassy carbon (working electrode), platinum wire (counter electrode), and an Ag/Ag^+ (non-aqueous reference electrode, 10 mM $AgNO_3$ in CH_3CN) in dichloromethane with 0.1 M tetra-n-butylammonium hexafluorophosphate ($TBAPF_6$) as the supporting electrolyte unless otherwise noted.

Methods

Unless otherwise noted, single crystals used to obtain the X-ray diffraction structures reported in the main text grew as brown prisms or green blocks. The .cif documents, available as separate supporting information files, provide details regarding the specific crystals used for the analysis, along with the structures in question. The data crystals used for the associated single crystal diffraction analyses were cut from clusters of the corresponding crystals and had the approximate dimensions given in the .cif documents. The data were collected on Agilent Technologies SuperNova Dual Source diffractometer using a μ-focus Cu Kα radiation source (λ = 1.5418 Å) with collimating mirror monochromators. Data reduction was performed using the Rigaku Oxford Diffraction's CrysAlisPro V 1.171.41.70a program. The structures were refined by full–matrix least–squares on F^2 with anisotropic displacement parameters for the non–H atoms using SHELXL-2018.[1] The hydrogen atoms were calculated in idealized positions with isotropic displacement parameters set to 1.2 x Ueq of the attached atom (1.5 x Ueq for methyl hydrogen atoms). Definitions used for calculating R(F), Rw(F2) and the goodness of fit, S, are given below and in the .cif documents.[2] Neutral atom scattering factors and values used to calculate the linear absorption coefficient are from the International Tables for X-ray Crystallography (1992).[3] All ellipsoid figures were generated using SHELXTL/PC.[4] Tables of positional and thermal parameters, bond lengths and angles, torsion angles, figures and lists of observed and calculated structure factors are located in the .cif documents available from the Cambridge Crystallographic Centre and may be obtained by quoting ref. numbers 2267375, 2267376, 2267377, and 2267378. The documents contain details of the crystal data, data collection, and structure refinement for each structure.

DFT calculations were performed using the Gaussian 09 program. Geometry optimizations of minima and the intermediates were carried out using the Becke three-parameter hybrid density functional (B3LYP)[5] with the D3 empirical dispersion correction by Grimme[6] *et al.* and the 6-311+G(d,p) basis set[7, 8] for C, H, N, O, F and S atoms, while the def2-TZVPD basis set[9, 10] was used for the heavy halogen atom I, Solvent effects (solvent = dichloromthane) were included in

the single-point energy calculation using SMD model.[11] Frequency analyses were carried out at the same level to verify all of the stationary points as minima (zero imaginary frequencies) or transition states (one imaginary frequency) and to evaluate the zero-point vibrational energies and provide thermal corrections at 298 K.

Calculations of Species Concentrations

With the spectral features for species of **1**, $\mathbf{1^{\bullet+}}$, **2**, $\mathbf{H_32^{\bullet 2+}}$, and $\mathbf{H_32^{+}}$ (as 10 or 5.0 μM solutions) shown in Figs. S2 and S14 over the 250-2000 nm optical window known, the equilibrium concentrations of all species (**1**, $\mathbf{1^{\bullet+}}$, **2**, $\mathbf{H_32^{\bullet 2+}}$, and $\mathbf{H_32^{+}}$) in solution **V** (manuscript Fig. 2B; a mixture of **2** (5.0 μM), 1.0 molar equivalents (eq) **1**, and 40 molar eq of TFA) could be calculated using the Beer-Lambert law, as elaborated below:

As the absorption for 1.0 μM solution of $\mathbf{1^{\bullet+}}$ ($A_{1750\text{ nm}}^{\mathbf{1^{\bullet+}},\,1\,\mu\text{M}}$) is 0.026, and only $\mathbf{1^{\bullet+}}$ absorbs at 1750 nm, the concentration of $\mathbf{1^{\bullet+}}$ ($\boldsymbol{C}_{\mu\text{M}}^{\mathbf{1^{\bullet+}}}$) in solution **V** could be calculated as 4.85 μM using Equat. S1:

$$\boldsymbol{A}_{1750\text{ nm}}^{\mathbf{1^{\bullet+}},\,1\,\mu\text{M}} \times \boldsymbol{C}_{\mu\text{M}}^{\mathbf{1^{\bullet+}}} = \boldsymbol{A}_{1750\text{ nm}}^{\mathbf{1^{\bullet+}}} = \boldsymbol{A}_{1750\text{ nm}}^{\text{Obs}} \quad \text{(Equat. S1)}$$

$$\boldsymbol{C}_{\mu\text{M}}^{\mathbf{1^{\bullet+}}} = \frac{\boldsymbol{A}_{1750\text{ nm}}^{\text{Obs}}}{\boldsymbol{A}_{1750\text{ nm}}^{\mathbf{1^{\bullet+}},\,1\,\mu\text{M}}} = \frac{0.126}{0.026} = 4.85\ (\mu\text{M})$$

For a total concentration in solution **V** of **1** ($\boldsymbol{C}_{\mu\text{M}}^{\mathbf{1},\,\text{Add}}$) of 5.00 μM, the concentration of free **1** ($\boldsymbol{C}_{\mu\text{M}}^{\mathbf{1}}$) could be calculated as 0.15 μM using Equat. S2:

$$\boldsymbol{C}_{\mu\text{M}}^{\mathbf{1}} = \boldsymbol{C}_{\mu\text{M}}^{\mathbf{1},\,\text{Add}} - \boldsymbol{C}_{\mu\text{M}}^{\mathbf{1^{\bullet+}}} = 5.00 - 4.85 = 0.15\ (\mu\text{M}) \quad \text{(Equat. S2)}$$

The absorption intensities of solution **V** at 612 nm and 562 nm reflect the sum of the individual absorptions for **1**, $\mathbf{1^{\bullet+}}$, **2**, $\mathbf{H_32^{\bullet 2+}}$, and $\mathbf{H_32^{+}}$; thus, the total concentration of **2**, $\mathbf{H_32^{\bullet 2+}}$, and $\mathbf{H_32^{+}}$ in this solution reflects the added concentration of **2** ($\boldsymbol{C}_{\mu\text{M}}^{\mathbf{2},\,\text{Add}}$) as obtained using Equat. S3-5:

$$\boldsymbol{C}_{\mu\text{M}}^{\mathbf{2}} + \boldsymbol{C}_{\mu\text{M}}^{\mathbf{H_32^{\bullet 2+}}} + \boldsymbol{C}_{\mu\text{M}}^{\mathbf{H_32^{+}}} = \boldsymbol{C}_{\mu\text{M}}^{\mathbf{2},\,\text{Add}} \quad \text{(Equat. S3)}$$

$$\boldsymbol{A}_{612\text{ nm}}^{\mathbf{2}} + \boldsymbol{A}_{612\text{ nm}}^{\mathbf{H_32^{\bullet 2+}}} + \boldsymbol{A}_{612\text{ nm}}^{\mathbf{H_32^{+}}} + \boldsymbol{A}_{612\text{ nm}}^{\mathbf{1^{\bullet+}}} + \boldsymbol{A}_{612\text{ nm}}^{\mathbf{1}} = \boldsymbol{A}_{612\text{ nm}}^{\text{Obs}} \quad (Equat.S4)$$

$$\boldsymbol{A}_{562\text{ nm}}^{\mathbf{2}} + \boldsymbol{A}_{652\text{ nm}}^{\mathbf{H_32^{\bullet 2+}}} + \boldsymbol{A}_{562\text{ nm}}^{\mathbf{H_32^{+}}} + \boldsymbol{A}_{562\text{ nm}}^{\mathbf{1^{\bullet+}}} + \boldsymbol{A}_{562\text{ nm}}^{\mathbf{1}} = \boldsymbol{A}_{562\text{ nm}}^{\text{Obs}} \quad (Equat.S5)$$

Eqs. (S6-7) can be deduced from Equat. (S3-5);

$$\boldsymbol{A}_{612\text{ nm}}^{\mathbf{2},\,1\,\mu\text{M}} \times \boldsymbol{C}_{\mu\text{M}}^{\mathbf{1}} + \boldsymbol{A}_{612\text{ nm}}^{\mathbf{H_32^{\bullet 2+}},\,1\,\mu\text{M}} \times \boldsymbol{C}_{\mu\text{M}}^{\mathbf{H_32^{\bullet 2+}}} + \boldsymbol{A}_{612\text{ nm}}^{\mathbf{H_32^{+}},\,1\,\mu\text{M}} \times \boldsymbol{C}_{\mu\text{M}}^{\mathbf{H_32^{+}}} + \boldsymbol{A}_{612\text{ nm}}^{\mathbf{1^{\bullet+}}} + \boldsymbol{A}_{612\text{ nm}}^{\mathbf{1}} = \boldsymbol{A}_{612\text{ nm}}^{\text{Obs}}$$

$$(Equat.S6)$$

$$\boldsymbol{A}_{562\text{ nm}}^{\mathbf{2},\,1\,\mu\text{M}} \times \boldsymbol{C}_{\mu\text{M}}^{\mathbf{2}} + \boldsymbol{A}_{562\text{ nm}}^{\mathbf{H_32^{\bullet 2+}},\,1\,\mu\text{M}} \times \boldsymbol{C}_{\mu\text{M}}^{\mathbf{H_32^{\bullet 2+}}} + \boldsymbol{A}_{562\text{ nm}}^{\mathbf{H_32^{+}},\,1\,\mu\text{M}} \times \boldsymbol{C}_{\mu\text{M}}^{\mathbf{H_32^{+}}} + \boldsymbol{A}_{562\text{ nm}}^{\mathbf{1^{\bullet+}}} + \boldsymbol{A}_{562\text{ nm}}^{\mathbf{1}} = \boldsymbol{A}_{562\text{ nm}}^{\text{Obs}}$$

$$(Equat.S7)$$

Where $C^{2,\text{ Add}}_{\mu\text{M}}$ is 5.00 μM, $A^{\text{Obs}}_{612\text{ nm}}$ and $A^{\text{Obs}}_{562\text{ nm}}$ are the observed absorbance of solution **V**. The absorbance at 612 nm and 562 nm for 1.00 μM of **1**, $\mathbf{1^{\bullet +}}$, **2**, $\mathbf{H_3 2^{\bullet 2+}}$, and $\mathbf{H_3 2^{+}}$ are given in Table S1.

Wavelength	$A^{2,\text{ 1 μM}}$	$A^{H_3 2^{\bullet 2+},\text{ 1 μM}}$	$A^{H_3 2^{+},\text{ 1 μM}}$	$A^{1,\text{ 1 μM}}$	$A^{1^{\bullet +},\text{ 1 μM}}$	A^{Obs}
612 nm	0.0259	0.0330	0.5376	0.0023	0.0108	0.3696
562 nm	0.0448	0.1868	0.0611	0.0037	0.0085	0.8852

Table S1.
Absorbance at 612 nm and 562 nm for 1.00 μM of **1**, $\mathbf{1^{\bullet +}}$, **2**, $\mathbf{H_3 2^{\bullet 2+}}$, and $\mathbf{H_3 2^{+}}$.

The absorbance of **1** and $\mathbf{1^{\cdot +}}$ at 612 nm and 562 nm for solution **V** could be calculated as follows:

$$A^{1}_{612\text{ nm}} = A^{1,\text{ 1 μM}}_{612\text{ nm}} \times C^{1}_{\mu\text{M}} = 0.0023 \times 0.15 = 0.0003$$

$$A^{1^{\bullet +}}_{612\text{ nm}} = A^{1^{\bullet +},\text{ 1 μM}}_{612\text{ nm}} \times C^{1^{\bullet +}}_{\mu\text{M}} = 0.0108 \times 4.85 = 0.052$$

$$A^{1}_{562\text{ nm}} = A^{1,\text{ 1 μM}}_{562\text{ nm}} \times C^{1}_{\mu\text{M}} = 0.0037 \times 0.15 = 0.0006$$

$$A^{1^{\bullet +}}_{562\text{ nm}} = A^{1^{\bullet +},\text{ 1 μM}}_{562\text{ nm}} \times C^{1^{\bullet +}}_{\mu\text{M}} = 0.0085 \times 4.85 = 0.041$$

Thus, Equat. (S8-10) were obtained; solving these equations gives the equilibrium concentrations for **2**, $\mathbf{H_3 2^{\bullet 2+}}$, and $\mathbf{H_3 2^{+}}$. The results are shown in Table S2.

$$C^{2}_{\mu\text{M}} + C^{H_3 2^{\bullet 2+}}_{\mu\text{M}} + C^{H_3 2^{+}}_{\mu\text{M}} = 5.00 \quad \text{(Equat. S8)}$$

$$0.026 \times C^{2}_{\mu\text{M}} + 0.033 \times C^{H_3 2^{\bullet 2+}}_{\mu\text{M}} + 0.538 \times C^{H_3 2^{+}}_{\mu\text{M}} = 0.318 \quad \text{(Equat. S9)}$$

$$0.045 \times C^{2}_{\mu\text{M}} + 0.187 \times C^{H_3 2^{\bullet 2+}}_{\mu\text{M}} + 0.061 \times C^{H_3 2^{+}}_{\mu\text{M}} = 0.844 \quad \text{(Equat. S10)}$$

Eqs S8-10 could also be used to calculate the equilibrium concentrations and percentages of the species of interest (**1**, $\mathbf{1^{\bullet +}}$, **2**, $\mathbf{H_3 2^{\bullet 2+}}$, and $\mathbf{H_3 2^{+}}$) in solution **IV** (manuscript Fig. 2B) and in other solutions containing these species.

solution		**2**	**$H_32^{\bullet 2+}$**	**H_32^{+}**	**1**	**$1^{\bullet +}$**
**V** **2** (5 µM) + 1.0 eq **1** + 40 eq TFA	Equilibrium concentration	0.36 µM	4.33 µM	0.31 µM	0.15 µM	4.85 µM
	Equilibrium percentage	7.2 %	86.6%	6.2 %	3.0 %	97 %
**IV** **2** (5 µM) + 2.0 eq **1** + 40 eq TFA	Equilibrium concentration	0.38 µM	1.73 µM	2.89 µM	2.15 µM	7.85 µM
	Equilibrium percentage	7.6 %	34.6 %	57.8 %	21.5 %	78.5 %

Table S2.

Equilibrium concentrations and percentages of species of interest (**1**, **$1^{\bullet +}$**, **2**, **$H_32^{\bullet 2+}$**, and **H_32^{+}**) in solutions **V** and **IV** (manuscript Fig. 2B).

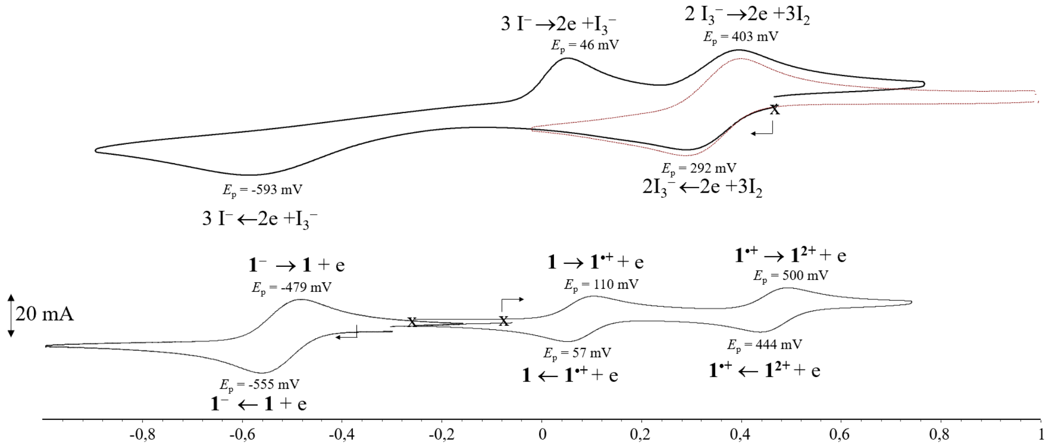

Fig. S1.

Voltammetric curves recorded on a glassy carbon working electrode (∅ = 3mm, 100 mV/s, *E* in volts *vs* $E_{ref}[Ag^+/Ag]$, N_2 atmosphere) for dichloroethane (DCE) solutions (+ 0.1 M in tetrabutylammonium bis-trifluoromethanesulfonimidate (TBATFSI)) of I_2 (top trace, 1.0 mM) and **1** (lower trace, 1.0 mM).

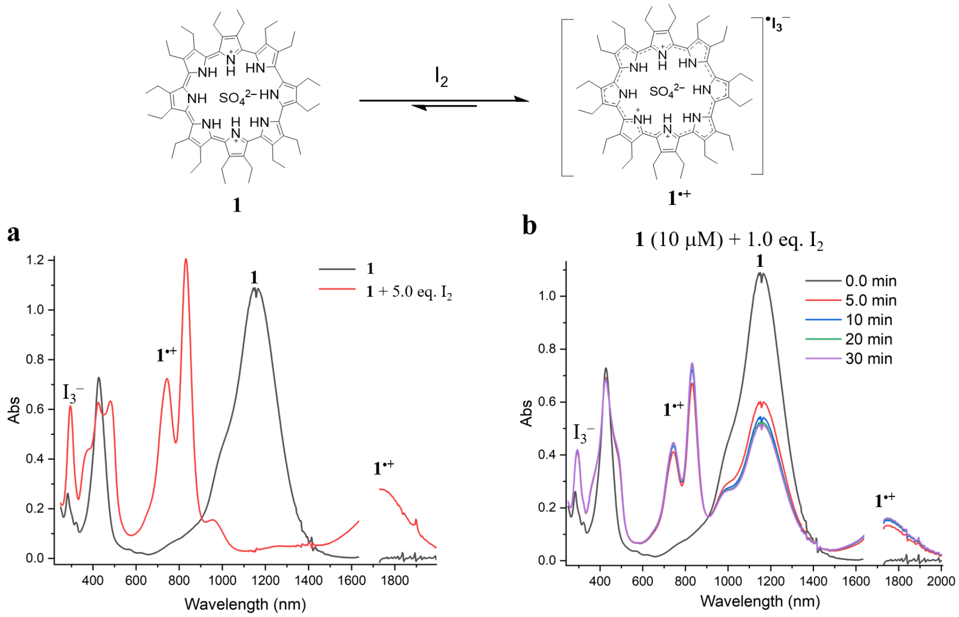

Fig. S2.

a, UV-Vis-NIR spectra of **1** (10 μM) recorded in the absence and presence of 5.0 molar eq of I_2. **b**, Time dependent UV-Vis-NIR spectra of **1** (10 μM) recorded in the presence of 1.0 molar eq of I_2 (CH_2Cl_2, 1 cm optical path).

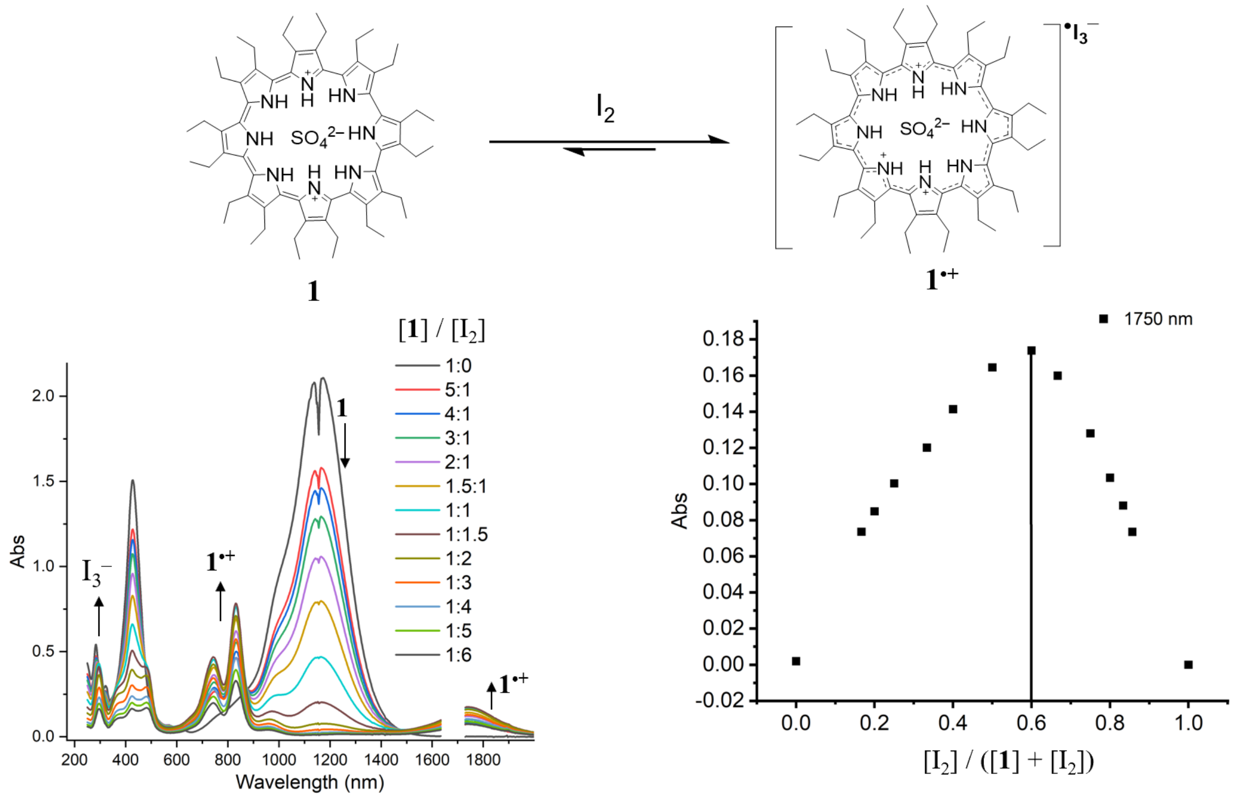

Fig. S3.

UV-Vis-NIR spectroscopic Job plot ([**1**] + [I_2] = 20 μM) corresponding to the interaction between **1** and I_2, as determined by monitoring the absorption change of 1750 nm. The maximum value of the plot falls at 0.6, a finding consistent with the formation of a complex with 2:3 (host/guest) binding stoichiometry[12] (CH_2Cl_2, 1 cm optical path).

a

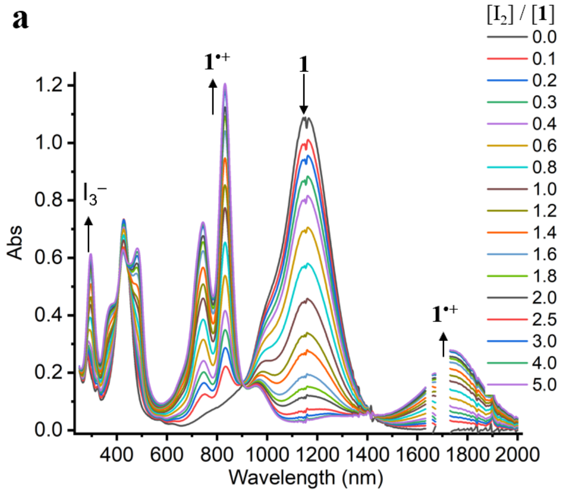

b

$$[\mathbf{1}] + [I_2] \xrightleftharpoons{K_{a1}} [\mathbf{1}^{\bullet+}\bullet I_2^-] \quad \text{(Eq S11)}$$

$$[\mathbf{1}^{\bullet+}\bullet I_2^-] + [I_2] \xrightleftharpoons{K_{a2}} [\mathbf{1}^{\bullet+}\bullet I_2^-\bullet I_2] \quad \text{(Eq S12)}$$

$$[\mathbf{1}^{\bullet+}\bullet I_2^-] + [\mathbf{1}^{\bullet+}\bullet I_2^-\bullet I_2] \xrightleftharpoons{K_{a3}} 2[\mathbf{1}^{\bullet+}\bullet I_3^-] \quad \text{(Eq S13)}$$

c

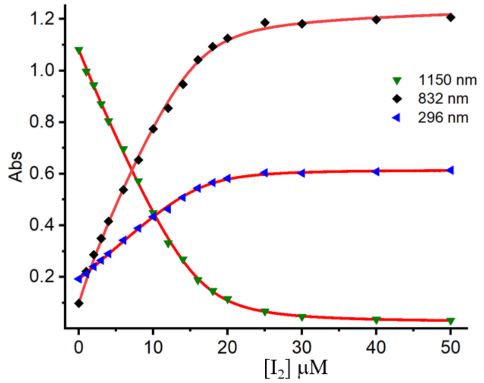

Fig. S4.

a, UV-Vis-NIR spectroscopic titration of **1** ([host] = 10 μM) with I_2 (guest) (CH_2Cl_2, 1 cm optical path). **b**, Equilibrium equations corresponding to the 2:3 (host/guest) binding between **1** and I_2. **c**, UV-Vis-NIR absorbance at 1150 nm, 832 nm, and 296 nm binding isotherms corresponding to the interaction between **1** and I_2. The absorption from 250 nm to 1400 nm were used to calculate the K_{a1} ((2.5 ± 0.2) × 10^5 M^{-1}), K_{a2} ((1.0 ± 0.1) × 10^5 M^{-1}), K_{a3} ((3.5 ± 0.3) × 10^6 M^{-1}), using the Hyperquad 2003 program.[13, 14] The red lines show the non-linear curve fits of the experimental data to the appropriate equation.

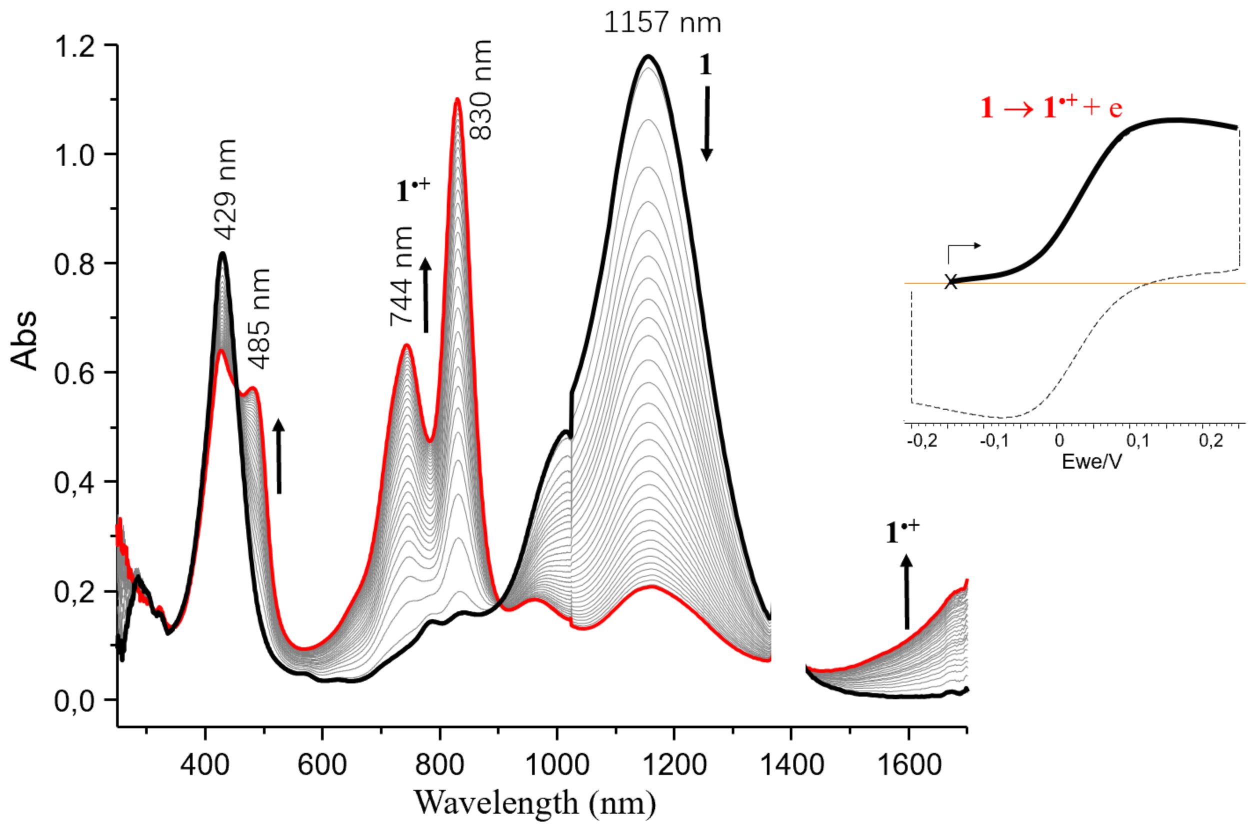

Fig. S5.

UV-Vis-NIR absorption spectra recorded for a DCE solution of **1** (10 µM in DCE + 0.1 M in (TBATFSI)) in a thin layer spectroelectrochemistry cell (1 mm, Pt grid) as the potential of the working electrode is swept linearly from – 0.15 V to + 0.25 V at 10 mV/s^{-1}, followed by 10 minutes of potentiation electrolysis at + 0.25 V. The red and black dark lines correspond to the beginning and end of the experiment. The CV curve recorded during this experiment is shown as an inset.

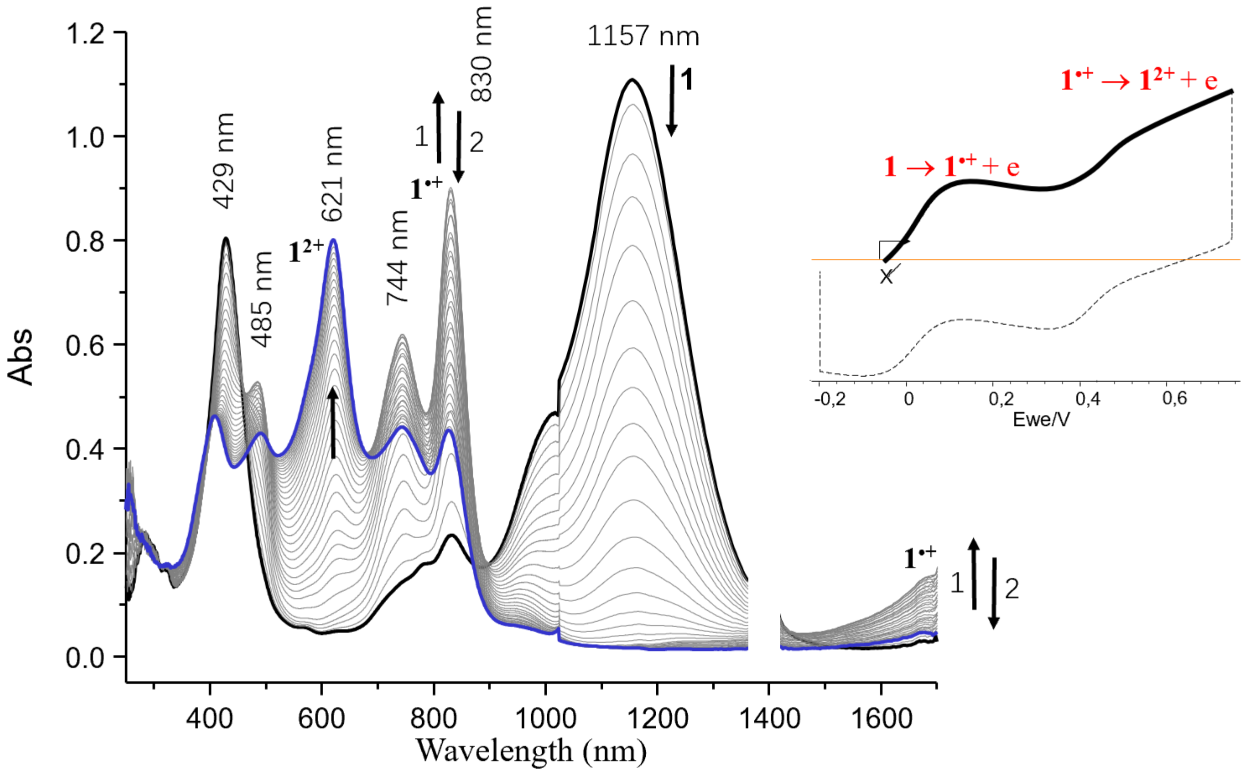

Fig. S6.

UV-Vis-NIR absoption spectra recorded for a DCE solution of **1** (10 µM in DCE + 0.1 M in TBATFSI) in a thin layer spectroelectrochemistry cell (1 mm, Pt grid) when the potential of the working electrode is swept linearly from – 0.1 V to + 0.75 V at 10 mV/s^{-1}, followed by 10 minutes of potentiostatic electrolysis at + 0.75 V. The blue and black dark lines correspond to the beginning and end of the experiment. The CV curve recorded during this experiment is shown as an inset.

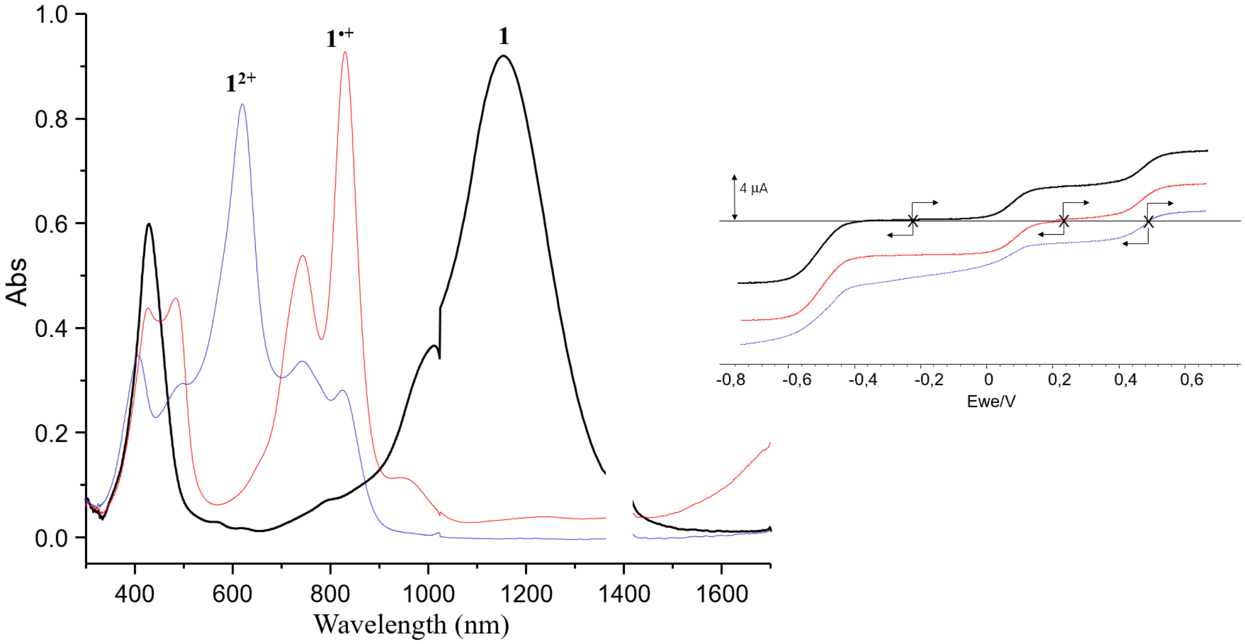

Fig. S7.

UV-Vis-NIR absorption spectra (path length = 1 mm) and voltammetric curves (rotating disk electrode (RDE), glassy carbon WE ∅ = 3mm, 500 rd/s, 10 mV/s) recorded before (black lines) and after exhaustive one-electron (red lines) or two electron (blue lines) oxidation of **1**. Electrolyses were conducted using a platinum working electrode whose potential was fixed at Eap = + 0.275 V then + 0.65 V (10 mL, t = ~1 h, DCE + 0.1 M TBATFSI, glovebox N_2, divided cell).

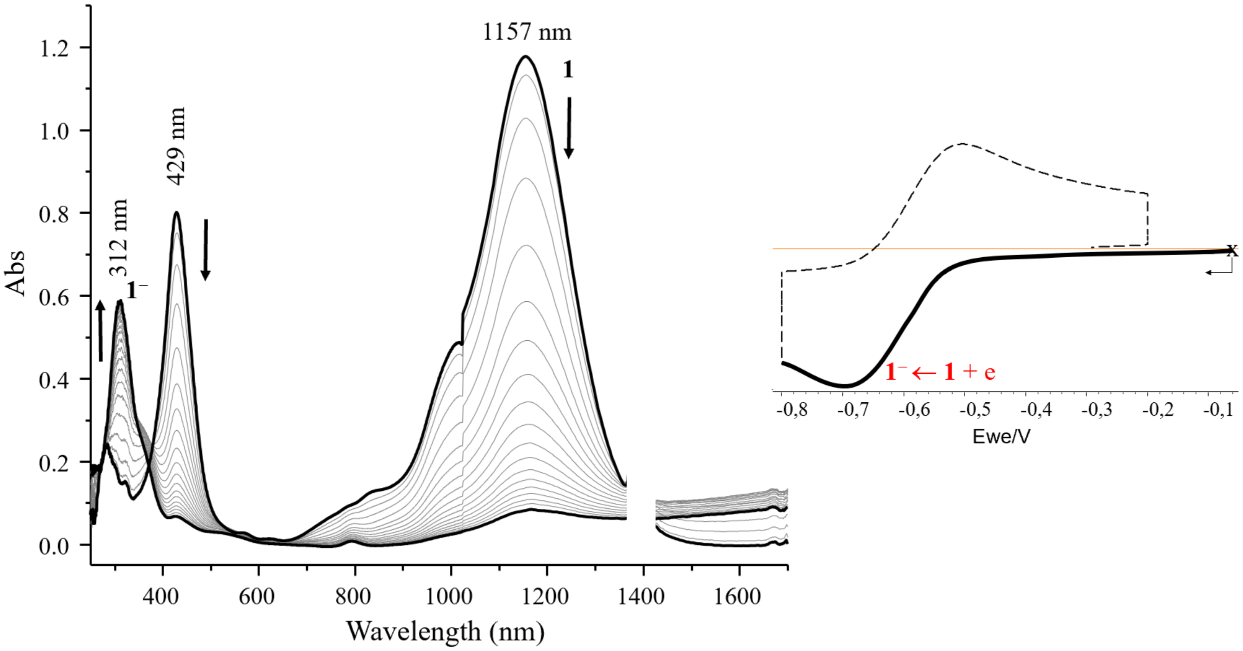

Fig. S8.

UV-Vis-NIR absoption spectra recorded for a DCE solution of **1** (10 μM in DCE + 0.1 M in TBATFSI) in a thin layer spectroelectrochemistry cell (1 mm, Pt grid) as the potential of the working electrode is swept linearly from – 0.1 V to – 0.8 V at 10 mV/s^{-1}, followed by 10 minutes of potentiostatic electrolysis at – 0.8 V. The CV curve recorded during this experiment is shown as an inset.

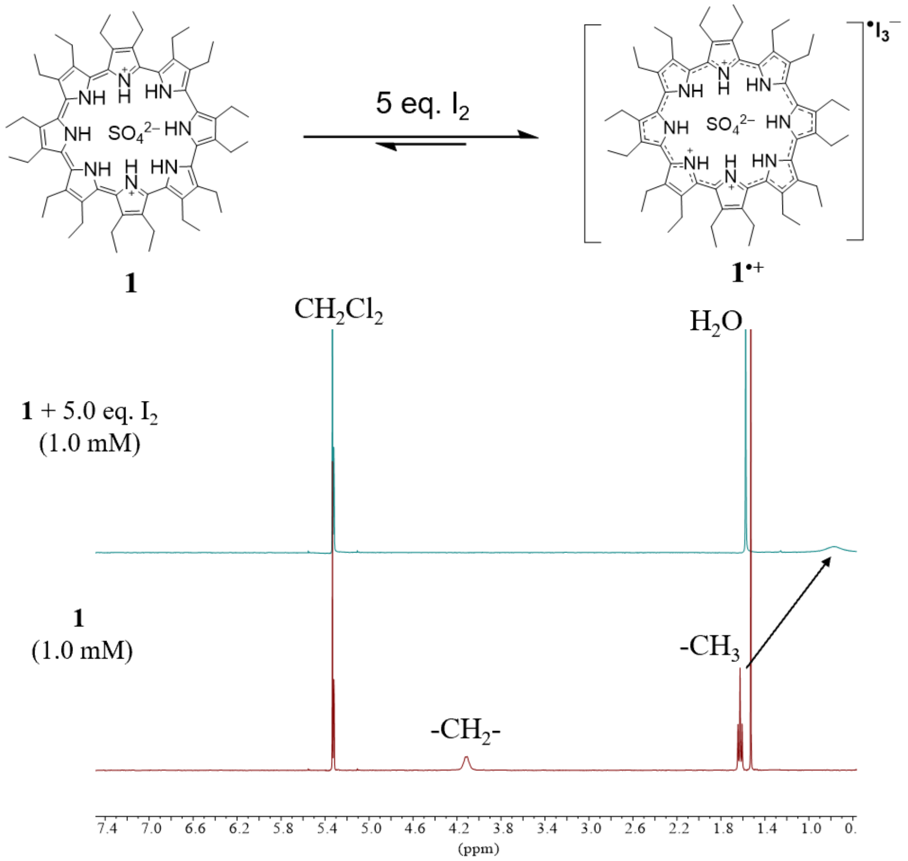

Fig. S9.

Expansion of the ^{1}H NMR spectra of **1** (1.0 mM) recorded in the absence and presence of 5.0 molar eq of I_2 in CD_2Cl_2 at 298 K (400 MHz).

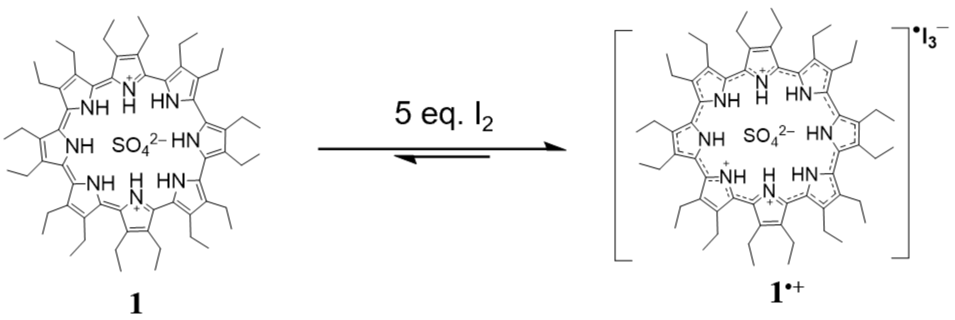

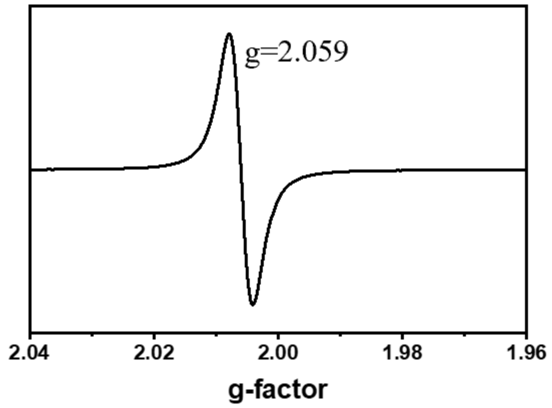

Fig. S10.
EPR spectrum of **1** (0.1 mM) recorded in the presence of 5.0 molar eq of I_2 in CH_2Cl_2 at 298 K.

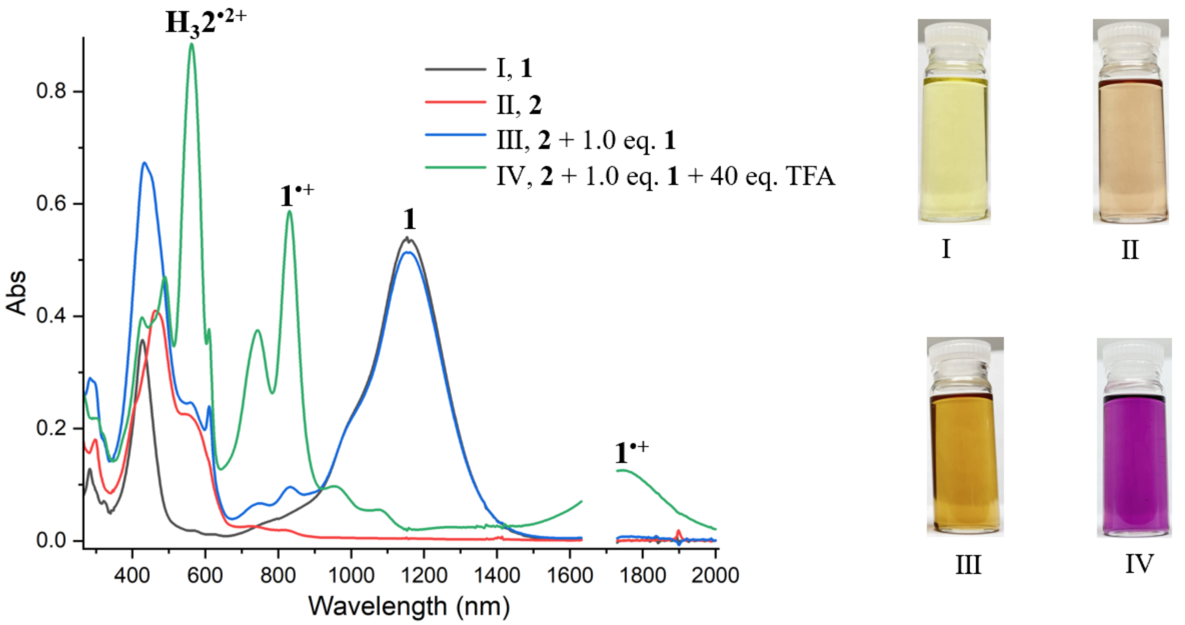

Fig. S11.

UV-Vis-NIR spectra and photographs of various samples: **I**) **1** (5.0 μM), **II**) **2** (5.0 μM), **III**) a mixture of **2** (5.0 μM) and 1.0 molar eq **1**, **IV**) a mixture of **2** (5.0 μM), 1.0 molar eq **1**, and 40 molar eq of TFA (CH_2Cl_2, 1 cm optical path).

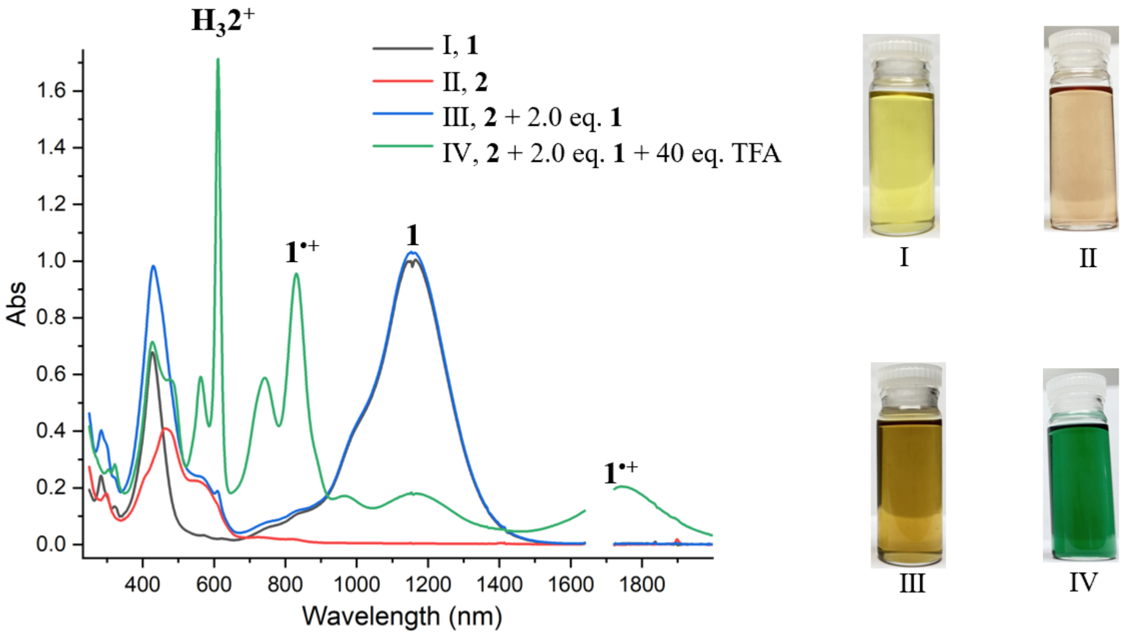

Fig. S12.

UV-Vis-NIR spectra and photographs of various sample: **I**) **1** (10 μM), **II**) **2** (5.0 μM), **III**) a mixture of **2** (5.0 μM) and 2.0 molar eq **1**, **IV**) a mixture of **2** (5.0 μM), 2.0 molar eq **1**, and 40 molar eq of TFA (CH_2Cl_2, 1 cm optical path).

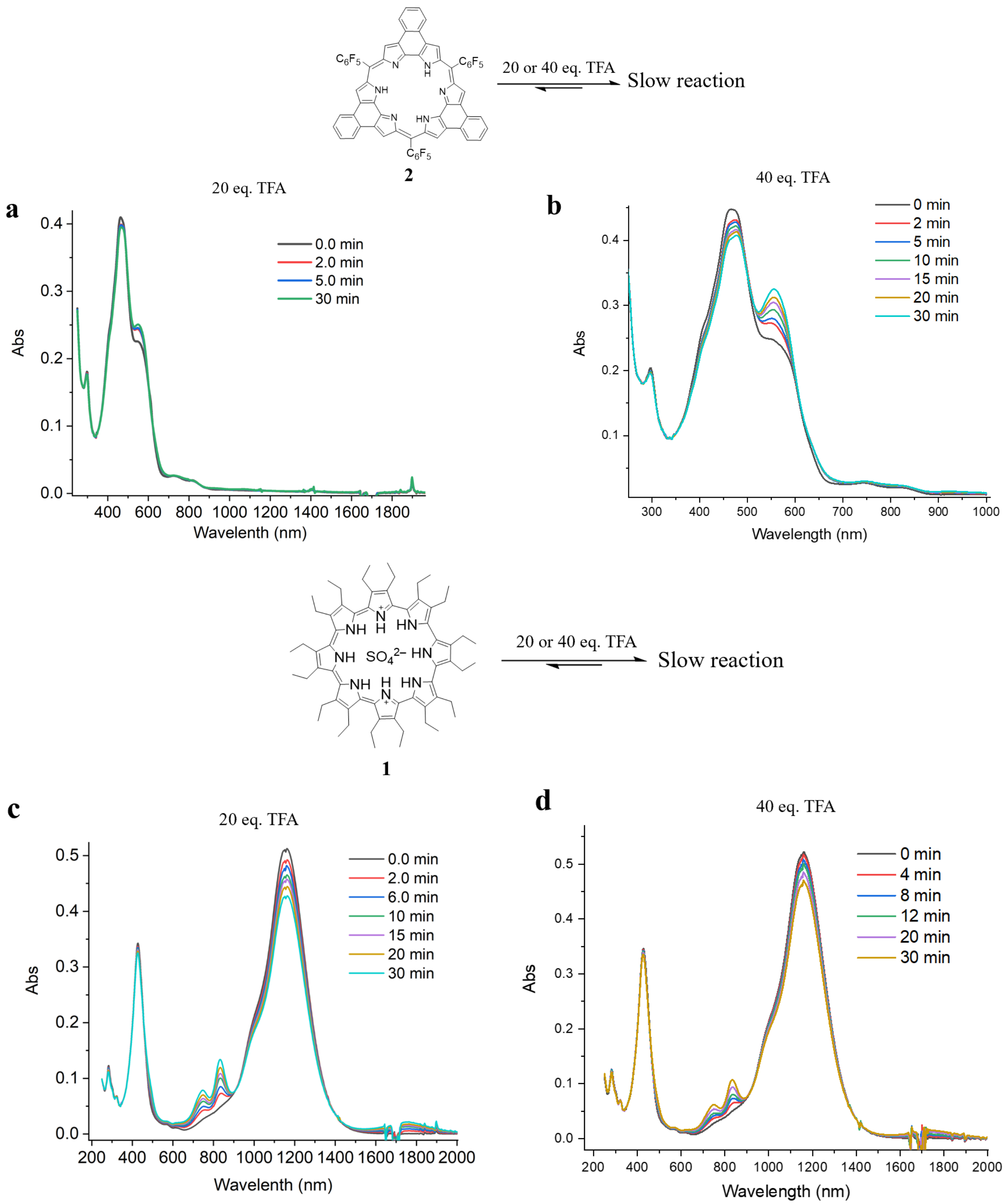

Fig. S13.

Time dependent UV-Vis-NIR spectral studies of **2** (5.0 μM, **a** and **b**) and **1** (5.0 μM, **c** and **d**) recorded in the presence of 20 or 40 molar eq of TFA (CH_2Cl_2, 1 cm optical path).

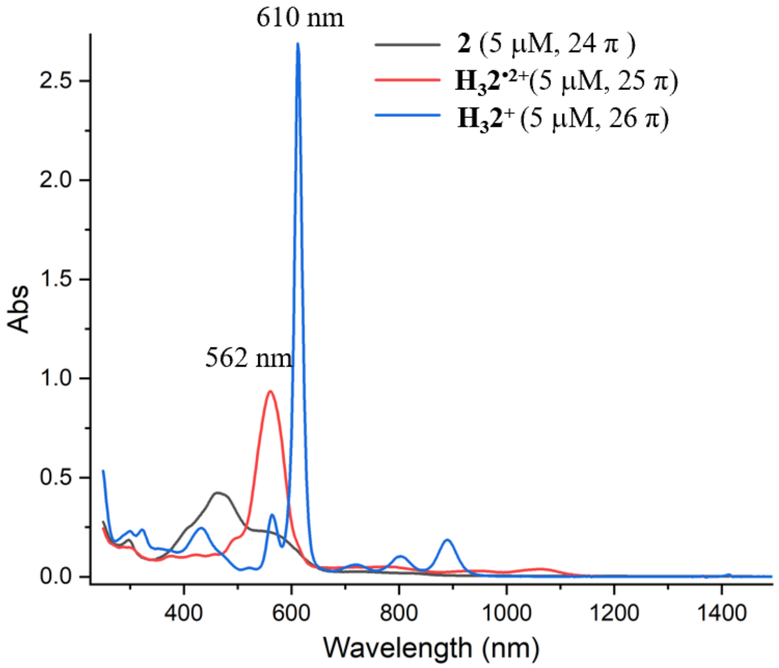

Fig. S14.

UV-Vis-NIR spectra for **2** (5.0 μM), $\mathbf{H_32^{•2+}}$ (5.0 μM, prepared from a solution of **2** and 50 molar eq of HCl), and $\mathbf{H_32^{+}}$ (5.0 μM, prepared from a solution of **2** and 50 molar eq of HI) (CH_2Cl_2, 1 cm optical path).

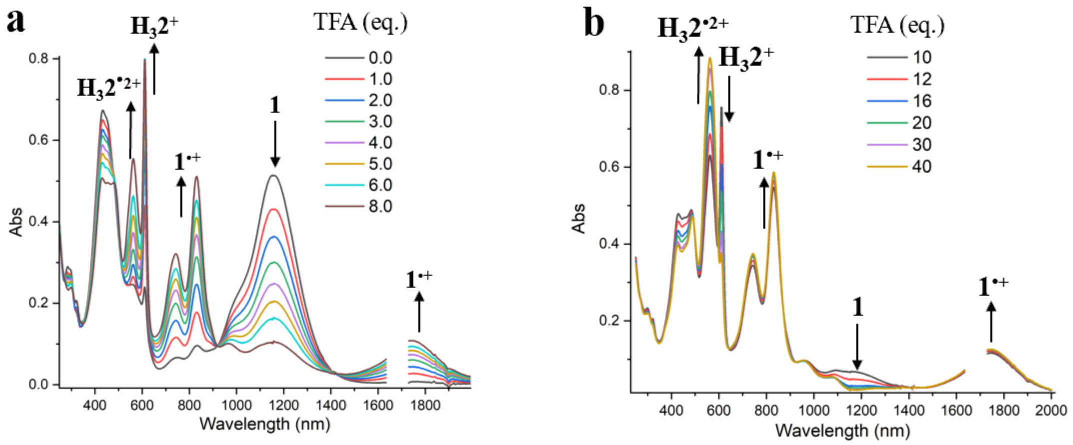

c

$$[\mathbf{1}] + [\mathbf{2}] + 3[CF_3CO_2H] \rightleftharpoons [\mathbf{1}^{\bullet +}\bullet CF_3CO_2^{-}] + [\mathbf{H_32}^{\bullet 2+}\bullet(CF_3CO_2^{-})_2] \quad \text{(Equat. S14)}$$

$$[\mathbf{1}] + [\mathbf{H_32}^{\bullet 2+}\bullet(CF_3CO_2^{-})_2] \rightleftharpoons [\mathbf{1}^{\bullet +}\bullet CF_3CO_2^{-}] + [\mathbf{H_32}^{+}\bullet CF_3CO_2^{-}] \quad \text{(Equat. S15)}$$

$$[\mathbf{2}] + 3[CF_3CO_2H] + [\mathbf{H_32}^{+}\bullet CF_3CO_2^{-}] \rightleftharpoons 2[\mathbf{H_32}^{\bullet 2+}\bullet(CF_3CO_2^{-})_2] \quad \text{(Equat. S16)}$$

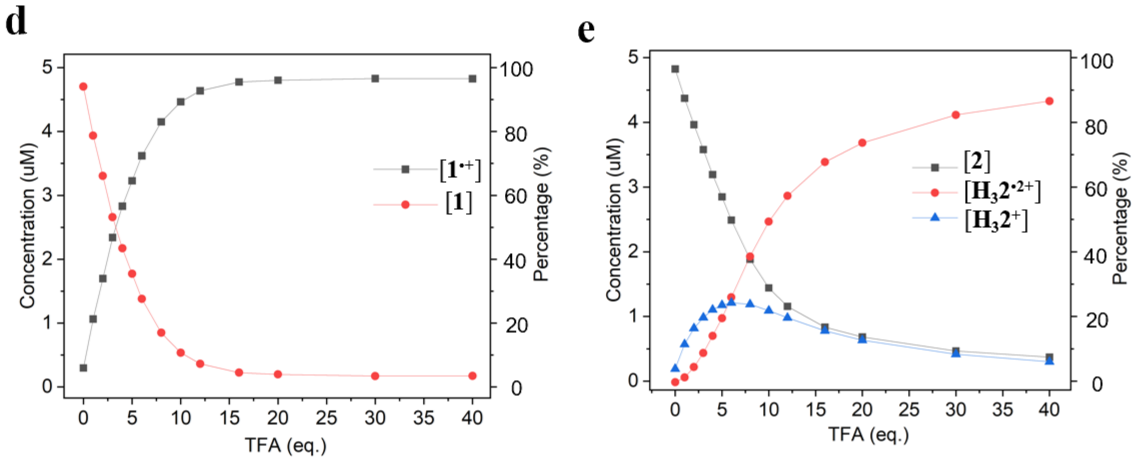

Fig. S15.

a and **b**, UV-Vis-NIR spectroscopic titrations involving the addition of TFA into a mixture of **2** (5.0 μM) and 1.0 molar eq of **1** (CH_2Cl_2, 1 cm optical path). **c**, Proposed equilibrium reactions corresponding to the reactions between **1**, **2**, and TFA. **d** and **e**, Plot of concentrations and percentages of **1**, $\mathbf{1}^{\bullet +}$, **2**, $\mathbf{H_32}^{\bullet 2+}$, and $\mathbf{H_32}^{+}$ vs the molar eq of added TFA from absorbance changes in the titration studies in **a** and **b**. Note the lines have been added to aid in visualization.

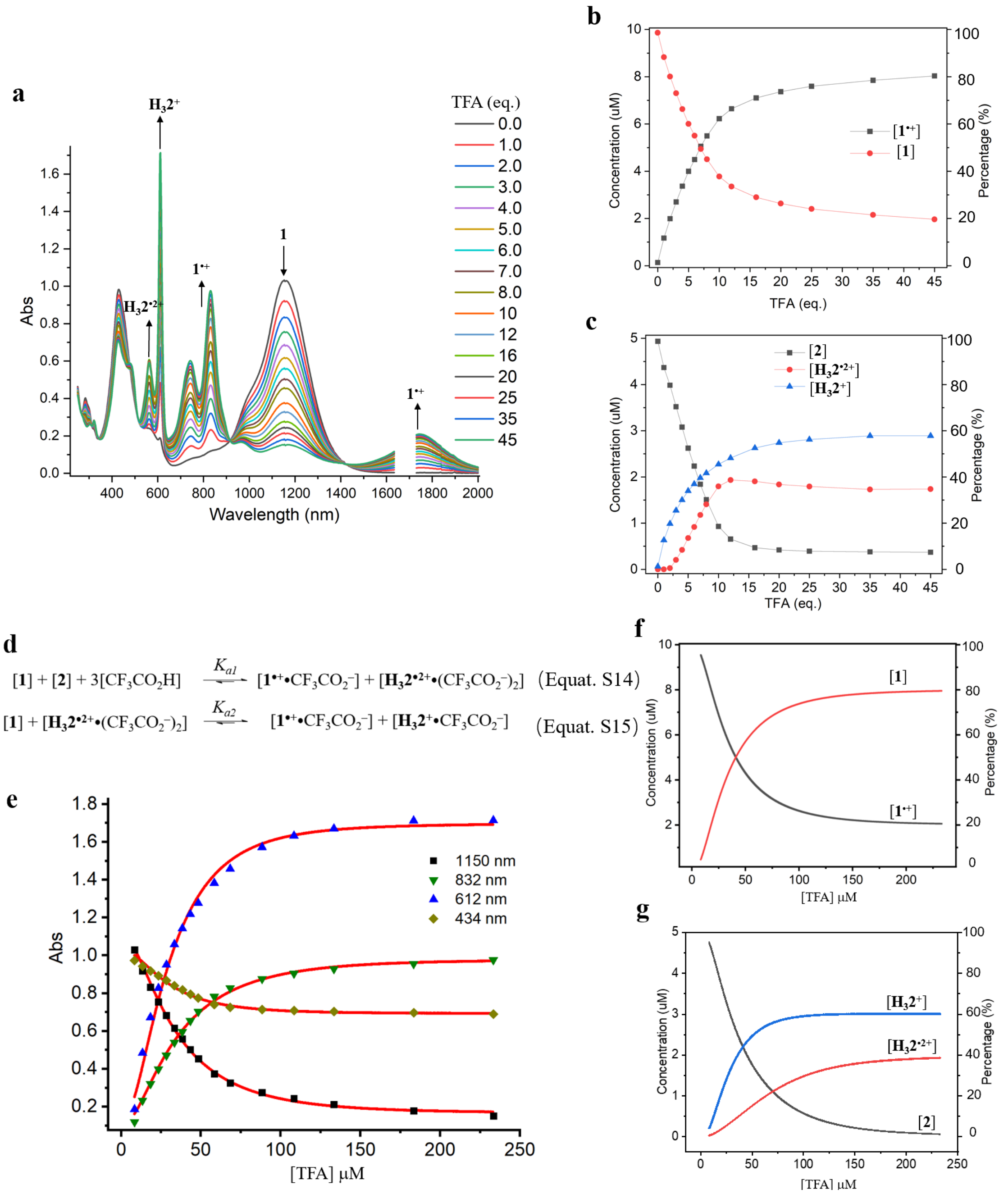

Fig. S16.

a, UV-Vis-NIR spectroscopic titration involving the addition of TFA into a mixture of **2** (5.0 µM) and 2.0 molar eq of **1** (CH_2Cl_2, 1 cm optical path). **b** and **c**, Plot of concentrations and percentages of **1**, $\mathbf{1^{\bullet+}}$, **2**, $\mathbf{H_32^{\bullet2+}}$, and $\mathbf{H_32^{+}}$ vs the molar eq of added TFA from absorbance changes in the titration studies in (**a** and **b**). **d**, Proposed equilibrium reactions corresponding to the interactions between **1**, **2**, and TFA. **e**, UV-Vis-NIR absorbance intensity at 1150 nm, 832 nm, 612 nm, and 434 nm vs added TFA and binding isotherms corresponding to the interactions between between **1**, **2**, and TFA (Note: The starting point corresponds to 8.3 µM TFA used to create the mildly acidic initial CH_2Cl_2 solution). Changes in the absorption intensities over the 250 nm to 1400 nm

spectral region were used to calculate K_{a1} ($(1.5 \pm 0.2) \times 10^{18}$ M^{-2}) and K_{a2} ($(7.6 \pm 0.5) \times 10^{5}$ M) using the Hyperquad 2003 program.[13, 14] The red lines show the non-linear curve fit of the experimental data to the appropriate equation. **f** and **g**, Plot of concentrations and percentages of **1**, $\mathbf{1^{\bullet+}}$, **2**, $\mathbf{H_3 2^{\bullet 2+}}$, and $\mathbf{H_3 2^{+}}$ vs added TFA from **d** and **e**.

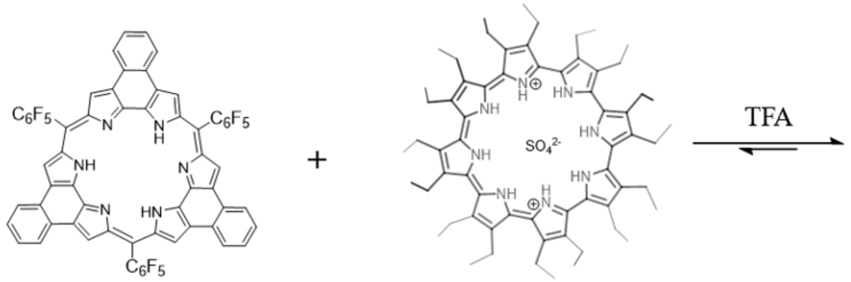

2 **1**

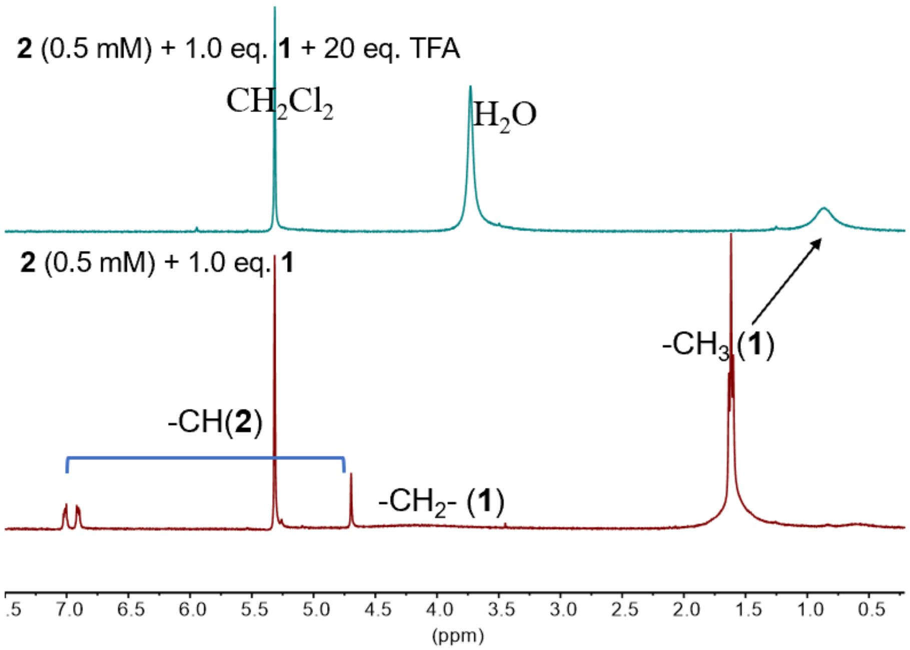

Fig. S17.

Expanded view of the ^{1}H NMR spectra of a mixture **2** (1.0 mM) and 1.0 molar eq of **1** recorded in the absence and presence of 20 molar eq of TFA in CD_2Cl_2 at 298 K (400 MHz).

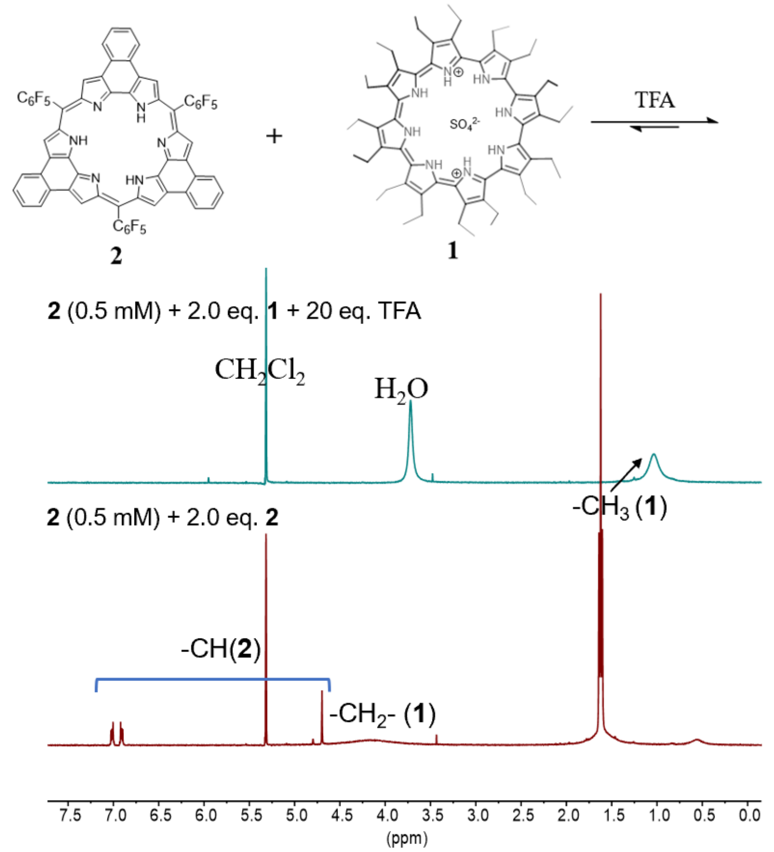

Fig. S18.
Expanded view of the ^{1}H NMR spectra of a mixture **2** (1.0 mM) and 2.0 molar eq of **1** recorded in the absence and presence of 20 molar eq of TFA in CD_2Cl_2 at 298 K (400 MHz).

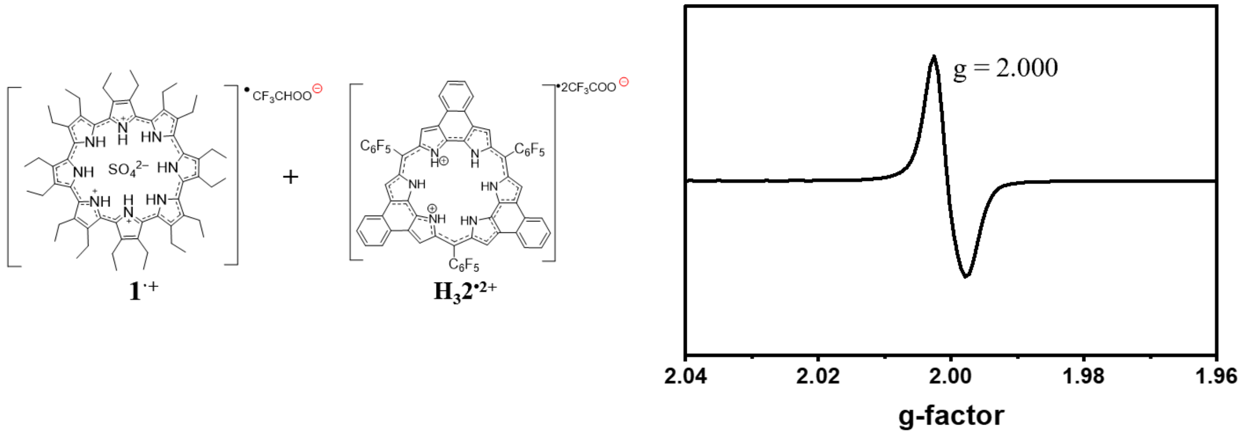

Fig. S19.
ESR spectrum of a mixture of **2** (0.1 mM), 1.0 molar eq of **1**, and 20 molar eq of TFA in CH_2Cl_2 at 298 K.

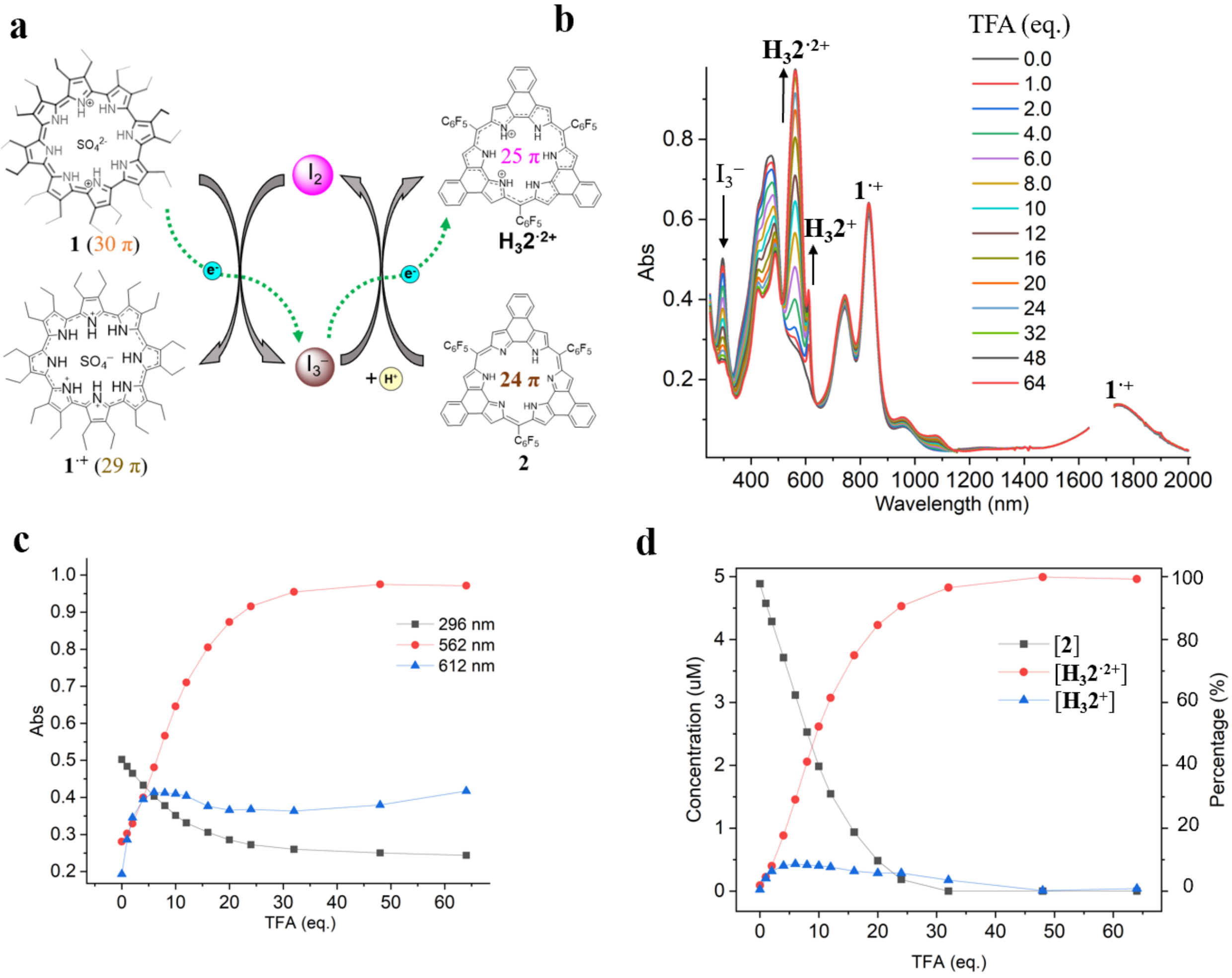

Fig. S20.

a, Schematic representations of the electron transfer from **1** to I_2, and further to give $\mathbf{H_32^{\bullet 2+}}$. **b**, UV-Vis-NIR spectral titrations of TFA into a mixed solution of **2** (5.0 μM), 1.0 molar eq of **1**, and 5.0 molar eq of I_2 (CH_2Cl_2, 1 cm optical path). **c**, Absorption spectral changes at 296 nm, 562 nm, and 612 nm seen upon titration with TFA. **d**, Plot of concentrations and percentages of **2**, $\mathbf{H_32^{\bullet 2+}}$, and $\mathbf{H_32^{+}}$ vs the added molar eq of TFA based on the titration studies shown in (**b**). Note the lines have been added to aid in visualization.

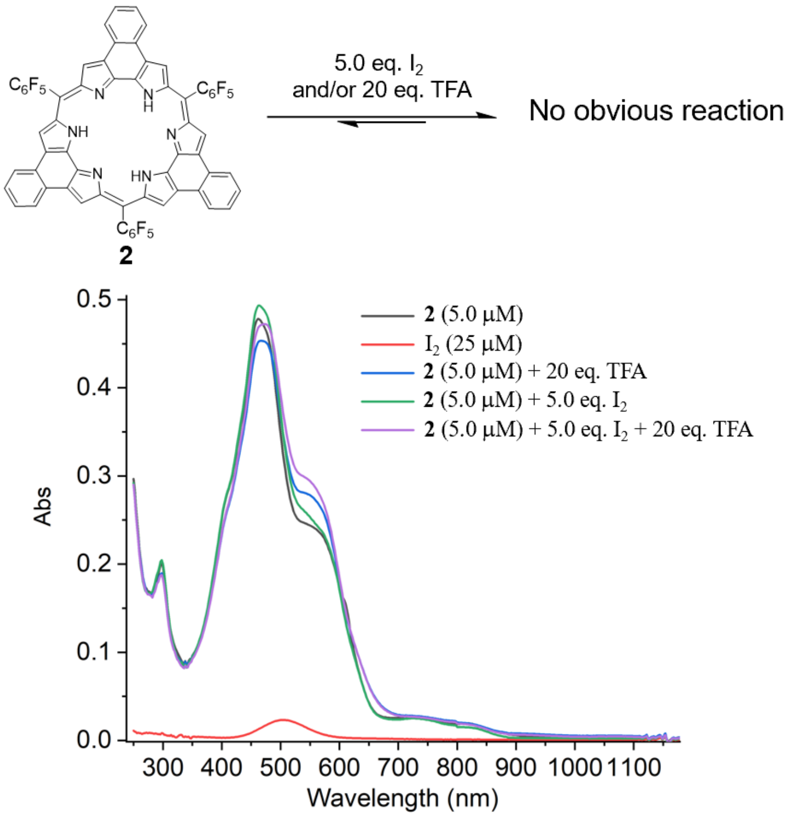

Fig. S21.

UV-Vis-NIR spectra of **2** (5.0 μM), I_2 (25 μM), a mixture of **2** (5.0 μM) and 20 molar eq TFA, a mixture of **2** (5.0 μM), 5.0 molar eq I_2, and a mixture of **2** (5.0 μM), 5.0 molar eq I_2, and 20 molar eq TFA (CH_2Cl_2, 1 cm optical path).

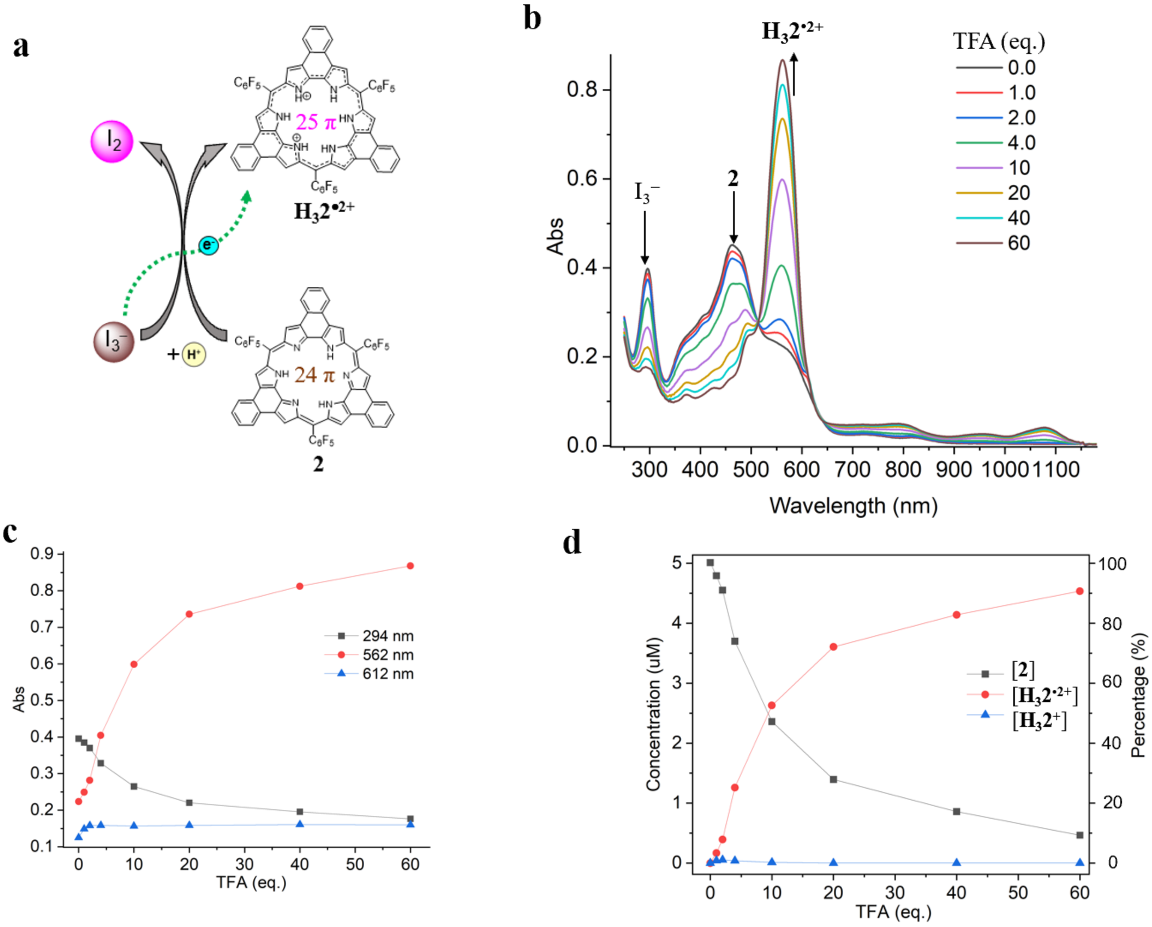

Fig. S22.

a, Schematic representation of the electron transfer from I_3^- to give **$H_3 2^{\bullet 2+}$**. **b**, UV-Vis-NIR spectral titrations of TFA into a mixed solution of **2** (5.0 μM), I_2 (3.5 μM), and 1.0 molar eq of $TBA^+\bullet I_3^-$ (CH_2Cl_2, 1 cm optical path). **c**, Absorption changes at 296 nm, 562 nm, and 612 nm seen upon titration with TFA. **d**, Plot of concentrations and percentages of **2**, **$H_3 2^{\bullet 2+}$**, and **$H_3 2^+$** vs the added molar eq of TFA based on the titration studies shown in (**b**). Note the lines have been added to aid in visualization.

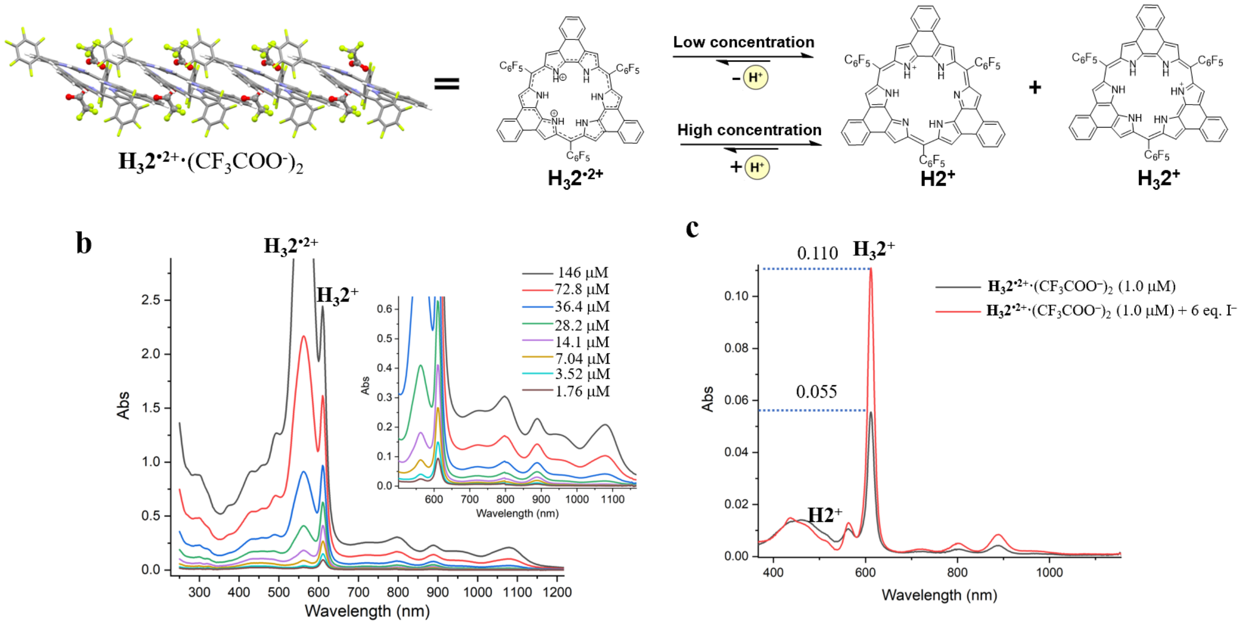

Fig. S23.

a, Schematic representation showing the disproportionate reaction of $\mathbf{H_32^{\bullet 2+}}$ to give a 1:1 mixture of **2** and $\mathbf{H_32^{+}}$. **b**, Concentration dependent UV-Vis-NIR spectra of crystalline $\mathbf{H_32^{\bullet 2+}}\bullet(CF_3CO_2^{-})_2$ dissolved in CH_2Cl_2. **c**, UV-Vis-NIR spectra of crystalline $\mathbf{H_32^{\bullet 2+}}\bullet(CF_3CO_2^{-})_2$ dissolved in CH_2Cl_2 (1.0 µM) followed by the addition of 6 eq of $TBA^{+}\bullet I^{-}$ (conditions that serve to promote the disproportion of $\mathbf{H_32^{\bullet 2+}}$ into $\mathbf{H2^{+}}$ and $\mathbf{H_32^{+}}$) (CH_2Cl_2, 0.2 cm optical path).

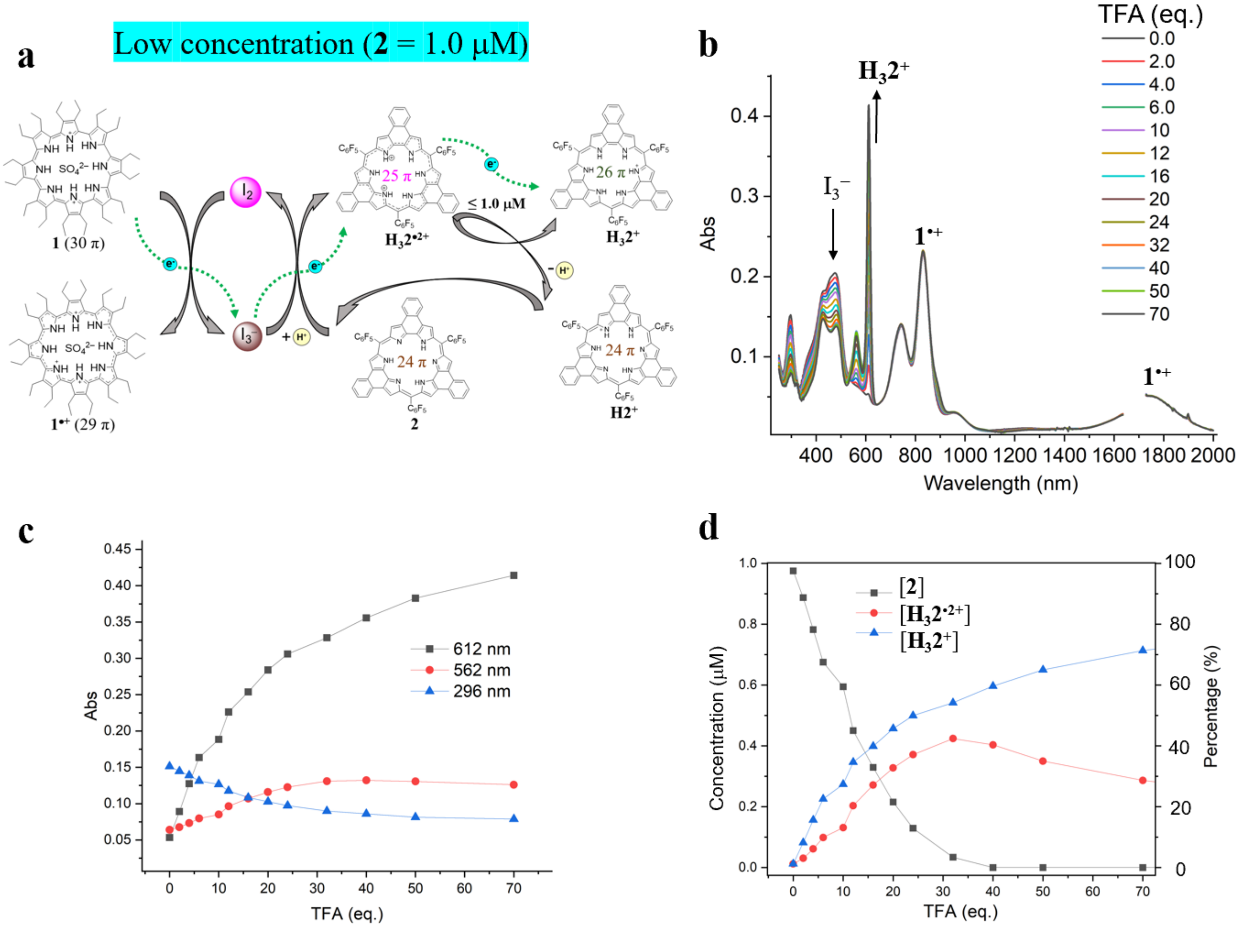

Fig. S24.

a, Schematic representation of the electron transport from **1** to I_2 and further to give $\mathbf{H_32^{\bullet 2+}}$ and $\mathbf{H_32^{+}}$. **b**, UV-Vis-NIR spectral titrations of TFA into a mixed solution of **2** (1.0 μM), 2.0 molar eq of **1**, and 10 molar eq of I_2 (CH_2Cl_2, 1 cm optical path). **c**, Absorption changes at 296 nm, 562 nm, and 612 nm upon titration with TFA. **d**, Plot of concentration and percentage of **2**, $\mathbf{H_32^{\bullet 2+}}$, and $\mathbf{H_32^{+}}$ vs the number of added molar eq of TFA from the data in (**b**). Note the lines have been added to aid in visualization.

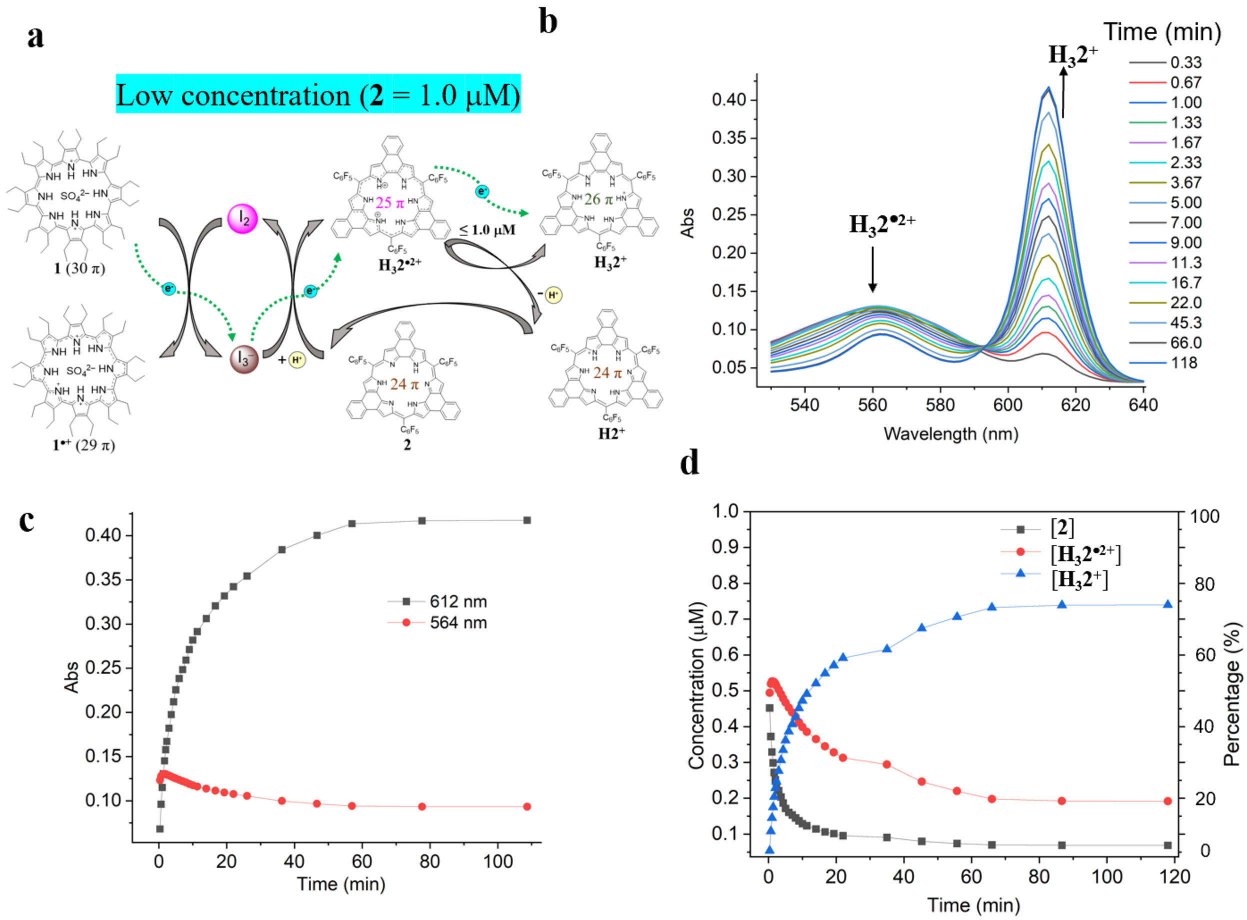

Fig. S25.

a, Schematic representation of the electron transport events involving **1** and I_2 to give first I_3^- and then to give $\mathbf{H_32^{\bullet 2+}}$ and $\mathbf{H_32^+}$. **b**, Time dependent UV-Vis-NIR spectra of a mixed solution of **2** (1.0 μM), 2.0 molar eq of **1**, 10 molar eq of I_2, and 40 molar eq of TFA. **c**, Time dependent absorption changes at 564 nm and 612 nm. **d**, Plot of the concentrations and percentages of **2**, $\mathbf{H_32^{\bullet 2+}}$, and $\mathbf{H_32^+}$ vs time. Note the lines have been added to aid in visualization.

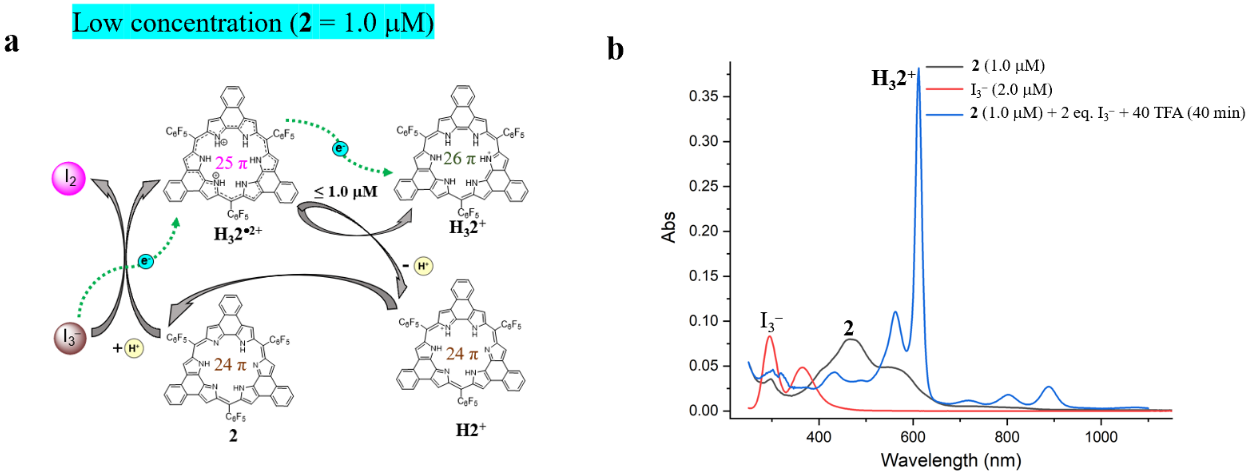

Fig. S26.

a, Schematic representation of the proposed electron transfer events involving I_3^- to give **$H_3 2^{\bullet 2+}$** and **$H_3 2^+$**. **b**, UV-Vis-NIR spectra of **2** (1.0 uM), $TBA^+ \bullet I_3^-$ (2.0 µM), and a mixture of **2** (1.0 µM), 2.0 molar eq of $TBA^+ \bullet I_3^-$, and 40 molar eq of TFA (CH_2Cl_2, 1 cm optical path).

High concentration (**2** = 10 µM)

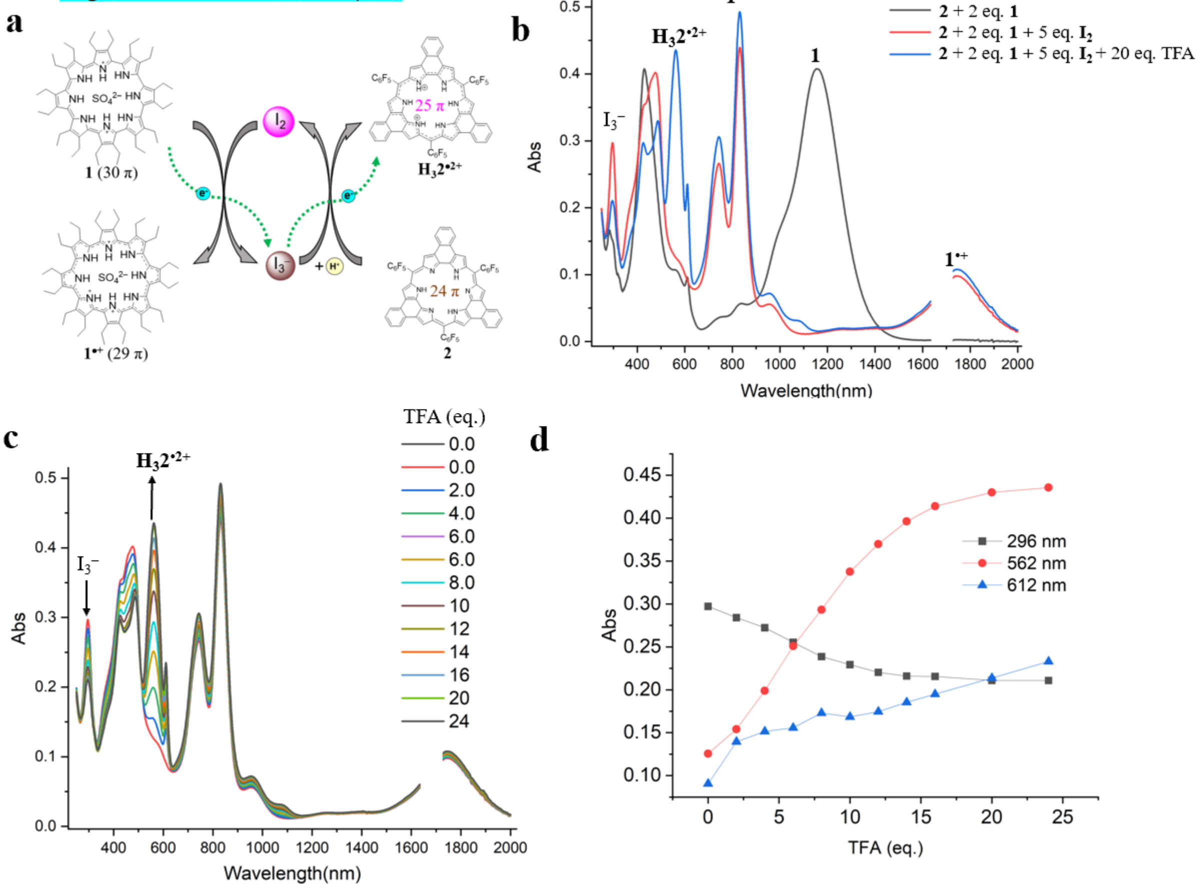

Fig. S27.

a, Schematic representation of the electron transport from **1** to I_2 and further to give $\mathbf{H_32^{\bullet 2+}}$. **b**, UV-Vis-NIR spectra of mixed solutions of **2** (10 µM) and 2.0 molar eq **2**, recorded upon the stepwise addition of 5.0 molar eq of I_2 and 20 eq of TFA. **c**, UV-Vis-NIR spectral titration of TFA into a mixture of **2** (10 µM), 2.0 molar eq **2**, and 5.0 molar eq of I_2 (CH_2Cl_2, 0.2 cm optical path). **d**, Absorption changes at 296 nm, 562 nm, and 612 nm seen upon titration with TFA. Note the lines have been added to aid in visualization.

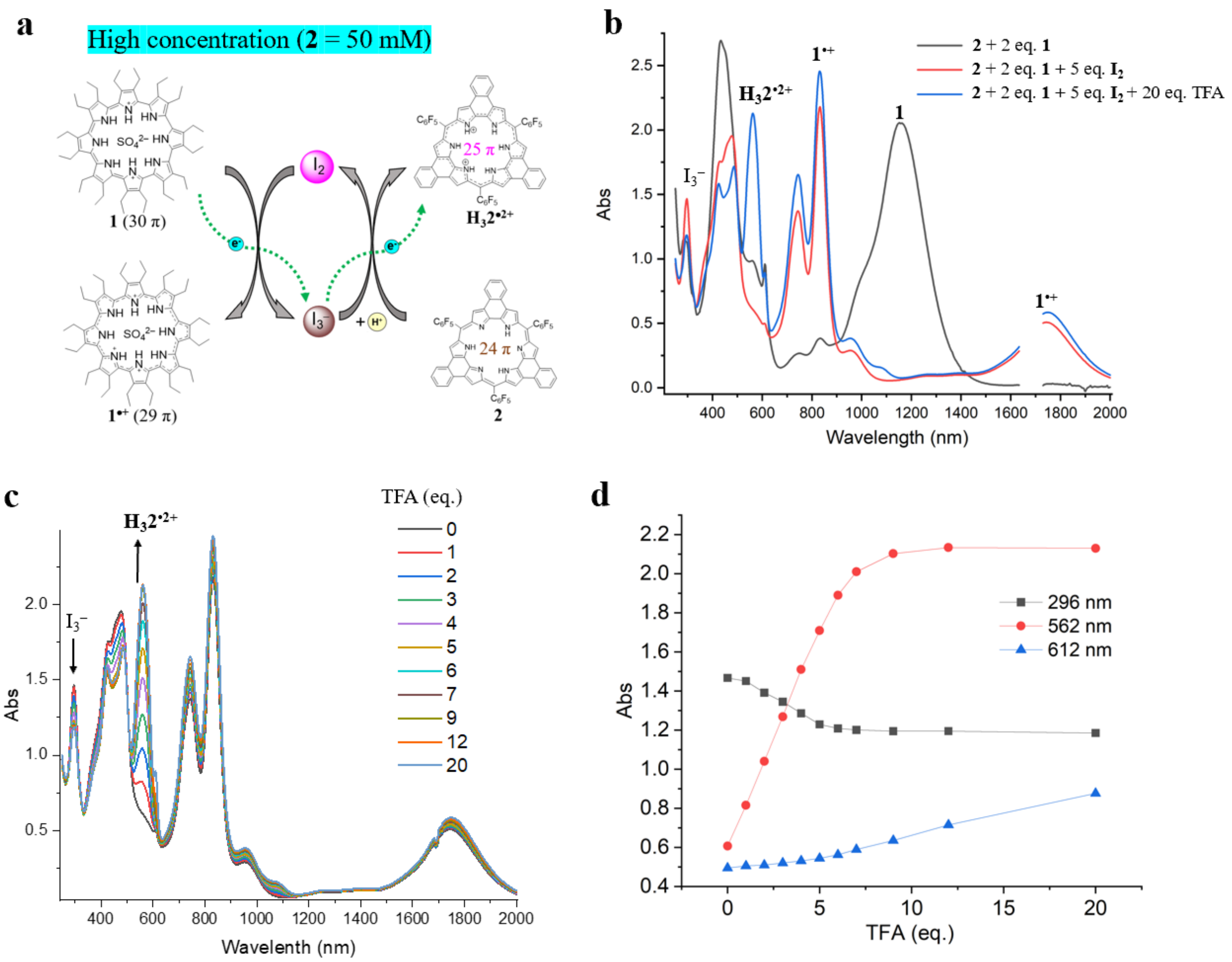

Fig. S28.

a, Schematic representation of electron transfer from **1** to I_2 and further to give $\mathbf{H_32^{\bullet 2+}}$. **b**, UV-Vis-NIR spectra of a mixed solution of **2** (50 μM) and 2.0 molar eq **2**, recorded upon the stepwise addition of 5.0 molar eq of I_2 and 20 eq of TFA. **c**, UV-Vis-NIR spectral titrations of TFA into a mixture of **2** (50 μM), 2.0 molar eq **1**, and 5.0 molar eq of I_2 (CH_2Cl_2, 0.2 cm optical path). **d**, Absorption changes at 296 nm, 562 nm, and 612 nm seen up titration with TFA. Note the lines have been added to aid in visualization.

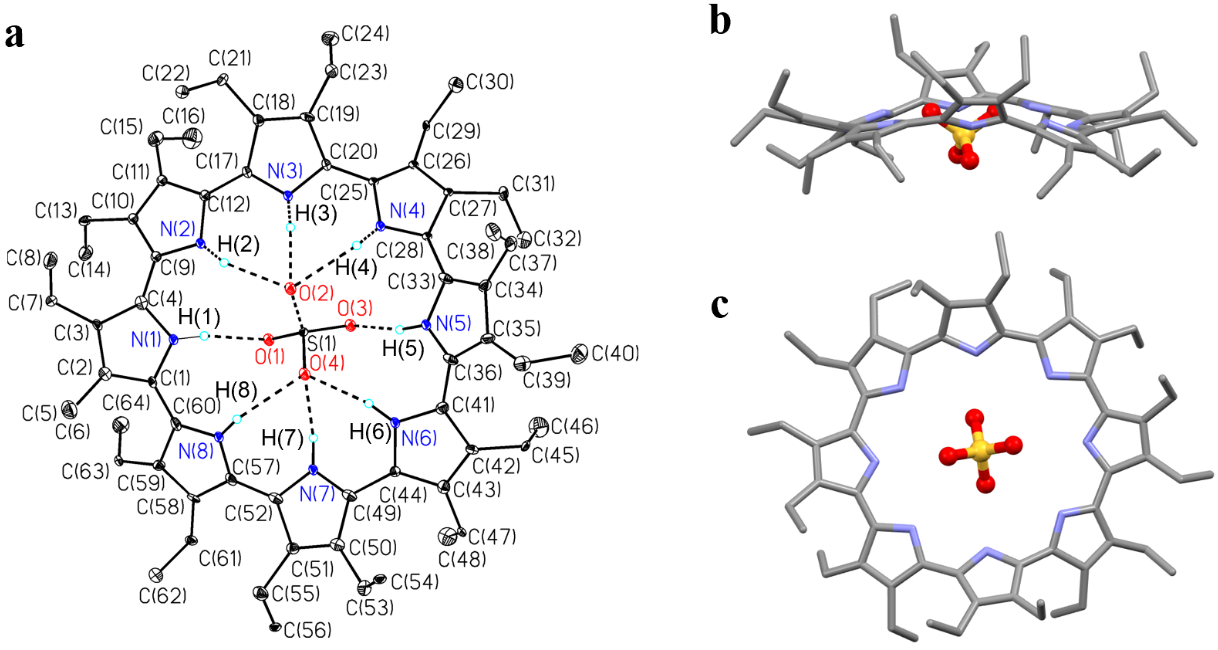

Fig. S29.

a, Top view in ellipsoid form of the structure of $\mathbf{1}^{\bullet+}$ as deduced from a single crystal X-ray diffraction analysis of $[(\mathbf{1}^{\bullet+})_2{\bullet}(I_{25})^{2-}{\bullet}I_2]$. **b** and **c**, Side and top views of the structure shown in ball and stick form. Displacement ellipsoids are scaled to the 25% probability level. All other molecules and atoms have been omitted for clarity. Possible hydrogen bond interactions were inferred from the following selected distances and selected interatomic angles: H(1)---O(1) 1.998 [Å], N(1)-H(1)…O(1) 165.54º; H(2)---O(2) 2.047 [Å], N(2)-H(2)…O(2) 164.17º; H(3)---O(2) 1.998 [Å], N(3)-H(3)…O(2) 148.86º; H(4)---O(2) 2.322 [Å], N(4)-H(4)…O(2) 149.79º; H(5)---O(3) 2.009 [Å], N(5)-H(5)…O(3) 168.03º; H(6)---O(4) 2.134 [Å], N(6)-H(6)…O(4) 163.30º; H(7)---O(4) 1.946 [Å], N(7)-H(7)…O(4) 152.31º; H(8)---O(4) 2.277 [Å], N(8)-H(8)…O(4) 153.02º.

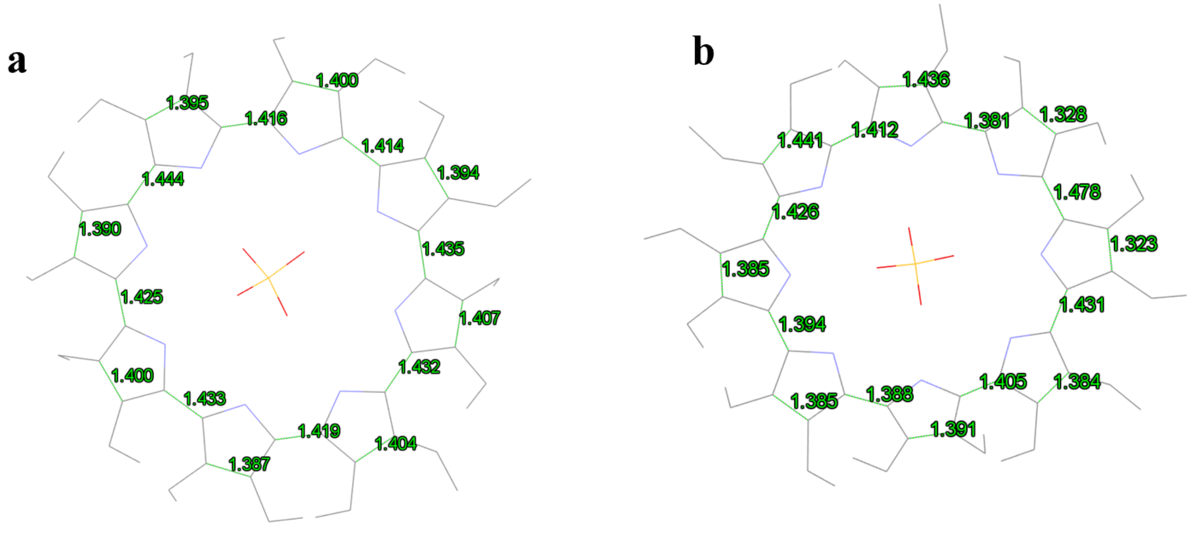

1 (30 π) **1**$^{\bullet+}$ (29 π)

Fig. S30.

Top view in wireframe form and bond lengths of the structures of **1** (a) and **1**$^{\bullet+}$ (b) as deduced from a single crystal X-ray diffraction analyses of [**1**] and [(**1**$^{\bullet+}$)$_2$•(I_{25})$^{2-}$•I_2].

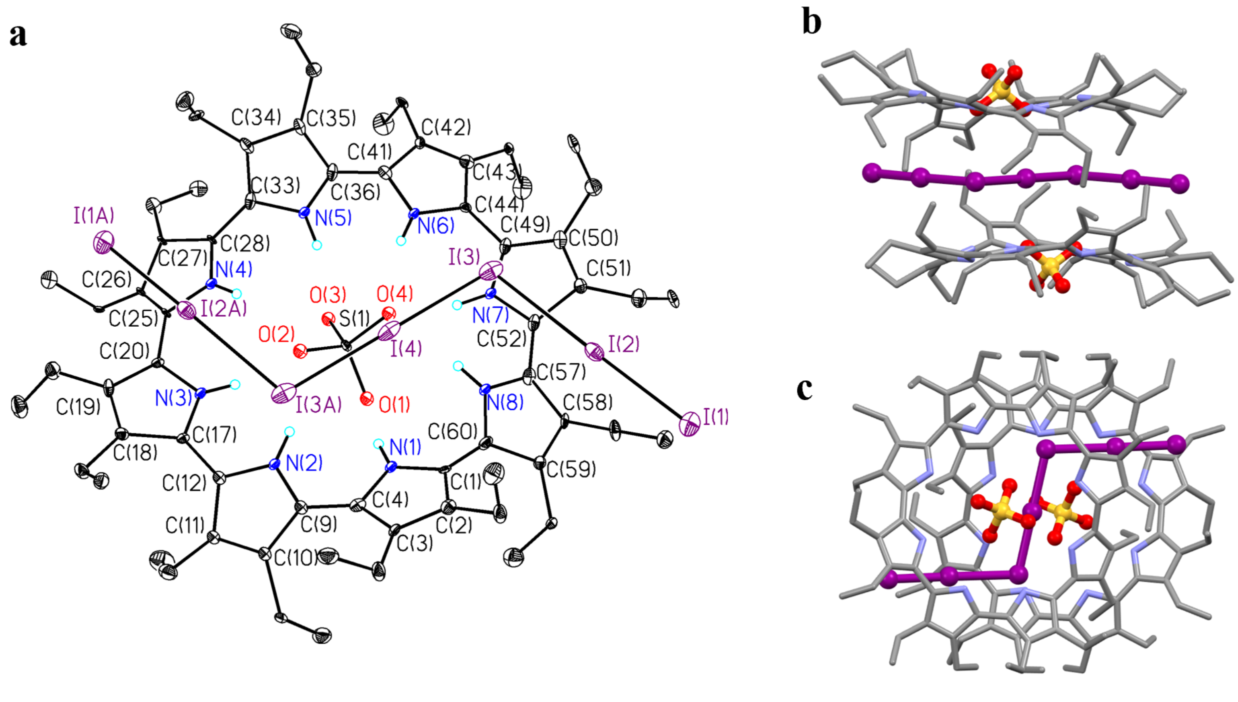

Fig. S31.

a, Top view in ellipsoid form showing the interactions between I_7^- and **1**$^{\bullet+}$ as observed in the single crystal structure of [(**1**$^{\bullet+}$)$_2$•(I_{25})$^{2-}$•I_2]. **b** and **c**, Side and top views in ball and stick form of the self-assembled dimer (**1**$^{\bullet+}$)$_2$⊇(I_7^-). Displacement ellipsoids are scaled to the 25% probability level. All

other molecules and atoms have been omitted for clarity. Possible halogen-π and the anion-π interactions are inferred from the following selected distances [Å]: I2---N3 3.83(4), I3---N2 3.51(3), I3---C9 3.73(4), I3---N7 3.62(3), I3---C52 3.66(4), I2---N8 3.98(3), I2---C57 3.96(4), I1---C25 3.96(4), I4---N1 3.94, I4---O2 3.90(0). Possible halogen-halogen interaction was inferred from the following selected distance: [Å] I2---I3 3.15(6).

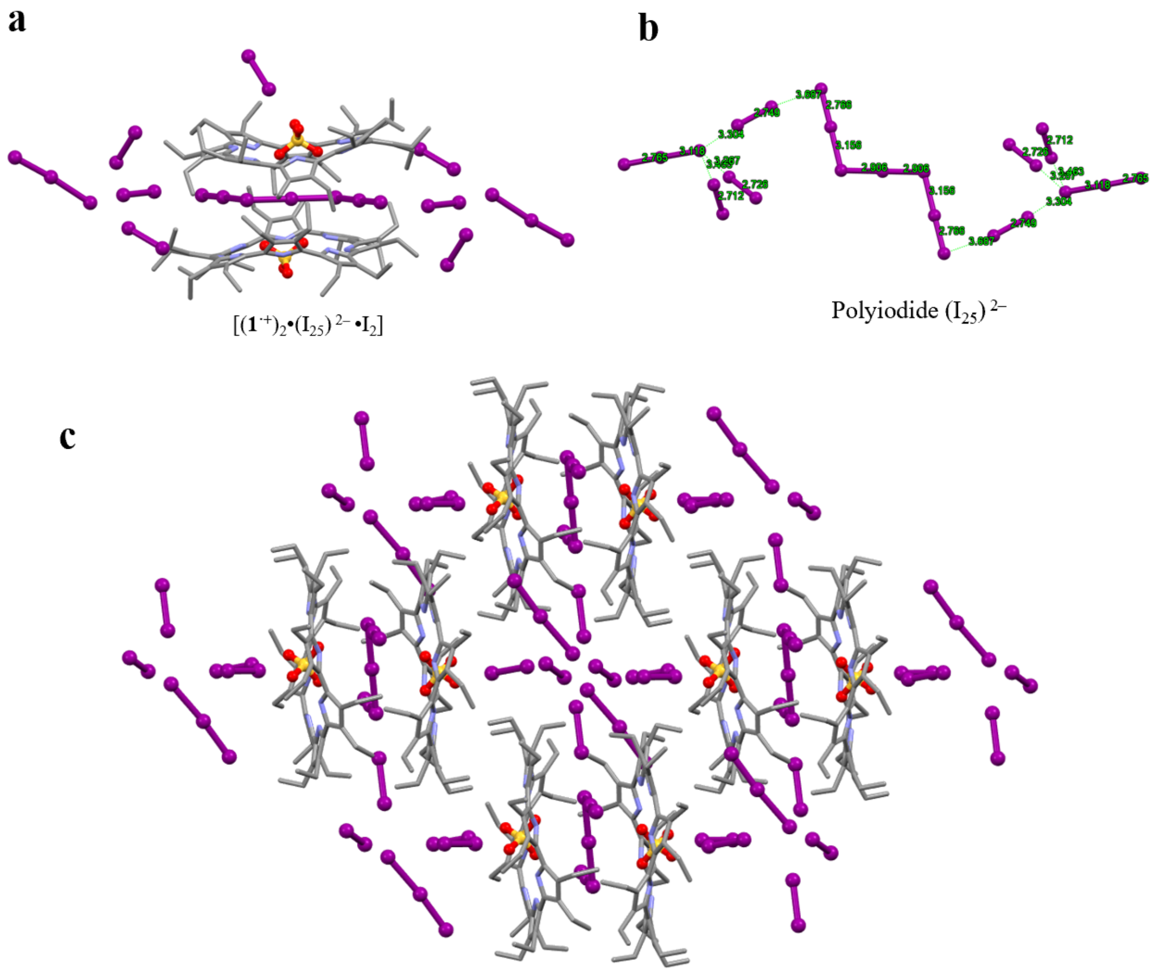

Fig. S32.

Periodic repeat unit, polyiodide $(I_{25})^{2-}$, and 2D packing structure seen in the single crystals of $[(\mathbf{1}^{\bullet+})_2\bullet(I_{25})^{2-}\bullet I_2]$.

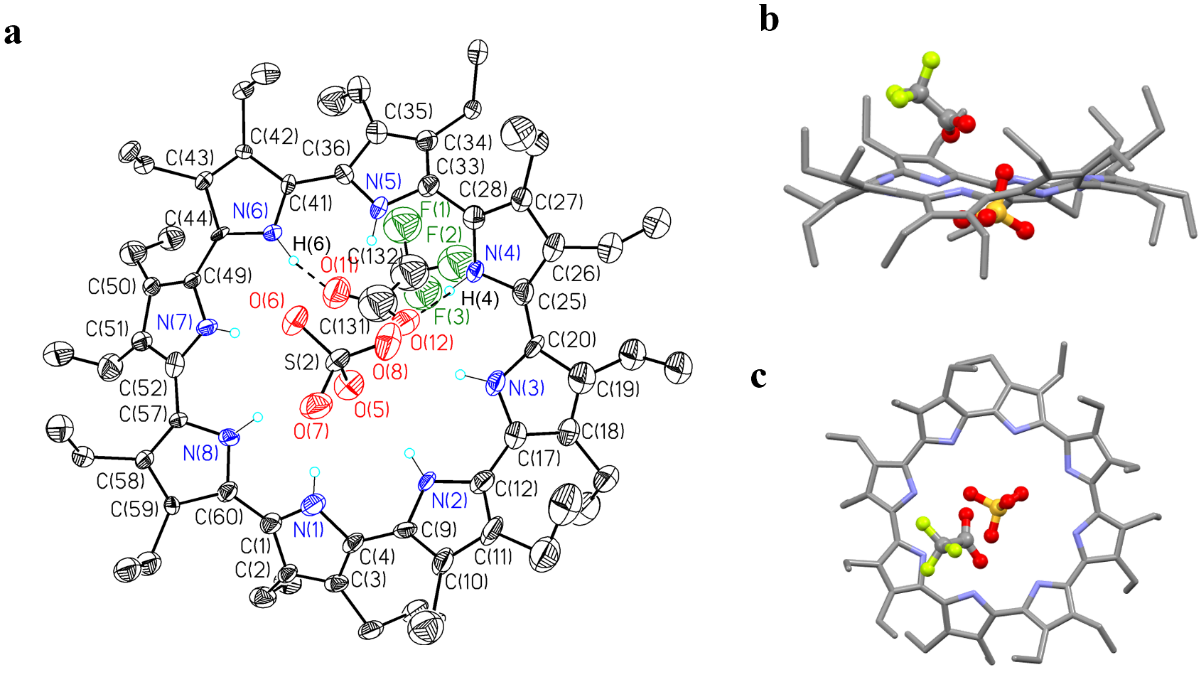

Fig. S33.

a, Top view in ellipsoid form showing the interactions between $CF_3CO_2^-$ and $\mathbf{1}^{\bullet+}$ as inferred from a single crystal structural analysis of [$\mathbf{1}^{\bullet+}$•$CF_3CO_2^-$]. **b** and **c**, Structure shown as side and top views in ball and stick form. Displacement ellipsoids are scaled to the 25% probability level. All other molecules and atoms have been omitted for clarity. Possible hydrogen bond interactions were inferred from the following selected distances and selected interatomic angles: H(4)---O(12) 2.04(2) [Å], N(4)-H(4)…O(12) 162.08º; H(6)---O(11) 2.23(0) [Å], N(6)-H(6)…O(11) 133.02º. Possible CF-π interactions were inferred from the following selected interatomic distances [Å]:F1---C35 3.54(3), F2---C33 3.49(3), F1---C36 3.66(3), F1---C34 3.59(2), F1---N5 3.62(2), F2---N5 3.77(3), F2---C28 3.52(3), F2---N4 3.17(3).

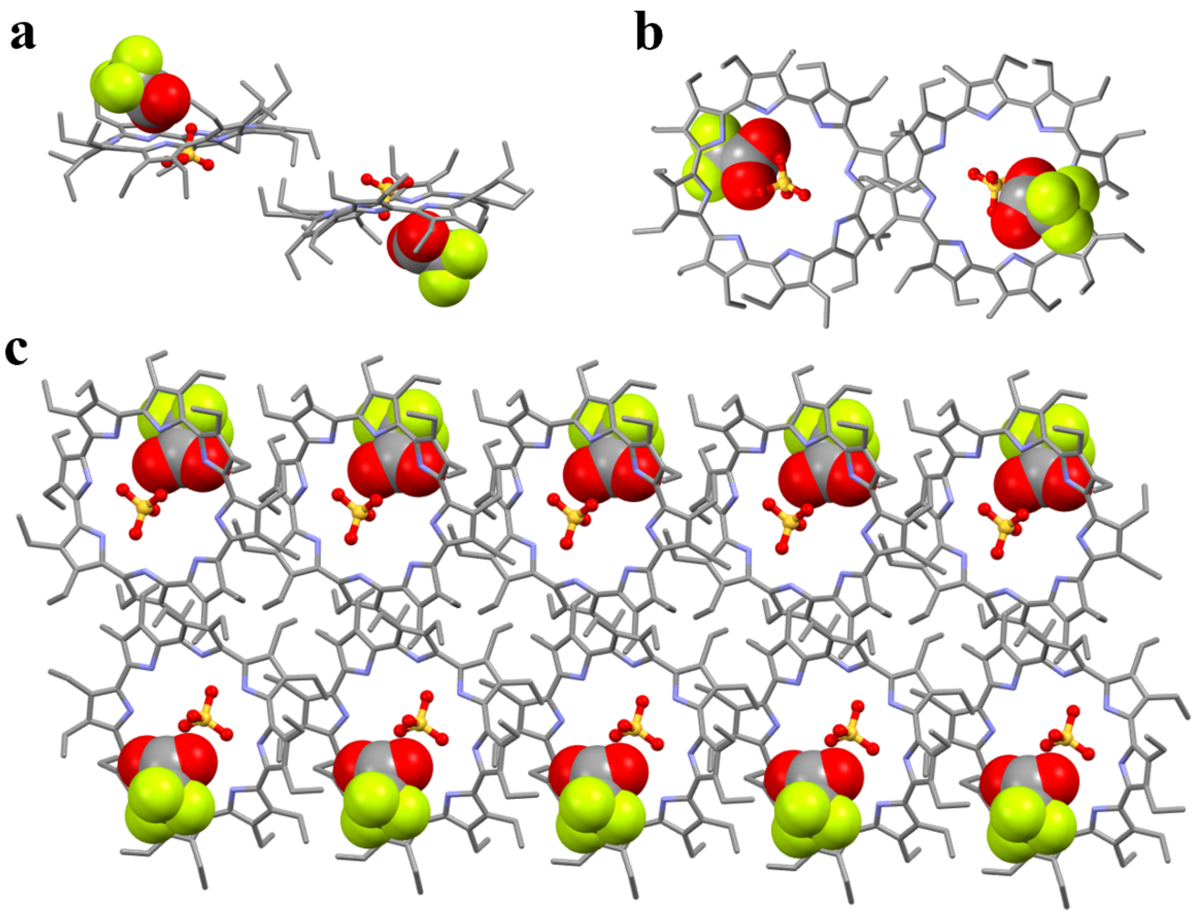

Fig. S34.

Side (**a**) and top (**b**) views of periodic repeat unit and 2D packing structure seen in single crystals of [$\mathbf{1}^{\bullet+}$•$CF_3CO_2^-$].

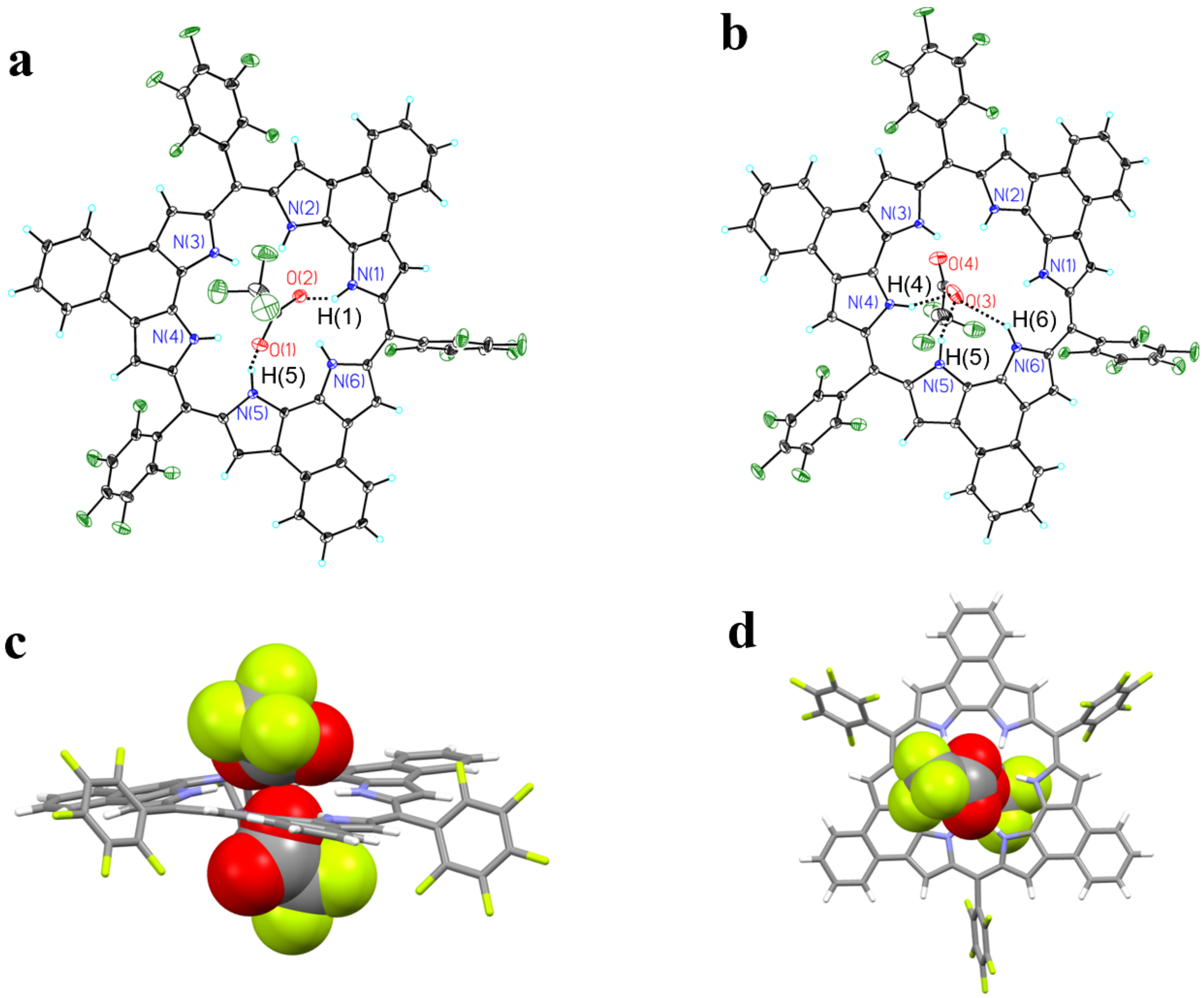

Fig. S35.

a and **b**, Top views in ellipsoid form showing the binding interactions between $CF_3CO_2^-$ and O(1), O(3) and $\mathbf{H_32}^{\bullet 2+}$ as observed in the single crystal structure of [$\mathbf{H_32}^{\bullet 2+}$•$(CF_3CO_2^-)_2$•$CHCl_3$]. **c** and **d**, Top and side views shown in space filling and stick form. Displacement ellipsoids are scaled to the 25% probability level. All other molecules and atoms have been omitted for clarity. Possible hydrogen bond interactions were inferred from the following selected distances and selected interatomic angles: H(1)---O(2) 2.13(5) [Å], N(1)-H(1)…O(2) 136.3°; H(5)---O(1) 2.20(7) [Å], N(5)-H(5)…O(1) 125.5°; H(1)---O(3) 2.08(3) [Å], N(1)-H(1)…O(3) 131.6°; H(5)---O(3) 2.34(6) [Å], N(5)-H(5)…O(3) 136.6°; H(6)---O(3) 2.36(5) [Å], N(6)-H(6)…O(3) 140.5°. Possible CF-π interactions are inferred from the following selected interatomic distances [Å]: F18---C25 3.10(9), F18---C26 3.42(6), N3---F18 3.25(9), F17---N3 3.39(9), F17---C22 3.43(4), F20---N5 3.18(1), F20---C43 3.58(1), F20---C46 3.61(1), F19---N6 3.08(1), F19---C47 3.57(1), F19---C50 3.58(1).

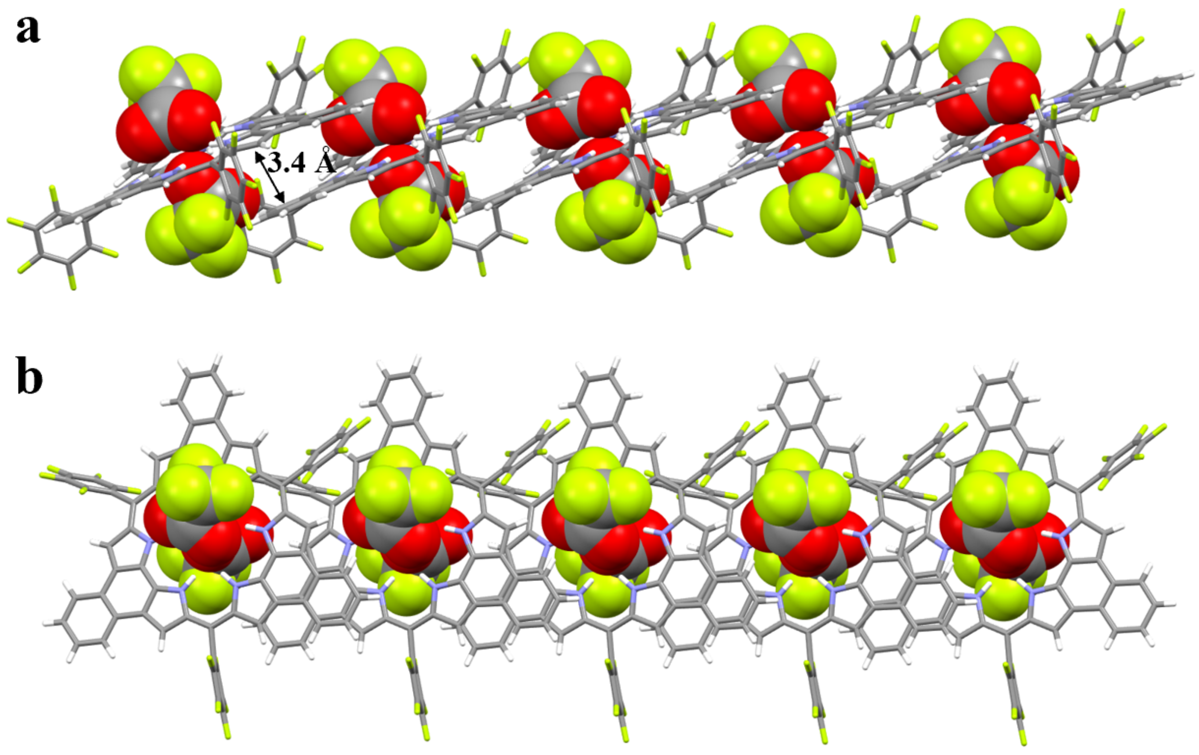

Fig. S36.

Side (**a**) and top (**b**) views of the 1D packing structure (**$H_3$2$^{\bullet 2+}$**•$(CF_3CO_2^-)_2$)$_n$ seen in single crystals of [**$H_3$2$^{\bullet 2+}$**•$(CF_3CO_2^-)_2$•$CHCl_3$]. Possible π-π donor-acceptor interactions as inferred from the closest distance between two macrocycles around 3.4 Å.

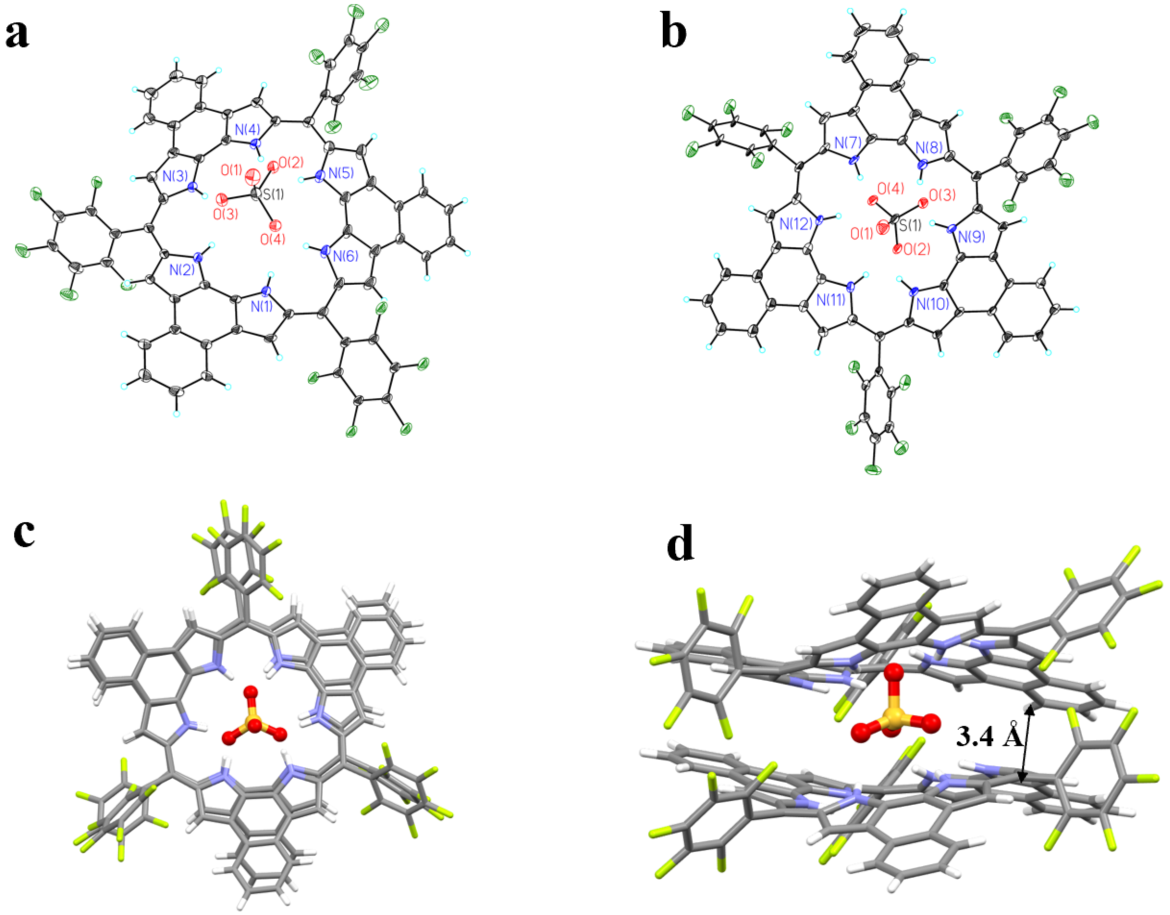

Fig. S37.

a and **b**, Top views in ellipsoid form showing the binding interactions between SO_4^{2-} and $\mathbf{H_32^{\bullet 2+}}$ with N(1), N(7) observed in the single crystal structure of $[(\mathbf{H_32^{\bullet 2+}})_2 \supset (SO_4^{2-}) \bullet 2I_3^- \bullet 2.5I_2 \bullet 6.25H_2O]$. **c** and **d**, Structure shown as top and side views in ball and stick form for dimer $(\mathbf{H_32^{\bullet 2+}})_2 \supset (SO_4^{2-})$. Displacement ellipsoids are scaled to the 25% probability level. All other molecules and atoms have been omitted for clarity. Possible hydrogen bond interactions are inferred from the following selected distances and selected interatomic angles: O1---H12 2.04(8) [Å], N12-H12…O1 152.27º; O1---H11 2.19(9) [Å], N11-H11…O1 147.87º; O1---H10 2.50(8) [Å], N10-H10…O1 134.89º; O1---H9 2.59(9) [Å], N9-H9…O1 135.03º; O1---H8 2.59(1) [Å], N8-H8…O1 145.57º; O1---H7 2.43(2) [Å], N7-H7…O1 148.33º; O2---H4 1.90(5) [Å], N4-H4…O2 143.30º; O2 ---H5 2.00(7) [Å], N5-H5…O2 140.15º; O3---H8 1.94(6) [Å], N8-H8…O3 146.60º; O3---H3 1.92(7) [Å], N3-H3…O3 149.76º; O4---H6 2.13(9) [Å], N6-H6…O4 147.94º. Possible π-π donor-acceptor interactions as inferred from the closest distance between two macrocycles around 3.4 Å.

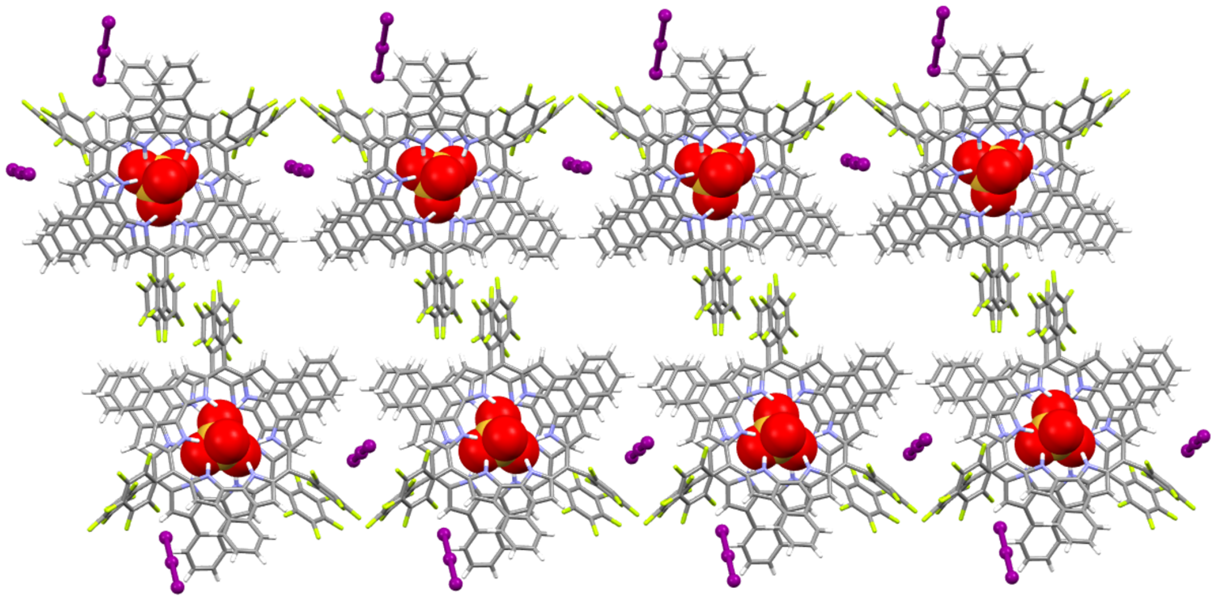

Fig. S38.

2D packing structure of dimer ($\mathbf{H_32}^{\bullet 2+}$)$_2$⊃($SO_4^{2-}$) and I_3^- as seen in single crystals of [($\mathbf{H_32}^{\bullet 2+}$)$_2$⊃($SO_4^{2-}$)•$2I_3^-$•$2.5I_2$•$6.25H_2O$].

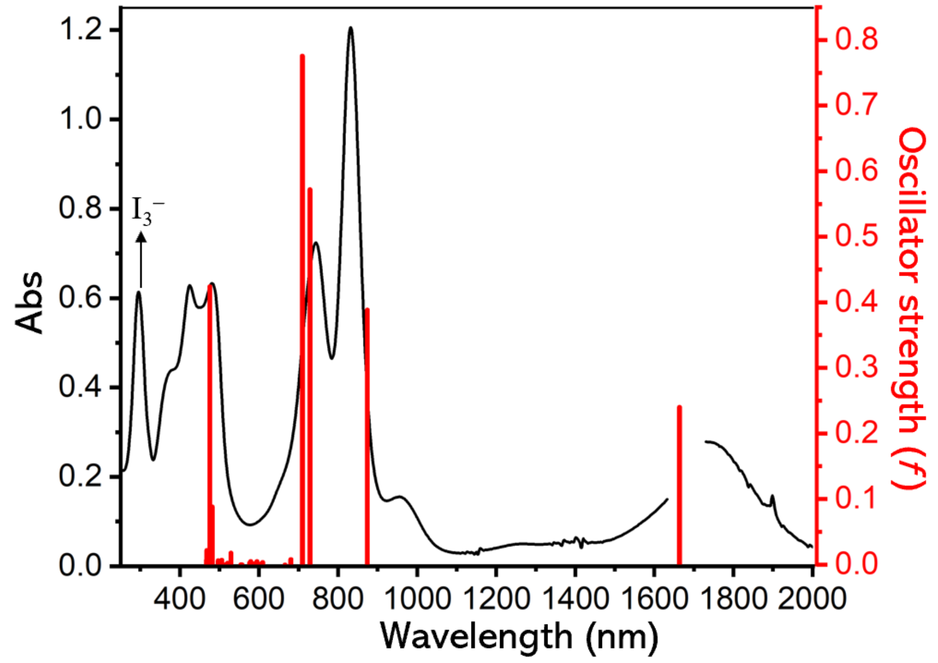

Fig. S39.

a, UV-Vis-NIR spectra (CH_2Cl_2) of $\mathbf{1}^{\bullet+}$•I_3^- (black line) prepared by mixed **1** (10 μM) in the presence of 5.0 molar eq of I_2 along with the theoretical vertical excitation energies for $\mathbf{1}^{\bullet+}$ obtained from TDDFT calculations carried out at the (U)B3LYP/6-311+G(d,p) level (red bars).

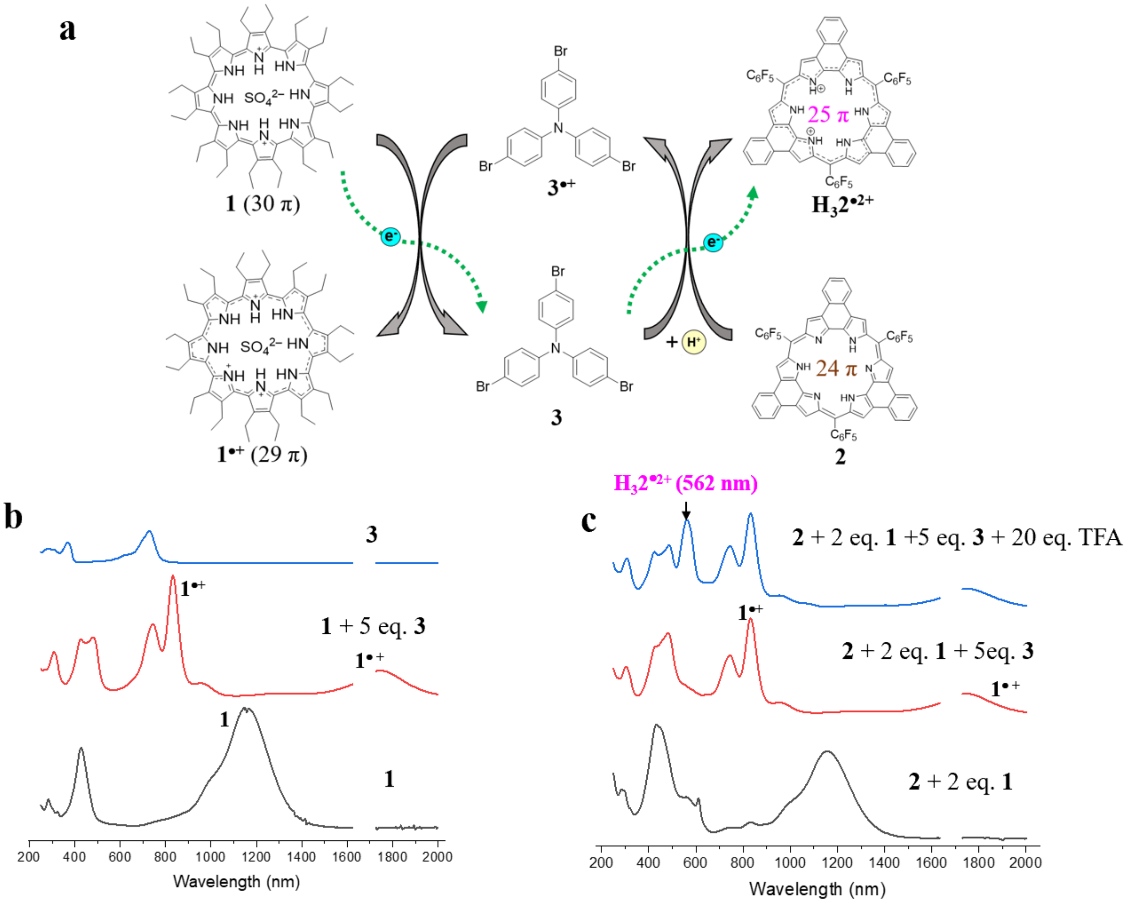

Fig.40.

a, Schematic representation of electron transfer from **1** to $\mathbf{3}^{\bullet+}$ (*tris*-(4-bromophenyl)ammoniumyl hexachloroantimonate, $[(p\text{-}BrC_6H_4)_3N^{\bullet}]^+[SbCl_6]^-$) and further to give $\mathbf{H_32}^{\bullet 2+}$. **b**, UV-Vis-NIR spectra of **1** (100 μM), a mixed solution of **1** (500 μM) and 5.0 molar eq **3**, and **3** (100 μM). **c**, UV-Vis-NIR spectra of a mixed solution of **1** (50 μM) and 2.0 molar eq **2**, recorded upon the stepwise addition of 5.0 molar eq of **3** and 20 eq of TFA (CH_2Cl_2, 0.2 cm optical path).

	$[(\mathbf{1}^{\bullet+})_2{\bullet}(I_{25})^{2-}{\bullet}I_2]$	$[\mathbf{1}^{\bullet+}{\bullet}CF_3CO_2^-]$
CCDC No.	2267375	2267378
Empirical formula	$C_{64}H_{88}I_{13.5}N_8O_4S$	$C_{66}H_{88}N_8O_6F_3S$
Formula weight	2778.63	1178.50
Temperature/K	173(2)	100(2)
Crystal system	triclinic	monoclinic
Space group	P-1	$P2_1$
a/Å	15.844(3)	10.8389(9)
b/Å	16.742(3)	52.875(3)
c/Å	18.517(4)	11.1333(10)
α/°	70.34(3)	90
β/°	69.76(3)	105.298(8)
γ/°	77.82(3)	90
Volume/Å^3	4314.6(19)	6154.5(9)
Z	2	4
ρ_{calc}g/cm^3	2.139	1.272
μ/mm^{-1}	38.605	1.018
F(000)	2583.0	2524.0
Crystal size/mm^3	0.05 × 0.01 × 0.01	0.05 × 0.01 × 0.01
Radiation	CuKα (λ = 1.54184)	CuKα (λ = 1.54184)
2Θ range for data collection/°	5.638 to 124.996	6.686 to 124.996
Index ranges	$-17 \le h \le 18$, $-19 \le k \le 19$, $-21 \le l \le 21$	$-12 \le h \le 12$, $-58 \le k \le 60$, $-10 \le l \le 12$
Reflections collected	39067	50383
Independent reflections	13699 [R_{int}= 0.0784, R_{sigma}= 0.0830]	16971 [R_{int} = 0.0760, R_{sigma} = 0.0744]
Data/restraints/parameters	13699/463/707	16971/1009/1447
Goodness-of-fit on F^2	1.064	1.019
Final R indexes [I>=2σ (I)]	R_1= 0.1233, wR_2= 0.3334	R_1 = 0.1391, wR_2 = 0.3019
Final R indexes [all data]	R_1= 0.1368, wR_2= 0.3417	R_1 = 0.1712, wR_2 = 0.3256
Largest diff. peak/hole / e Å^{-3}	4.22/-2.26	0.80/-0.46

Table S3.

X-ray crystallographic data for $[(\mathbf{1}^{\bullet+})_2{\bullet}(I_{25})^{2-}{\bullet}I_2]$ and $[\mathbf{1}^{\bullet+}{\bullet}CF_3CO_2^-]$.

	$[H_32^{\bullet 2+}\bullet(CF_3CO_2^-)_2\bullet CHCl_3]$	$[(H_32^{\bullet 2+})_2\supset(SO_4^{2-})\bullet 2I_3^-\bullet 2.5I_2\bullet 6.25H_2O]$
CCDC No.	2267376	2267377
Empirical formula	$C_{69}H_{26}N_6O_4F_{21}Cl_6$	$C_{126}H_{60.5}F_{30}I_{11}N_{12}O_{10.25}S$
Formula weight	1614.66	3904.32
Temperature/K	201(2)	173(2)
Crystal system	monoclinic	monoclinic
Space group	$P2_1/c$	$P2_1/c$
a/Å	10.1442(3)	14.710(3)
b/Å	14.4587(5)	67.008(13)
c/Å	43.3261(10)	15.490(3)
α/°	90	90
β/°	94.316(2)	103.22(3)
γ/°	90	90
Volume/Å^3	6336.7(3)	14863(5)
Z	4	4
ρ_{calc}g/cm^3	1.692	1.745
μ/mm^{-1}	3.558	18.951
F(000)	3220.0	7406.0
Crystal size/mm^3	0.05 × 0.01 × 0.01	0.05 × 0.03 × 0.02
Radiation	CuKα (λ = 1.54184)	CuKα (λ = 1.54184)
2Θ range for data collection/°	6.446 to 125	5.276 to 125
Index ranges	$-11 \le h \le 11$, $-16 \le k \le 16$, $-34 \le l \le 49$	$-16 \le h \le 16$, $-77 \le k \le 45$, $-17 \le l \le 17$
Reflections collected	55627	54161
Independent reflections	10107 [R_{int} = 0.0528, R_{sigma} = 0.0392]	23281 [R_{int} = 0.0494, R_{sigma} = 0.0634]
Data/restraints/parameters	10107/156/970	23281/490/1519
Goodness-of-fit on F^2	0.990	1.005
Final R indexes [I>=2σ (I)]	R_1 = 0.1002, wR_2 = 0.2394	R_1 = 0.1033, wR_2 = 0.2165
Final R indexes [all data]	R_1 = 0.1148, wR_2 = 0.2508	R_1 = 0.1151, wR_2 = 0.2241
Largest diff. peak/hole / e Å^{-3}	1.61/-1.27	2.93/-2.37

Table S4.

X-ray crystallographic data for $[H_32^{\bullet 2+}\bullet(CF_3CO_2^-)_2\bullet CHCl_3]$ and $[(H_32^{\bullet 2+})_2\supset(SO_4^{2-})\bullet 2I_3^-\bullet 2.5I_2\bullet 6.25H_2O]$.

Compound	Solvent	E_{ox} (V, Fc/Fc$^+$)	E_{red} (V, Fc/Fc$^+$)
I_2	Dichloromethane (0.1 M TBA•PF_6)	---	+0.15, -0.57
**1**	Dichloromethane (0.1 M TBA•PF_6)	-0.12, +0.30, +0.88	-0.71
**2**	Dichloromethane (0.1 M TBA•PF_6)	+0.43, +0.72	-0.60, -0.96
I_2	Dichloromethane (0.1 M TBA•PF_6 + 0.02 M TFA)	---	+0.16, -0.41
I_3^-	Dichloromethane (0.1 M TBA•PF_6 + 0.02 M TFA)	+0.16	-0.41
**1**	Dichloromethane (0.1 M TBA•PF_6 + 0.2 M TFA)	-0.10, +0.20, +0.82	-0.56
$\mathbf{H_3 2^{3+}}$	Dichloromethane (0.1 M TBA•PF_6 + 0.02 M TFA)	+0.76	+0.41, +0.02
$\mathbf{H_3 2^{\bullet 2+}}$	Dichloromethane (0.1 M TBA•PF_6 + 0.02 M TFA)	+0.76, +0.41	+0.02
$\mathbf{H_3 2^{+}}$	Dichloromethane (0.1 M TBA•PF_6 + 0.02 M TFA)	+0.76, +0.41, +0.02	--

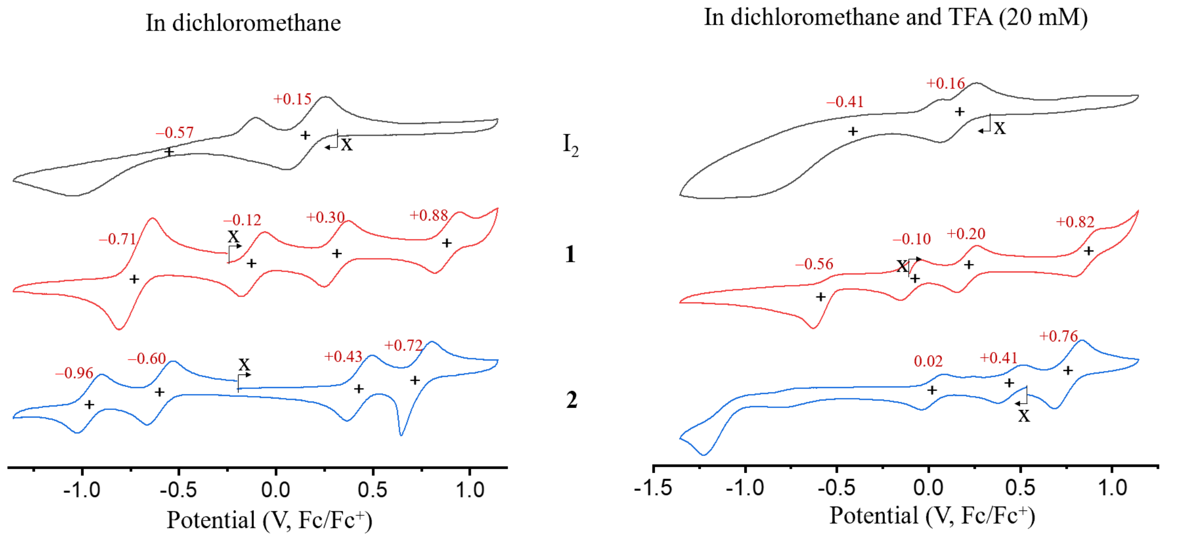

Table S5.

Summary of electrochemical data for I_2, **1**, **2**, and their ionic species. Voltammetric curves were recorded on a glassy carbon working electrode (∅ = 3mm, 100 mV/s, E in volts *vs* E_{ref}[Ag^+/Ag], and calibrated to Fc/Fc$^+$ ($E_{1/2}$ = +0.36 V)).

Process	Reactions	ΔE (kcal·M^{-1})
ETC Step one ($\mathbf{1} \rightarrow I_3^-$)	$[\mathbf{1}] + \frac{3}{2}[I_2] \rightleftharpoons [\mathbf{1}^{\bullet+}] + [I_3^-]$	-8.71
	$[\mathbf{1_a}^{2+}] + \frac{3}{2}[I_2] \rightleftharpoons [\mathbf{1_a}^{\bullet 3+}] + [I_3^-]$	5.87
ETC Step two ($I_3^- \rightarrow \mathbf{H_32}^{\bullet 2+}$)	$[\mathbf{2}] + [I_3^-] + 3[CF_3CO_2H] \rightleftharpoons [\mathbf{H_32}^{\bullet 2+}] + 3[CF_3CO_2^-] + \frac{3}{2}[I_2]$	-39.8
ETC Step three ($\mathbf{H_32}^{\bullet 2+} \rightarrow \mathbf{H_32}^{+}$)	$2[\mathbf{H_32}^{\cdot 2+}] + 2[CF_3CO_2^-] \rightleftharpoons [\mathbf{H_32}^{+}] + [\mathbf{H2}^{+}] + 2[CF_3CO_2H]$	-22.7
	$2[\mathbf{H_32}^{\bullet 2+}] + [CF_3CO_2^-] \rightleftharpoons [\mathbf{H_32}^{+}] + [\mathbf{H_22}^{2+}] + [CF_3CO_2H]$	-7.82
	$2[\mathbf{H_32}^{\bullet 2+}] \rightleftharpoons [\mathbf{H_32}^{+}] + [\mathbf{H_32}^{3+}]$	20.8
	$2[\mathbf{H_32}^{\bullet 2+}] + 3[CF_3CO_2^-] \rightleftharpoons [\mathbf{H_32}^{+}] + [\mathbf{2}] + 3[CF_3CO_2H]$	34.3
ET ($\mathbf{1} \rightarrow \mathbf{H_32}^{\bullet 2+}$ or $\mathbf{H_32}^{+}$)	$[\mathbf{1}] + [\mathbf{2}] + 3[CF_3CO_2H] \rightleftharpoons [\mathbf{1}^{\bullet+}] + [\mathbf{H_32}^{\bullet 2+}] + 3[CF_3CO_2^-]$	-48.5
	$[\mathbf{1_a}^{2+}] + [\mathbf{2}] + 3[CF_3CO_2H] \rightleftharpoons [\mathbf{1_a}^{\bullet 3+}] + [\mathbf{H_32}^{\bullet 2+}] + 3[CF_3CO_2^-]$	-28.1
	$[\mathbf{1}] + [\mathbf{H_32}^{\bullet 2+}] \rightleftharpoons [\mathbf{1}^{\bullet+}] + [\mathbf{H_32}^{+}]$	-14.2
	$[\mathbf{1_a}^{2+}] + [\mathbf{H_32}^{\bullet 2+}] \rightleftharpoons [\mathbf{1_a}^{\bullet 3+}] + [\mathbf{H_32}^{+}]$	6.22
complexes binding	$[\mathbf{1_a}^{2+}] + [SO_4^{2-}] \rightleftharpoons [\mathbf{1}]$	-71.2
	$[\mathbf{1_a}^{\bullet 3+}] + [SO_4^{2-}] \rightleftharpoons [\mathbf{1}^{\bullet+}]$	-91.2
	$[\mathbf{1}^{\bullet+}] + [I_3^-] \rightleftharpoons [\mathbf{1}^{\bullet+}\bullet I_3^-]$	-6.74
	$[\mathbf{1}^{\bullet+}] + [CF_3CO_2^-] \rightleftharpoons [\mathbf{1}^{\bullet+}\bullet CF_3CO_2^-]$	-0.31
	$[\mathbf{H_32}^{\bullet 2+}] + [CF_3CO_2^-] \rightleftharpoons [\mathbf{H_32}^{\bullet 2+}\bullet CF_3CO_2^-]$	-32.8
	$[\mathbf{H_32}^{\bullet 2+}\bullet CF_3CO_2^-] + [CF_3CO_2^-] \rightleftharpoons [\mathbf{H_32}^{\bullet 2+}\bullet (CF_3CO_2^-)_2]$	-19.4
	$[\mathbf{H_32}^{+}] + [CF_3CO_2^-] \rightleftharpoons [\mathbf{H_32}^{+}\bullet CF_3CO_2^-]$	-27.3
	$[\mathbf{H_32}^{3+}] + [CF_3CO_2^-] \rightleftharpoons [\mathbf{H_32}^{3+}\bullet CF_3CO_2^-]$	-40.8
	$[\mathbf{H_32}^{3+}\bullet CF_3CO_2^-] + [CF_3CO_2^-] \rightleftharpoons [\mathbf{H_32}^{3+}\bullet (CF_3CO_2^-)_2]$	-28.8

Note; $\mathbf{1_a}^{2+}$ and $\mathbf{1_a}^{\bullet 3+}$ are **1** and $\mathbf{1}^{\bullet+}$ without SO_4^{2-}.

Table S6.

Reaction energies (ΔE) as calculated in CH_2Cl_2 for the interconversions involved in the electron transport chain (ETC), electron transport (ET), or bound complexes comprised of **1**, I_2, and **2** based on the free energies of the species shown in Table S7.

I_2			
I	0	0	1.350278
I	0	0	-1.350278
Sum of electronic and thermal Free Energies= -595.658492			
I_3^-			
I	0	0	-2.966528
I	0	0	-0.000001
I	0	0	2.96653
Sum of electronic and thermal Free Energies=-893.667882			
CF_3CO_2H			
C	-0.592807	-0.000981	-0.001788
C	0.95057	0.156549	-0.004779
F	-1.186952	1.19102	-0.079719
F	-1.003079	-0.604848	1.129692
F	-1.003654	-0.74826	-1.041964
O	1.4924	1.224209	-0.002365
O	1.520708	-1.040836	-0.002368
H	2.491715	-0.941589	0.005185
Sum of electronic and thermal Free Energies=-526.972517			
$CF_3CO_2^-$			
C	0.520261	0.014168	-0.000016
C	-1.056011	0.012171	-0.000074
F	1.025665	-0.630731	-1.088494
F	1.025395	-0.629066	1.089573
F	1.081942	1.246369	-0.000927
O	-1.590833	1.131979	-0.000041
O	-1.531982	-1.136629	-0.000063

Sum of electronic and thermal Free Energies=-526.531890			
**1**			
O	0.104205	-1.273067	-1.398882
O	-1.226843	-0.125606	0.334173
O	-0.052279	1.204881	-1.379375
O	1.25668	0.029755	0.350664
S	0.019982	-0.041191	-0.541861
C	1.305667	-4.262197	0.49642
C	0.918525	-5.413602	1.221286
C	-0.48533	-5.493371	1.16232
C	-0.933771	-4.38523	0.401064
C	1.819776	-6.248578	2.086496
H	2.854068	-6.125093	1.765423
H	1.576014	-7.308891	1.965271
C	1.733072	-5.870835	3.57465
H	2.426746	-6.477956	4.16418
H	1.994111	-4.81872	3.719504
H	0.727594	-6.024377	3.971451
C	-1.340946	-6.433517	1.966031
H	-0.698253	-7.201814	2.401783
H	-2.052094	-6.96218	1.32871
C	-2.120889	-5.725866	3.086239
H	-2.691586	-6.451106	3.674041
H	-1.444429	-5.19567	3.761566
H	-2.823155	-4.997173	2.674601
C	-2.255616	-3.999031	0.025127
C	-3.322591	-4.779682	-0.474024
C	-4.431629	-3.930904	-0.638671
C	-4.029794	-2.62684	-0.24348

C	-3.20946	-6.188583	-0.982835
H	-2.330422	-6.664537	-0.551983
H	-4.075703	-6.777028	-0.664863
C	-3.077823	-6.248512	-2.514449
H	-2.977717	-7.285288	-2.849651
H	-3.946803	-5.813453	-3.012745
H	-2.192638	-5.69716	-2.843267
C	-5.728594	-4.349744	-1.26895
H	-6.548291	-4.249008	-0.554385
H	-5.668379	-5.416065	-1.493262
C	-6.069722	-3.588068	-2.557374
H	-7.006565	-3.958115	-2.984128
H	-6.18083	-2.517544	-2.374
H	-5.280792	-3.717849	-3.303107
C	-4.707158	-1.376757	-0.173198
C	-6.057724	-1.011741	0.076683
C	-6.121391	0.392821	0.087317
C	-4.809627	0.887468	-0.143995
C	-7.195115	-1.907668	0.476927
H	-7.483654	-2.559025	-0.350962
H	-8.067873	-1.28478	0.674701
C	-6.906548	-2.745947	1.731276
H	-7.774251	-3.3612	1.986801
H	-6.050715	-3.408672	1.591655
H	-6.688886	-2.095863	2.583597
C	-7.340345	1.186197	0.457373
H	-7.043888	2.202475	0.705915
H	-7.759059	0.766349	1.37854
C	-8.444653	1.206584	-0.610712
H	-9.274649	1.842531	-0.289352
H	-8.07572	1.587424	-1.56541

H	-8.838953	0.203858	-0.791715
C	-4.240882	2.195506	-0.143525
C	-4.735897	3.476093	-0.50556
C	-3.698624	4.403137	-0.299307
C	-2.579401	3.690782	0.18753
C	-6.03917	3.800861	-1.178618
H	-5.81709	4.348887	-2.100776
H	-6.522814	2.87837	-1.493386
C	-7.014812	4.645545	-0.343396
H	-7.951783	4.790927	-0.888821
H	-7.250277	4.170276	0.611062
H	-6.600435	5.631466	-0.122061
C	-3.67182	5.814949	-0.814508
H	-4.675635	6.241534	-0.780831
H	-3.046242	6.444585	-0.183324
C	-3.137467	5.881176	-2.255899
H	-3.143351	6.912222	-2.622345
H	-2.111049	5.508054	-2.304353
H	-3.746824	5.275629	-2.93186
C	-1.279735	4.176899	0.522258
C	-0.909899	5.33721	1.242648
C	0.497155	5.419493	1.202588
C	0.957993	4.302266	0.462968
C	-1.860364	6.172575	2.057655
H	-1.802857	7.221745	1.75245
H	-2.879178	5.844805	1.848242
C	-1.634119	6.070587	3.573925
H	-2.411417	6.625618	4.107388
H	-1.676815	5.027932	3.90132
H	-0.668438	6.47911	3.876251
C	1.377539	6.382423	1.952982

H	1.260652	6.200751	3.02664
H	2.420883	6.152	1.733412
C	1.10897	7.866957	1.673629
H	1.854653	8.487362	2.178966
H	1.15563	8.086993	0.60362
H	0.124942	8.17481	2.03204
C	2.285935	3.91901	0.1043
C	3.359006	4.703268	-0.380658
C	4.461868	3.851943	-0.563355
C	4.05496	2.543301	-0.187533
C	3.25995	6.125469	-0.853008
H	2.414214	6.615267	-0.376759
H	4.152569	6.683497	-0.554922
C	3.074369	6.223146	-2.376722
H	2.993636	7.26937	-2.68683
H	3.912179	5.773091	-2.914142
H	2.162142	5.705003	-2.68505
C	5.756457	4.274998	-1.196057
H	5.694021	5.342224	-1.41509
H	6.57961	4.171807	-0.485814
C	6.092258	3.521443	-2.490613
H	7.026312	3.895871	-2.919596
H	6.206162	2.449948	-2.315018
H	5.2995	3.654721	-3.231691
C	4.732264	1.293323	-0.129314
C	6.084098	0.928887	0.120071
C	6.150619	-0.474805	0.121456
C	4.839753	-0.971074	-0.11611
C	7.219724	1.823052	0.528964
H	8.093041	1.199593	0.721974
H	7.508155	2.48168	-0.293066

C	6.929735	2.649932	1.790538
H	7.797094	3.263176	2.051934
H	6.073738	3.313521	1.656399
H	6.712035	1.992082	2.636859
C	7.370918	-1.267176	0.489555
H	7.077075	-2.285546	0.731982
H	7.786362	-0.851284	1.413954
C	8.477624	-1.278109	-0.576259
H	9.308188	-1.914448	-0.257196
H	8.111722	-1.653328	-1.534385
H	8.870183	-0.273408	-0.749787
C	4.274027	-2.279702	-0.12745
C	4.773879	-3.559447	-0.488262
C	3.735088	-4.487745	-0.296431
C	2.610409	-3.777189	0.180566
C	6.085302	-3.881492	-1.146581
H	5.875511	-4.42525	-2.073928
H	6.573046	-2.957981	-1.451271
C	3.712097	-5.897176	-0.818788
H	4.70624	-6.340639	-0.740329
C	3.24133	-5.9518	-2.282815
H	3.248532	-6.982063	-2.65138
H	2.223335	-5.564087	-2.37477
H	3.887469	-5.3539	-2.930487
N	0.164627	-3.630793	0.065319
H	0.145943	-2.803772	-0.55632
N	-2.693761	-2.701918	0.104843
H	-2.131044	-1.918658	0.443114
N	-3.989701	-0.207745	-0.278699
H	-2.976657	-0.161759	-0.405621
N	-2.919582	2.362804	0.231086

H	-2.29861	1.614916	0.543404
N	-0.132151	3.541079	0.116227
H	-0.103802	2.716504	-0.508333
N	2.718771	2.618872	0.162424
H	2.152806	1.83134	0.485025
N	4.017783	0.123531	-0.244486
H	3.004486	0.077013	-0.371821
N	2.948497	-2.449756	0.231934
H	2.323382	-1.704429	0.542959
H	3.046585	-6.518051	-0.220175
C	7.049734	-4.730087	-0.302352
H	6.63356	-5.717969	-0.093645
H	7.994798	-4.870716	-0.834892
H	7.270406	-4.260631	0.658564
Sum of electronic and thermal Free Energies=-3629.049123			
**1$^{\bullet+}$**			
O	0.073063	-1.281763	-1.387905
O	-1.232509	-0.101934	0.34129
O	-0.032015	1.199404	-1.376342
O	1.253785	0.003974	0.355522
S	0.015134	-0.045444	-0.539487
C	1.214359	-4.225434	0.58902
C	0.81672	-5.32225	1.38299
C	-0.592085	-5.375613	1.341629
C	-1.019897	-4.308166	0.519042
C	1.711903	-6.136824	2.272321
H	2.742285	-6.063653	1.923422
H	1.431924	-7.192991	2.210912
C	1.666486	-5.680258	3.739926
H	2.351557	-6.279468	4.346782

H	1.963247	-4.631223	3.826487
H	0.664138	-5.782006	4.160576
C	-1.462587	-6.251468	2.198908
H	-0.838274	-7.020915	2.658043
H	-2.20231	-6.780852	1.595366
C	-2.198218	-5.466901	3.297607
H	-2.780884	-6.144918	3.927936
H	-1.493066	-4.92834	3.935747
H	-2.884911	-4.735735	2.86374
C	-2.335952	-3.919419	0.111933
C	-3.402942	-4.717252	-0.377729
C	-4.496208	-3.873618	-0.596022
C	-4.087863	-2.54566	-0.246081
C	-3.282968	-6.147863	-0.818484
H	-2.426378	-6.610059	-0.331189
H	-4.166872	-6.71215	-0.508274
C	-3.09307	-6.273588	-2.339805
H	-2.987073	-7.324462	-2.623847
H	-3.94074	-5.856792	-2.887658
H	-2.192487	-5.741802	-2.658824
C	-5.7729	-4.3176	-1.245748
H	-6.617107	-4.148533	-0.575995
H	-5.721515	-5.398303	-1.384463
C	-6.038078	-3.653751	-2.605142
H	-6.963134	-4.040386	-3.041432
H	-6.13301	-2.569806	-2.514445
H	-5.22071	-3.860657	-3.300621
C	-4.731461	-1.293432	-0.242483
C	-6.108401	-0.884332	-0.12589
C	-6.137358	0.504164	-0.13677
C	-4.780296	0.972866	-0.243996

C	-7.297739	-1.74204	0.189227
H	-7.489666	-2.444185	-0.622253
H	-8.177079	-1.100795	0.236405
C	-7.16849	-2.489638	1.525501
H	-8.069448	-3.078782	1.71643
H	-6.312226	-3.166384	1.534842
H	-7.045113	-1.781453	2.349525
C	-7.366511	1.328127	0.10484
H	-7.075391	2.333065	0.395307
H	-7.893603	0.910647	0.968363
C	-8.340489	1.378362	-1.083066
H	-9.178339	2.042691	-0.855177
H	-7.854552	1.743259	-1.990265
H	-8.744568	0.388191	-1.305132
C	-4.1911	2.255056	-0.205685
C	-4.639876	3.569503	-0.549044
C	-3.586009	4.452846	-0.291058
C	-2.500648	3.68851	0.208483
C	-5.909795	3.968952	-1.242749
H	-5.636214	4.555519	-2.125997
H	-6.416274	3.085322	-1.621607
C	-6.872868	4.809135	-0.387694
H	-7.788929	5.015186	-0.947896
H	-7.1494	4.29986	0.537817
H	-6.426263	5.76608	-0.110357
C	-3.494361	5.881488	-0.747096
H	-4.496203	6.299614	-0.848284
H	-2.978995	6.491053	-0.005356
C	-2.754295	5.9996	-2.090613
H	-2.710582	7.043805	-2.413111
H	-1.730514	5.626297	-2.005587

H	-3.259354	5.422626	-2.869904
C	-1.194664	4.133614	0.593983
C	-0.815913	5.24645	1.374828
C	0.595534	5.306904	1.347591
C	1.037278	4.224232	0.552768
C	-1.760199	6.064504	2.213007
H	-1.682562	7.122415	1.945564
H	-2.782787	5.76121	1.985248
C	-1.543591	5.904009	3.725364
H	-2.314624	6.452415	4.273975
H	-1.604372	4.851331	4.015607
H	-0.573128	6.286903	4.045114
C	1.487877	6.219093	2.144945
H	1.37541	5.975879	3.206795
H	2.528645	5.995416	1.906258
C	1.226026	7.718089	1.951658
H	1.98424	8.303212	2.479306
H	1.258708	7.996624	0.894965
H	0.250449	8.011727	2.34284
C	2.359028	3.835608	0.166446
C	3.436848	4.634373	-0.303979
C	4.523433	3.787416	-0.532521
C	4.106532	2.456349	-0.201202
C	3.33745	6.075175	-0.71338
H	2.513397	6.552932	-0.189783
H	4.245579	6.609035	-0.420877
C	3.105828	6.233598	-2.22559
H	3.023348	7.291864	-2.489154
H	3.92481	5.800727	-2.804369
H	2.181002	5.734059	-2.526855
C	5.800417	4.233428	-1.180459

H	5.750325	5.314963	-1.312216
H	6.645904	4.058958	-0.514067
C	6.060254	3.579203	-2.545462
H	6.984447	3.967921	-2.98168
H	6.153897	2.494437	-2.463631
H	5.240922	3.793024	-3.236618
C	4.749042	1.204633	-0.201051
C	6.127481	0.796758	-0.080117
C	6.158606	-0.590718	-0.093415
C	4.802061	-1.062047	-0.207256
C	7.315899	1.65338	0.241378
H	8.194965	1.011811	0.287376
H	7.50998	2.358678	-0.56665
C	7.184773	2.394223	1.581287
H	8.085362	2.982656	1.776026
H	6.328193	3.07054	1.59344
H	7.060949	1.681736	2.401507
C	7.387956	-1.413194	0.152128
H	7.097117	-2.418476	0.441323
H	7.911381	-0.995508	1.017722
C	8.366165	-1.461519	-1.032399
H	9.203052	-2.126367	-0.802576
H	7.883458	-1.824738	-1.941976
H	8.771196	-0.471096	-1.251444
C	4.217022	-2.344985	-0.175073
C	4.672903	-3.660151	-0.510598
C	3.618351	-4.544846	-0.263714
C	2.524957	-3.781867	0.221084
C	5.949013	-4.060074	-1.192491
H	5.682455	-4.649967	-2.07547
H	6.457229	-3.177253	-1.570679

C	3.531948	-5.970243	-0.730726
H	4.521569	-6.42822	-0.711562
C	2.952844	-6.059274	-2.153459
H	2.911995	-7.100479	-2.485857
H	1.939185	-5.65124	-2.185296
H	3.564835	-5.497833	-2.86413
N	0.086165	-3.597303	0.127934
H	0.079009	-2.798362	-0.530158
N	-2.75095	-2.624153	0.127415
H	-2.182099	-1.841959	0.462055
N	-3.979967	-0.142136	-0.276838
H	-2.959694	-0.120314	-0.364998
N	-2.868924	2.378952	0.205538
H	-2.271226	1.612672	0.523903
N	-0.06003	3.500194	0.15882
H	-0.043406	2.699687	-0.497424
N	2.766997	2.537729	0.164998
H	2.193134	1.753288	0.48574
N	3.999935	0.051867	-0.24103
H	2.979309	0.028745	-0.329794
N	2.889646	-2.47202	0.220078
H	2.28701	-1.707568	0.534147
H	2.903616	-6.55649	-0.060998
C	6.906986	-4.895921	-0.327403
H	6.459765	-5.852009	-0.048356
H	7.826387	-5.103882	-0.881358
H	7.177955	-4.382138	0.597252
Sum of electronic and thermal Free Energies=-3628.882863			
$\mathbf{1_a}^{\bullet 3+}\bullet CF_3CO_2^-$			
O	0.570385	-0.970815	0.977228

O	-0.661389	0.915294	0.903454
C	0.565611	-4.564669	0.03299
C	1.324862	-5.703052	-0.366063
C	2.6645	-5.317794	-0.431626
C	2.723086	-3.924823	-0.074185
C	0.7436	-6.993073	-0.868966
H	-0.239287	-7.153486	-0.42849
H	1.370382	-7.831064	-0.554153
C	0.594283	-7.000217	-2.400151
H	0.165289	-7.948518	-2.735066
H	-0.067735	-6.193377	-2.72662
H	1.557865	-6.867832	-2.897841
C	3.75008	-6.192496	-0.994844
H	3.28632	-7.121665	-1.326444
H	4.454852	-6.486415	-0.213706
C	4.513639	-5.574721	-2.174044
H	5.231227	-6.295965	-2.57339
H	3.826727	-5.298326	-2.978586
H	5.064894	-4.680107	-1.879319
C	3.775182	-2.995271	0.037236
C	5.151241	-3.126957	0.421101
C	5.745939	-1.871053	0.33045
C	4.736379	-0.943846	-0.083935
C	5.78348	-4.323745	1.062425
H	5.248962	-5.225507	0.784925
H	6.806341	-4.436006	0.693571
C	5.794525	-4.213956	2.597131
H	6.251547	-5.105361	3.034938
H	6.359076	-3.340869	2.93098
H	4.77594	-4.127042	2.984835
C	7.142835	-1.549784	0.769545

H	7.712022	-1.153744	-0.073442
H	7.635089	-2.481163	1.049925
C	7.216989	-0.570214	1.950166
H	8.258618	-0.4154	2.242924
H	6.789654	0.401756	1.69772
H	6.67577	-0.963851	2.814456
C	4.755514	0.458574	-0.276022
C	5.742793	1.362356	-0.752508
C	5.211036	2.661127	-0.649585
C	3.90124	2.548764	-0.12743
C	7.041031	1.035366	-1.432921
H	7.77919	0.707358	-0.695993
H	7.439957	1.957846	-1.855353
C	6.92246	-0.00191	-2.558197
H	7.894512	-0.148202	-3.036225
H	6.579824	-0.970848	-2.191416
H	6.217096	0.338143	-3.321539
C	5.86371	3.914401	-1.15597
H	5.127699	4.71883	-1.167233
H	6.153534	3.753791	-2.200338
C	7.104108	4.352292	-0.363343
H	7.509001	5.279023	-0.778461
H	6.867221	4.528118	0.687339
H	7.890299	3.595228	-0.403962
C	2.902468	3.535017	0.178914
C	2.965955	4.762187	0.871057
C	1.658317	5.296823	0.899027
C	0.820053	4.399291	0.205685
C	4.155765	5.330152	1.58945
H	3.876203	5.513498	2.631584
H	4.947709	4.581022	1.615141

C	4.694927	6.637613	0.989562
H	5.571738	6.97567	1.548166
H	4.987427	6.508139	-0.053834
H	3.94622	7.431786	1.029273
C	1.193361	6.431589	1.766591
H	1.926209	7.240475	1.746216
H	0.259058	6.842216	1.385438
C	0.964047	5.980513	3.220236
H	0.632982	6.824813	3.831297
H	0.195775	5.203951	3.266126
H	1.877907	5.578517	3.663643
C	-1.320285	5.630561	-0.51727
C	-2.66278	5.258412	-0.570407
C	-2.740024	3.875669	-0.184414
C	-0.725978	6.873857	-1.114703
H	-1.406066	7.715464	-0.978071
H	0.203895	7.134854	-0.611441
C	-0.432466	6.68517	-2.613888
H	-0.02483	7.60575	-3.040283
H	0.298676	5.886721	-2.766713
H	-1.338485	6.424565	-3.166744
C	-3.746868	6.145241	-1.112459
H	-3.387974	6.581154	-2.049486
H	-4.623503	5.553326	-1.375654
C	-4.13348	7.29299	-0.162541
H	-4.956894	7.87268	-0.587768
H	-4.445548	6.923362	0.816657
H	-3.292442	7.970275	-0.001947
C	-3.80176	2.956967	-0.080018
C	-5.200666	3.09594	0.225051
C	-5.781057	1.832872	0.161598

C	-4.746212	0.89551	-0.168024
C	-5.877939	4.310995	0.778758
H	-5.419499	5.211977	0.393378
H	-6.918519	4.326983	0.447983
C	-5.824063	4.344267	2.315652
H	-6.323685	5.241167	2.691263
H	-6.317893	3.470527	2.747643
H	-4.789117	4.357234	2.667609
C	-7.193914	1.517982	0.554134
H	-7.695065	2.453053	0.804003
H	-7.734905	1.111503	-0.302447
C	-7.31465	0.55779	1.746889
H	-8.366853	0.417409	2.007263
H	-6.88907	-0.422203	1.524665
H	-6.798759	0.960091	2.622573
C	-4.753838	-0.51068	-0.331448
C	-5.718896	-1.423873	-0.832738
C	-5.187589	-2.720694	-0.690472
C	-3.90012	-2.596561	-0.126462
C	-6.986795	-1.113362	-1.575169
H	-7.370281	-2.046572	-1.988229
H	-7.754911	-0.762957	-0.880619
C	-6.814525	-0.108932	-2.723268
H	-7.764284	0.027502	-3.246847
H	-6.483271	0.868669	-2.369135
H	-6.078679	-0.473309	-3.445483
C	-5.817205	-3.988623	-1.189146
H	-5.077521	-4.789305	-1.148921
H	-6.0685	-3.858723	-2.247703
C	-7.083967	-4.410958	-0.430465
H	-7.471073	-5.349531	-0.835924

H	-6.883988	-4.55922	0.631962
H	-7.870814	-3.658455	-0.51861
C	-2.91109	-3.581451	0.222469
C	-2.989359	-4.771535	0.968634
C	-1.683598	-5.315838	1.030002
C	-0.835962	-4.460306	0.300553
C	-4.193275	-5.295258	1.69707
H	-3.93036	-5.432964	2.750587
H	-4.978789	-4.538525	1.675362
C	-1.235776	-6.418229	1.94686
H	-1.97348	-7.223185	1.948392
C	-1.022253	-5.914283	3.385188
H	-0.703838	-6.736389	4.03224
H	-0.250464	-5.140274	3.412025
H	-1.93952	-5.491043	3.801052
N	1.414967	-3.505843	0.129209
H	1.141231	-2.584146	0.495945
N	3.556214	-1.647185	-0.184079
H	2.764841	-1.294143	-0.70414
N	3.626761	1.212471	0.021235
H	2.866839	0.870414	0.596149
N	1.582761	3.308886	-0.154054
H	1.295315	2.626238	-0.842923
N	-1.436269	3.447145	0.037105
H	-1.173966	2.535317	0.433433
N	-3.569448	1.602558	-0.232935
H	-2.726055	1.224966	-0.643241
N	-3.631431	-1.257482	0.015825
H	-2.912001	-0.911221	0.637741
N	-1.589441	-3.383234	-0.119177
H	-1.298383	-2.757994	-0.858136

H	-0.299971	-6.849013	1.591828
C	-4.737333	-6.623567	1.150172
H	-3.993975	-7.420043	1.22746
H	-5.619017	-6.932586	1.718077
H	-5.024737	-6.536538	0.101036
C	-0.050249	-0.053023	0.418858
C	-0.035542	-0.093941	-1.156238
F	-1.187687	0.361346	-1.704153
F	0.964433	0.68358	-1.649362
F	0.165904	-1.332031	-1.655277
C	-0.575427	4.496505	-0.079726
Sum of electronic and thermal Free Energies= -3455.885021			
**2**			
C	-3.553053	2.253274	-0.013996
C	-4.912689	1.889296	-0.02142
H	-5.737888	2.58316	-0.027271
C	-4.97866	0.488439	-0.019666
C	-3.639746	0.019403	-0.011973
C	-3.358086	-1.375444	-0.011532
C	-4.502987	-2.290233	-0.01782
C	-3.953644	-3.547843	-0.016566
H	-4.467819	-4.49627	-0.020491
C	-2.519706	-3.366701	-0.009527
C	-5.876834	-1.819172	-0.023582
C	-6.116695	-0.418624	-0.024981
C	-7.439565	0.048728	-0.030457
H	-7.626967	1.116058	-0.030756
C	-8.507161	-0.838206	-0.03518
H	-9.52294	-0.460234	-0.039324
C	-8.271829	-2.218216	-0.033911

H	-9.10479	-2.911697	-0.037198
C	-6.970695	-2.69939	-0.027942
H	-6.790932	-3.768192	-0.02644
C	-1.57766	-4.378559	-0.00932
C	-2.063947	-5.790213	-0.002911
C	-2.152869	-6.532388	-1.176257
C	-2.604844	-7.843718	-1.18171
C	-2.980819	-8.442816	0.012269
C	-2.90154	-7.728142	1.198894
C	-2.444776	-6.418379	1.178503
C	-0.170692	-4.20446	-0.00901
C	0.825356	-5.199007	0.005276
H	0.63996	-6.260859	0.014797
C	2.070695	-4.554626	0.005585
C	1.80675	-3.160788	-0.007435
C	2.87365	-2.219009	-0.004641
C	4.238527	-2.752905	0.013619
C	5.052492	-1.647985	0.017982
H	6.131118	-1.618834	0.031694
C	4.178765	-0.496801	0.001809
C	4.51796	-4.178289	0.0242
C	3.425127	-5.086333	0.019826
C	3.681116	-6.465691	0.0297
H	2.849841	-7.16112	0.026357
C	4.982643	-6.947269	0.044013
H	5.162845	-8.015977	0.051677
C	6.06026	-6.05361	0.048486
H	7.077158	-6.428381	0.059317
C	5.826972	-4.686123	0.038757
H	6.663707	-3.997144	0.042242
C	4.584607	0.824809	0.005762

C	6.05024	1.109258	0.020937
C	6.802725	1.106545	-1.148958
C	8.16558	1.366423	-1.144185
C	8.805483	1.64173	0.055746
C	8.08003	1.65557	1.238401
C	6.7186	1.391937	1.207885
C	3.731765	1.956949	-0.001692
C	4.095736	3.316925	-0.007395
H	5.108983	3.685544	-0.007387
C	2.914809	4.073224	-0.011164
C	1.839674	3.147499	-0.011008
C	0.490493	3.59989	-0.014876
C	0.269749	5.048949	-0.015048
C	-1.094169	5.200784	-0.014591
H	-1.660956	6.118921	-0.012916
C	-1.653152	3.868275	-0.01685
C	1.364181	6.003782	-0.015655
C	2.697251	5.512148	-0.014433
C	3.76292	6.424573	-0.01595
H	4.781006	6.053237	-0.015209
C	3.527991	7.792496	-0.018244
H	4.362932	8.483631	-0.01935
C	2.214937	8.278022	-0.019343
H	2.030315	9.345985	-0.021471
C	1.148161	7.391143	-0.018114
H	0.132248	7.768888	-0.019642
C	-2.999981	3.558463	-0.015929
C	-3.981922	4.683401	-0.019283
C	-4.371835	5.303073	-1.202007
C	-5.288118	6.344583	-1.215613
C	-5.839721	6.785846	-0.0216

C	-5.471616	6.185378	1.173786
C	-4.553532	5.145759	1.161513
F	-2.372816	-5.755515	2.343069
F	-3.262563	-8.306089	2.351516
F	-3.419678	-9.704668	0.018757
F	-2.683538	-8.532485	-2.327308
F	-1.80103	-5.978486	-2.34689
F	6.21197	0.849032	-2.325925
F	8.865197	1.355198	-2.285947
F	10.117419	1.893774	0.072524
F	8.697167	1.919339	2.396996
F	6.044636	1.406519	2.368429
F	-3.859686	4.89486	-2.373362
F	-5.644507	6.923789	-2.369025
F	-6.724509	7.786932	-0.022988
F	-6.002917	6.612645	2.326247
F	-4.215162	4.585505	2.332986
N	-2.80458	1.090321	-0.008656
H	-1.787303	1.098882	-0.004929
N	-2.190686	-2.006002	-0.007672
N	0.461624	-2.974197	-0.016375
H	-0.055175	-2.097811	-0.020415
N	2.836011	-0.89256	-0.011328
N	2.350457	1.889312	-0.00537
H	1.850404	1.003118	-0.006173
N	-0.638793	2.903431	-0.015722
Sum of electronic and thermal Free Energies= -4240.317667			
**H2^{+}**			
C	-3.980581	-1.479954	0.120367
C	-4.499792	-2.764497	-0.084746
H	-5.54671	-2.997879	-0.181364
C	-3.414879	-3.655106	-0.1782
C	-2.240442	-2.874362	0.000108
C	-0.991937	-3.493742	-0.059598
C	-0.898353	-4.885621	-0.362444
C	0.460904	-5.197028	-0.372817
H	0.906601	-6.150463	-0.605333
C	1.199818	-4.031365	-0.077105
C	-2.079542	-5.694434	-0.572725
C	-3.353736	-5.073883	-0.451698
C	-4.510014	-5.852018	-0.624961
H	-5.484409	-5.388782	-0.530518
C	-4.417261	-7.202297	-0.913585
H	-5.317809	-7.789613	-1.043664
C	-3.161802	-7.811172	-1.038243
H	-3.092976	-8.867888	-1.264793
C	-2.008679	-7.064647	-0.86903
H	-1.041789	-7.544302	-0.963035
C	2.588175	-3.88905	-0.010037
C	3.401741	-5.132018	-0.018225
C	4.265632	-5.42472	-1.075911
C	5.032467	-6.582106	-1.093792
C	4.947424	-7.476484	-0.030626
C	4.097117	-7.210206	1.038564
C	3.335803	-6.048997	1.033007
C	3.280763	-2.672131	0.070367
C	4.64522	-2.4631	0.368938
H	5.343906	-3.253996	0.58741
C	4.894938	-1.088412	0.374121
C	3.657468	-0.456144	0.080226
C	3.546885	0.953446	0.022758

C	4.768532	1.726915	0.20459
C	4.366981	3.036702	0.102611
H	4.970218	3.925248	0.196098
C	2.937367	3.013089	-0.102694
C	6.048313	1.097188	0.480869
C	6.109354	-0.318531	0.590118
C	7.334879	-0.934529	0.877925
H	7.386687	-2.013574	0.961768
C	8.483823	-0.179354	1.054055
H	9.424191	-0.668608	1.275526
C	8.42701	1.213643	0.942697
H	9.324646	1.804523	1.077535
C	7.223546	1.840725	0.660069
H	7.188017	2.920047	0.575402
C	2.114282	4.130732	-0.165626
C	2.762272	5.472513	-0.179031
C	3.436118	5.933842	-1.30901
C	4.04849	7.180559	-1.335567
C	3.995777	7.993372	-0.207878
C	3.33249	7.556837	0.934694
C	2.724255	6.308388	0.936239
C	0.700832	4.115758	-0.181516
C	-0.172354	5.220213	-0.222592
H	0.141432	6.247952	-0.294311
C	-1.481364	4.737903	-0.141239
C	-1.38784	3.322366	-0.064616
C	-2.568495	2.524904	0.033344
C	-3.856358	3.24598	0.078326
C	-4.804165	2.269643	0.175596
H	-5.873594	2.387625	0.237803
C	-4.102958	1.002778	0.165013

C	-3.949114	4.692016	0.007412
C	-2.753972	5.443926	-0.111515
C	-2.828786	6.839958	-0.188949
H	-1.920202	7.422587	-0.279934
C	-4.055301	7.487736	-0.150142
H	-4.096833	8.568266	-0.211303
C	-5.235166	6.747275	-0.032002
H	-6.192511	7.252649	-0.001734
C	-5.179339	5.364382	0.045035
H	-6.096539	4.795013	0.134436
C	-4.692227	-0.237713	0.178936
C	-6.176972	-0.337476	0.207458
C	-6.954601	0.004331	-0.898401
C	-8.33997	-0.099653	-0.877637
C	-8.975609	-0.558304	0.27144
C	-8.224696	-0.911323	1.388492
C	-6.84143	-0.800249	1.342954
F	2.543154	-5.804354	2.082322
F	4.024582	-8.061115	2.058355
F	5.676709	-8.58366	-0.03681
F	5.837325	-6.843876	-2.119716
F	4.34759	-4.592035	-2.118708
F	3.490145	5.174539	-2.408492
F	4.678893	7.601555	-2.430066
F	4.579467	9.186011	-0.221213
F	3.290221	8.331942	2.016701
F	2.106584	5.901821	2.051653
F	-6.36701	0.430327	-2.022367
F	-9.059081	0.229518	-1.949105
F	-10.299286	-0.660151	0.302347
F	-8.833995	-1.345846	2.489825

F	-6.137699	-1.136241	2.430724
N	-2.608293	-1.56661	0.18786
H	-2.049279	-0.713664	0.205871
N	2.697541	-1.407597	-0.083371
H	1.838428	-1.229695	-0.581713
N	2.460611	1.703738	-0.148443
N	-0.074746	2.972154	-0.092557
H	0.345943	2.048219	-0.066743
N	-2.707949	1.211972	0.08813
H	0.466909	-2.18728	0.677677
N	0.274497	-2.982409	0.085645
Sum of electronic and thermal Free Energies=-4240.746508			
**$H_2 2^{2+}$**			
C	-4.119083	1.131532	-0.085542
C	-4.688924	2.288887	0.436239
H	-5.703086	2.362382	0.793262
C	-3.690748	3.28989	0.497918
C	-2.511337	2.70881	-0.018947
C	-1.317381	3.434868	-0.005442
C	-1.306676	4.7619	0.509501
C	0.021929	5.2006	0.443036
H	0.412982	6.140833	0.796712
C	0.815725	4.162269	-0.074996
C	-2.506078	5.388127	1.017671
C	-3.716699	4.637494	1.01392
C	-4.890864	5.227438	1.511052
H	-5.817263	4.666559	1.509145
C	-4.876498	6.522839	1.995718
H	-5.788321	6.968838	2.372471
C	-3.685292	7.261335	1.998518
H	-3.680694	8.275584	2.377512
C	-2.515712	6.700102	1.516858
H	-1.602304	7.282151	1.521162
C	2.214166	4.133262	-0.204702
C	2.921932	5.431054	-0.192195
C	3.893619	5.728128	0.771745
C	4.552846	6.948715	0.790556
C	4.253089	7.905562	-0.177956
C	3.292709	7.638761	-1.152238
C	2.636367	6.416554	-1.1456
C	2.984837	2.964475	-0.31496
C	4.354687	2.850607	-0.636057
H	4.984688	3.682412	-0.904706
C	4.717979	1.50404	-0.56661
C	3.540811	0.789909	-0.215548
C	3.562754	-0.627803	-0.059847
C	4.869057	-1.289595	-0.187862
C	4.606221	-2.615977	0.009669
H	5.297591	-3.441931	-0.023002
C	3.176347	-2.73621	0.204202
C	6.079138	-0.561107	-0.522131
C	5.999663	0.839274	-0.743524
C	7.155691	1.547698	-1.092073
H	7.104045	2.616002	-1.263355
C	8.373316	0.894746	-1.219327
H	9.260244	1.453847	-1.489578
C	8.455432	-0.482807	-0.995406
H	9.406823	-0.990831	-1.091084
C	7.32011	-1.200548	-0.6516
H	7.393001	-2.267375	-0.480213
C	2.486542	-3.923949	0.302809

C	3.263819	-5.190262	0.382677
C	3.966745	-5.525393	1.540479
C	4.69985	-6.701736	1.633855
C	4.742595	-7.569764	0.545914
C	4.05393	-7.257552	-0.624185
C	3.324722	-6.078297	-0.691484
C	1.05735	-4.048135	0.277119
C	0.302554	-5.229563	0.206721
H	0.711035	-6.224947	0.239551
C	-1.043523	-4.871132	0.044883
C	-1.085167	-3.445689	0.065562
C	-2.308215	-2.799144	-0.077099
C	-3.500418	-3.570056	-0.334951
C	-4.535051	-2.663244	-0.460838
H	-5.565013	-2.880376	-0.692057
C	-4.028332	-1.345605	-0.257799
C	-3.468312	-5.017985	-0.39308
C	-2.228427	-5.674774	-0.168369
C	-2.183352	-7.07623	-0.189337
H	-1.244261	-7.587071	-0.017472
C	-3.331426	-7.815529	-0.428625
H	-3.283473	-8.897116	-0.441326
C	-4.550061	-7.168048	-0.655219
H	-5.444581	-7.748457	-0.842742
C	-4.616655	-5.783038	-0.636485
H	-5.567056	-5.292579	-0.808065
C	-4.728801	-0.15485	-0.224606
C	-6.206885	-0.187088	-0.26572
C	-6.961744	-0.857947	0.702902
C	-8.349547	-0.861603	0.673469
C	-9.016218	-0.1868	-0.347176

C	-8.292292	0.49147	-1.326524
C	-6.906377	0.488655	-1.272486
F	1.740028	6.171272	-2.106683
F	3.018376	8.54545	-2.080199
F	4.879048	9.067502	-0.171131
F	5.453673	7.212628	1.727343
F	4.171645	4.841596	1.732446
F	3.929816	-4.707633	2.597898
F	5.353061	-7.001591	2.750126
F	5.439807	-8.691811	0.62207
F	4.103608	-8.080449	-1.665832
F	2.683827	-5.782807	-1.830353
F	-6.3473	-1.484317	1.713155
F	-9.04032	-1.492975	1.614016
F	-10.336214	-0.189317	-0.386531
F	-8.92781	1.123937	-2.303864
F	-6.227942	1.124384	-2.233905
N	-2.763432	1.383249	-0.339943
H	-2.286471	0.905427	-1.092259
N	2.508262	1.665047	-0.089676
H	1.649917	1.448761	0.394524
N	2.562596	-1.464263	0.160081
N	0.199152	-2.975702	0.213899
H	0.567679	-2.027822	0.187274
H	0.188127	2.390089	-1.042635
N	-0.029343	3.063946	-0.322129
H	-2.093888	-0.762893	0.406351
N	-2.637531	-1.472786	-0.061541
Sum of electronic and thermal Free Energies= -4241.163342			
**H_32^{3+}**			

C	-3.108867	-2.845769	0.256752
C	-3.122281	-4.136769	-0.254093
H	-4.009627	-4.659263	-0.571834
C	-1.783763	-4.586936	-0.378243
C	-0.965709	-3.53184	0.083483
C	0.422241	-3.645224	-0.020618
C	0.995439	-4.85531	-0.550533
C	2.368707	-4.660503	-0.579233
H	3.113043	-5.339673	-0.962454
C	2.660313	-3.354813	-0.101088
C	0.167682	-5.961955	-0.980667
C	-1.247	-5.816629	-0.905002
C	-2.071178	-6.870631	-1.331948
H	-3.148074	-6.773548	-1.275045
C	-1.516519	-8.043995	-1.81603
H	-2.15955	-8.853329	-2.13777
C	-0.126122	-8.187433	-1.88631
H	0.302511	-9.107917	-2.262214
C	0.706584	-7.15717	-1.474147
H	1.780313	-7.286403	-1.53094
C	3.896855	-2.72532	-0.01549
C	5.120648	-3.530442	-0.090732
C	6.157179	-3.193551	-0.979736
C	7.325046	-3.933762	-1.057676
C	7.492669	-5.039486	-0.220176
C	6.487745	-5.397922	0.68109
C	5.318763	-4.654909	0.730455
C	4.045726	-1.307815	0.162388
C	5.122416	-0.625428	0.720492
H	5.997889	-1.098409	1.134588
C	4.829315	0.758145	0.706102
C	3.546217	0.887823	0.114953
C	2.953338	2.146656	0.024045
C	3.663137	3.295135	0.503442
C	2.811241	4.38228	0.332935
H	2.990064	5.399314	0.643228
C	1.582253	3.928466	-0.207179
C	4.983552	3.187241	1.085275
C	5.5713	1.893743	1.193808
C	6.848889	1.764959	1.76276
H	7.307374	0.787716	1.847592
C	7.534621	2.881872	2.210043
H	8.520868	2.772853	2.643079
C	6.957512	4.152666	2.100645
H	7.499585	5.022716	2.449139
C	5.695622	4.303076	1.545766
H	5.265456	5.293614	1.464191
C	0.412475	4.670388	-0.359492
C	0.534459	6.139534	-0.373267
C	1.336282	6.780013	-1.331265
C	1.457013	8.160961	-1.378141
C	0.784009	8.939616	-0.435501
C	-0.008096	8.331755	0.541557
C	-0.128249	6.951186	0.55971
C	-0.895326	4.101951	-0.443133
C	-2.110049	4.767587	-0.64166
H	-2.203682	5.812682	-0.885511
C	-3.16094	3.845381	-0.454178
C	-2.544797	2.592852	-0.206055
C	-3.33506	1.461302	-0.006288
C	-4.759518	1.609959	0.126016
C	-5.257819	0.342416	0.385343

H	-6.282784	0.077968	0.586382
C	-4.185589	-0.596861	0.337352
C	-5.403692	2.894906	-0.063666
C	-4.595681	4.014295	-0.418142
C	-5.207561	5.252139	-0.664952
H	-4.604452	6.108573	-0.939455
C	-6.582717	5.391465	-0.557151
H	-7.044859	6.351391	-0.749591
C	-7.373844	4.295827	-0.197443
H	-8.447138	4.409778	-0.112133
C	-6.790067	3.060118	0.044292
H	-7.417542	2.219152	0.312606
C	-4.249671	-1.980655	0.389332
C	-5.562157	-2.646146	0.485212
C	-6.583841	-2.438957	-0.452578
C	-7.801912	-3.095699	-0.360267
C	-8.029057	-3.978585	0.697266
C	-7.031712	-4.207834	1.647052
C	-5.816391	-3.552395	1.525981
F	4.392978	-4.993249	1.63198
F	6.659906	-6.432363	1.486199
F	8.598785	-5.744651	-0.281411
F	8.269836	-3.610661	-1.924146
F	5.997638	-2.167133	-1.82005
F	1.96737	6.053921	-2.258273
F	2.197277	8.738882	-2.308795
F	0.898587	10.248701	-0.463605
F	-0.624083	9.071399	1.44894
F	-0.853071	6.387051	1.532065
F	-6.374431	-1.633193	-1.500175
F	-8.739939	-2.899337	-1.272351

F	-9.184414	-4.597934	0.796799
F	-7.252077	-5.035872	2.654012
F	-4.878915	-3.761768	2.455486
N	-1.772087	-2.445904	0.44251
H	-1.543921	-1.88581	1.255581
N	3.054045	-0.378504	-0.170741
H	2.409299	-0.562529	-0.926687
N	-1.170777	2.747659	-0.232077
H	-0.544663	2.167014	0.307078
H	1.358213	-1.934032	0.801245
N	1.426975	-2.748294	0.209937
H	-2.162046	-0.278258	-0.224776
N	-3.005163	0.144748	0.134434
H	1.193782	2.06403	-1.121371
N	1.68794	2.527211	-0.370325
Sum of electronic and thermal Free Energies= -4241.558330			
**$H_3 2^{\bullet 2+}$**			
F	-2.095333	-5.91171	-2.438192
F	-3.405257	-8.273912	-2.506693
F	-5.087624	-8.95462	-0.482716
F	-5.448926	-7.272254	1.62113
F	-4.131072	-4.918511	1.71364
F	-2.0716	5.919287	2.43946
F	-3.371357	8.287104	2.508333
F	-5.050093	8.975734	0.484018
F	-5.417927	7.295656	-1.620513
F	-4.110201	4.936284	-1.71339
F	6.305577	1.381583	1.912941
F	9.001636	1.381861	1.895578
F	10.352084	-0.020621	0.000047

F	8.996436	-1.417925	-1.895593
F	6.3004	-1.407279	-1.913226
N	2.713709	-1.437766	-0.151494
H	2.217547	-0.840207	-0.795896
N	-0.075014	-3.026067	-0.346583
H	-0.237944	-2.35526	-1.085798
N	-2.549889	-1.584406	-0.184729
H	-1.670452	-1.457592	0.295273
N	-2.543723	1.594793	0.185393
H	-1.663073	1.46389	-0.291198
N	-0.062761	3.025826	0.347218
H	-0.227219	2.358283	1.089143
N	2.719588	1.426498	0.151437
H	2.220632	0.83049	0.795096
C	4.07945	-1.246931	0.113269
C	4.60297	-2.480436	0.543556
H	5.616193	-2.621489	0.882749
C	3.580353	-3.435122	0.521823
C	2.410644	-2.768669	0.051481
C	1.207816	-3.46182	-0.036134
C	1.150122	-4.825708	0.356592
C	-0.189567	-5.221956	0.231786
H	-0.608495	-6.178687	0.497671
C	-0.948113	-4.124664	-0.206508
C	3.553522	-4.827636	0.910386
C	4.698084	-5.50081	1.365278
H	5.643028	-4.975293	1.427028
C	4.632135	-6.834233	1.730556
H	5.522685	-7.343604	2.076716
C	3.4175	-7.527735	1.651648
H	3.372378	-8.571232	1.936959

C	2.275715	-6.883685	1.207715
H	1.342868	-7.430392	1.146262
C	2.319408	-5.532474	0.829535
C	-2.350678	-4.054631	-0.334441
C	-3.085106	-5.342471	-0.367586
C	-2.91014	-6.236486	-1.427911
C	-3.579673	-7.451256	-1.480037
C	-4.445733	-7.799534	-0.445808
C	-4.634858	-6.932928	0.628596
C	-3.95766	-5.721455	0.656246
C	-3.096569	-2.866225	-0.354528
C	-4.498145	-2.717339	-0.453391
H	-5.181787	-3.529027	-0.639412
C	-4.822352	-1.372499	-0.258402
C	-3.587392	-0.680742	-0.091969
C	-3.584594	0.695365	0.092107
C	-4.816911	1.392275	0.257541
C	-4.487336	2.735698	0.452747
H	-5.167746	3.550266	0.637987
C	-3.084981	2.878771	0.354943
C	-6.091824	-0.682428	-0.1566
C	-7.317382	-1.346462	-0.312266
H	-7.330737	-2.402865	-0.550625
C	-8.513246	-0.664512	-0.159534
H	-9.452689	-1.188859	-0.281305
C	-8.510584	0.699367	0.155468
H	-9.447978	1.227552	0.276445
C	-7.312071	1.376426	0.309212
H	-7.32131	2.432875	0.547561
C	-6.089106	0.707394	0.154599
C	-2.334183	4.064059	0.335097

C	-3.063182	5.354997	0.368377
C	-2.884701	6.247908	1.429041
C	-3.548994	7.465541	1.481355
C	-4.413192	7.817893	0.446951
C	-4.605682	6.952474	-0.627811
C	-3.933716	5.738089	-0.655642
C	-0.931318	4.128311	0.207163
C	-0.168441	5.222311	-0.231487
H	-0.583493	6.180671	-0.497598
C	1.169684	4.820571	-0.356525
C	1.221912	3.456627	0.036394
C	2.421838	2.758541	-0.051553
C	3.594208	3.420227	-0.522117
C	4.612935	2.461413	-0.543939
H	5.62669	2.598343	-0.883226
C	4.084439	1.230039	-0.113571
C	2.341638	5.522575	-0.829863
C	2.303275	6.873904	-1.208246
H	1.372626	7.424337	-1.146737
C	3.44755	7.513321	-1.652431
H	3.406567	8.556949	-1.937884
C	4.659399	6.81495	-1.731419
H	5.551916	7.320719	-2.077792
C	4.720089	5.481327	-1.365979
H	5.662915	4.952033	-1.427828
C	3.57292	4.812788	-0.910806
C	4.74131	-0.009781	-0.000179
C	6.218867	-0.012706	-0.000133
C	6.949089	0.704807	0.955014
C	8.336516	0.706499	0.966718
C	9.030937	-0.018101	-0.000002

C	8.333827	-0.740033	-0.966784
C	6.946412	-0.733018	-0.955211
Sum of electronic and thermal Free Energies=-4241.780449			
**H_32^+**			
C	-0.948472	-4.130945	-0.169592
C	-0.16516	-5.24668	0.190495
H	-0.570103	-6.220115	0.413754
C	1.169468	-4.850033	0.289112
C	1.216145	-3.463311	-0.033644
C	2.408736	-2.773914	0.041426
C	3.605393	-3.449486	0.429601
C	4.621304	-2.49499	0.454911
H	5.649404	-2.647367	0.739883
C	4.077782	-1.240513	0.102072
C	4.729743	-0.002373	-0.000067
C	4.079041	1.236416	-0.102187
C	4.623856	2.490386	-0.454911
H	5.652114	2.641736	-0.739858
C	3.60892	3.4459	-0.429568
C	2.411556	2.771505	-0.041479
C	1.219675	3.462108	0.033639
C	1.174396	4.848896	-0.289017
C	-0.159833	5.246874	-0.190402
H	-0.563796	6.220734	-0.41359
C	-0.944281	4.131894	0.169577
C	-2.341452	4.062262	0.287372
C	-3.092074	2.880655	0.312682
C	-4.498201	2.736616	0.395666
H	-5.180715	3.555892	0.550457
C	-4.823872	1.393415	0.224649

C	-3.593156	0.685635	0.079746	H	1.415316	7.481601	-0.946006
C	-3.593845	-0.682025	-0.079971	C	2.368528	5.566173	-0.683378
C	-4.82528	-1.388562	-0.22492	C	-3.079749	5.352112	0.305698
C	-4.500974	-2.732094	-0.395849	C	-3.001896	6.21087	1.40317
H	-5.184309	-3.550686	-0.550646	C	-3.675494	7.42493	1.436827
C	-3.094993	-2.877558	-0.312783	C	-4.454502	7.805065	0.348735
C	-2.345576	-4.059922	-0.287416	C	-4.551008	6.972333	-0.761427
C	2.362865	-5.568485	0.683541	C	-3.864758	5.764807	-0.771894
C	2.341602	-6.936711	0.998621	C	-6.094579	0.70204	0.13446
H	1.407719	-7.482936	0.946265	C	-7.320145	1.37256	0.268298
C	3.500983	-7.595144	1.369039	H	-7.327693	2.435924	0.474555
H	3.470155	-8.651351	1.606464	C	-8.51798	0.691116	0.135884
C	4.714648	-6.89839	1.435718	H	-9.455961	1.222223	0.240853
H	5.620511	-7.416918	1.724604	C	-8.518673	-0.682494	-0.136362
C	4.75877	-5.549113	1.131717	H	-9.457188	-1.212644	-0.241384
H	5.701347	-5.017934	1.183437	C	-7.321525	-1.36516	-0.268706
C	3.595322	-4.860726	0.751975	H	-7.330146	-2.428517	-0.474958
C	6.21273	-0.003142	-0.00007	C	-6.095285	-0.695889	-0.1348
C	6.942047	-0.616051	-1.021985	C	-3.08517	-5.349014	-0.305661
C	8.330525	-0.624796	-1.032322	C	-3.870782	-5.760729	0.771867
C	9.027095	-0.004655	-0.000061	C	-4.55828	-6.967545	0.761484
C	8.331185	0.616237	1.032191	C	-4.462444	-7.800567	-0.348517
C	6.942697	0.608988	1.021846	C	-3.68286	-7.421413	-1.436541
C	3.600276	4.85718	-0.751829	C	-3.008026	-6.208041	-1.402978
C	4.764423	5.544419	-1.131499	N	-0.082845	-3.023885	-0.280352
H	5.706465	5.012294	-1.183242	H	-0.273629	-2.318815	-0.979051
C	4.721668	6.893763	-1.435409	N	2.702295	-1.427622	-0.110858
H	5.628059	7.411396	-1.724243	H	2.192693	-0.818066	-0.733317
C	3.508703	7.591732	-1.368715	N	2.703769	1.424934	0.110738
H	3.478943	8.647985	-1.606071	H	2.193372	0.815678	0.732829
C	2.348651	6.934439	-0.998369	N	-0.079764	3.023963	0.280316

H	-0.271222	2.319159	0.979102
N	-2.548352	1.592482	0.176865
H	-1.685293	1.463111	-0.332558
N	-2.549984	-1.589945	-0.176992
H	-1.686683	-1.461374	0.332221
F	6.299754	-1.199221	-2.041968
F	8.996932	-1.210359	-2.026152
F	10.355236	-0.005374	-0.000054
F	8.998214	1.201082	2.026028
F	6.301025	1.192841	2.041826
F	-2.269538	5.862513	2.468439
F	-3.587394	8.220151	2.50183
F	-5.105012	8.963124	0.369182
F	-5.289269	7.341404	-1.807507
F	-3.957231	4.99377	-1.863176
F	-3.962652	-4.98942	1.863008
F	-5.297095	-7.335674	1.807504
F	-5.114149	-8.957954	-0.368883
F	-3.595403	-8.2169	-2.5014
F	-2.275155	-5.860609	-2.468196
Sum of electronic and thermal Free Energies= -4241.969392			
**1_a^{2+}**			
C	-1.207246	-4.474065	-0.110991
C	-0.880819	-5.772413	-0.559181
C	0.523841	-5.885157	-0.567188
C	1.052407	-4.64447	-0.134187
C	-1.840119	-6.760224	-1.158689
H	-2.850421	-6.56258	-0.804028
H	-1.583378	-7.771585	-0.831506
C	-1.847316	-6.700186	-2.695692
H	-2.55591	-7.427347	-3.101926
H	-2.146353	-5.705915	-3.039625
H	-0.86099	-6.918157	-3.111439
C	1.276711	-7.076689	-1.084685
H	0.551096	-7.821231	-1.414638
H	1.831426	-7.547997	-0.269167
C	2.230975	-6.762936	-2.24486
H	2.713686	-7.678662	-2.596579
H	1.688123	-6.321367	-3.085106
H	3.013802	-6.063414	-1.945045
C	2.375308	-4.162118	0.071318
C	3.567769	-4.764017	0.542103
C	4.565041	-3.768307	0.566575
C	3.989855	-2.571502	0.08772
C	3.744264	-6.157206	1.070408
H	3.59185	-6.879688	0.265514
H	4.783189	-6.276967	1.38069
C	2.824302	-6.497759	2.251123
H	3.02648	-7.51019	2.610922
H	2.986784	-5.803726	3.080373
H	1.771191	-6.443964	1.967652
C	5.911754	-3.895181	1.2197
H	6.544598	-3.057983	0.928857
H	6.410183	-4.804392	0.870944
C	5.816205	-3.916107	2.754938
H	6.814067	-3.992129	3.195867
H	5.350909	-2.997453	3.123395
H	5.223318	-4.761208	3.111736
C	4.559556	-1.285312	-0.135396
C	5.80263	-0.907325	-0.684554
C	5.874987	0.5027	-0.647087

C	4.662919	0.977232	-0.100129
C	6.726546	-1.805534	-1.457922
H	6.700848	-2.81867	-1.059556
H	7.755333	-1.455106	-1.358561
C	6.344989	-1.8627	-2.947815
H	7.042066	-2.502717	-3.495991
H	5.338772	-2.272357	-3.072354
H	6.365113	-0.869945	-3.403942
C	6.975729	1.329588	-1.245993
H	6.630947	2.358625	-1.35127
H	7.170034	0.967778	-2.260687
C	8.295683	1.300336	-0.459513
H	9.038762	1.938091	-0.945929
H	8.162371	1.656894	0.56322
H	8.705312	0.289241	-0.406782
C	4.145956	2.288697	0.133643
C	4.708197	3.464474	0.676088
C	3.692869	4.446115	0.707078
C	2.527179	3.871763	0.161439
C	6.077445	3.613297	1.273635
H	5.972227	4.004006	2.290686
H	6.531533	2.627064	1.372169
C	7.019397	4.542145	0.491919
H	8.000085	4.576393	0.974118
H	7.159347	4.199893	-0.534864
H	6.630655	5.561951	0.451868
C	3.752835	5.746869	1.456891
H	4.733122	6.20873	1.326128
H	3.019334	6.447528	1.058896
C	3.47165	5.554163	2.95773
H	3.537487	6.510493	3.484121
H	2.467461	5.149766	3.112513
H	4.18739	4.866031	3.413696
C	1.244456	4.441615	-0.09179
C	0.898555	5.692354	-0.642722
C	-0.509811	5.783898	-0.646291
C	-1.015207	4.57283	-0.119115
C	1.842027	6.606327	-1.372614
H	1.527671	7.644006	-1.251414
H	2.843424	6.530911	-0.951141
C	1.922325	6.264589	-2.870848
H	2.60077	6.952982	-3.382529
H	2.299279	5.248094	-3.014131
H	0.94357	6.333783	-3.351356
C	-1.304888	6.901023	-1.262695
H	-0.965732	7.041669	-2.294023
H	-2.353454	6.606619	-1.327713
C	-1.185972	8.246477	-0.526673
H	-1.859075	8.981606	-0.975931
H	-1.443156	8.153738	0.531294
H	-0.171423	8.644824	-0.582992
C	-2.346521	4.087932	0.041916
C	-3.54538	4.725487	0.4346
C	-4.587702	3.784035	0.335943
C	-4.019968	2.561771	-0.099425
C	-3.660816	6.078549	1.071009
H	-2.800489	6.685552	0.808036
H	-4.540403	6.598765	0.681533
C	-3.746196	5.995982	2.604355
H	-3.82213	6.998075	3.035734
H	-4.615808	5.41983	2.928406
H	-2.852349	5.516508	3.013627

C	-6.005511	4.034804	0.760391
H	-6.09373	5.079359	1.06199
H	-6.674577	3.917321	-0.094825
C	-6.482996	3.140393	1.913034
H	-7.508746	3.396603	2.19148
H	-6.462091	2.084114	1.63772
H	-5.8493	3.273251	2.794027
C	-4.579883	1.275629	-0.338215
C	-5.83432	0.845599	-0.83528
C	-5.863081	-0.56217	-0.756964
C	-4.620803	-0.991682	-0.238783
C	-6.892099	1.677993	-1.503574
H	-7.618688	1.00191	-1.954997
H	-7.449921	2.249542	-0.755962
C	-6.363871	2.618945	-2.594819
H	-7.194544	3.152831	-3.064116
H	-5.669255	3.361167	-2.198632
H	-5.844053	2.052616	-3.372957
C	-6.959721	-1.438893	-1.28787
H	-6.608705	-2.470034	-1.317668
H	-7.156402	-1.157685	-2.328504
C	-8.277321	-1.357155	-0.502295
H	-9.011212	-2.050114	-0.922349
H	-8.133697	-1.612843	0.549095
H	-8.703087	-0.352188	-0.542345
C	-4.088888	-2.286051	0.042219
C	-4.643706	-3.425353	0.668159
C	-3.633809	-4.407611	0.73517
C	-2.478251	-3.875319	0.124881
C	-5.996333	-3.526865	1.312227
H	-5.86176	-3.834012	2.354283

H	-6.451657	-2.537063	1.345785
C	-3.678392	-5.652637	1.575351
H	-4.6649	-6.114133	1.505415
C	-3.352711	-5.357066	3.050191
H	-3.405536	-6.274726	3.642671
H	-2.343169	-4.948106	3.147694
H	-4.053063	-4.636156	3.478366
N	-0.022213	-3.787792	0.062639
H	0.047447	-2.952322	0.628592
N	2.649891	-2.817459	-0.131262
H	2.075568	-2.225073	-0.717052
N	3.858235	-0.126474	0.137979
H	3.070913	-0.099022	0.772556
N	2.803954	2.545724	-0.105755
H	2.246168	1.98587	-0.737618
N	0.071194	3.752188	0.14546
H	0.018331	2.957753	0.769324
N	-2.652298	2.762002	-0.201077
H	-2.045436	2.146894	-0.72729
N	-3.839204	0.136792	-0.065018
H	-3.03322	0.145418	0.545784
N	-2.753624	-2.563828	-0.212339
H	-2.209485	-2.047075	-0.891214
H	-2.961928	-6.385892	1.207854
C	-6.956262	-4.51714	0.634771
H	-6.568639	-5.537356	0.67255
H	-7.924662	-4.508499	1.142056
H	-7.121698	-4.263865	-0.413879
Sum of electronic and thermal Free Energies= -2929.501694			
**$1_a^{\bullet 3+}$**			

C	0.63473	-4.428693	0.280853
C	0.144522	-5.578357	0.927157
C	-1.266623	-5.502001	0.918687
C	-1.615762	-4.303432	0.258128
C	0.97173	-6.596156	1.657154
H	1.984822	-6.603521	1.254793
H	0.55638	-7.593085	1.48595
C	1.049779	-6.324929	3.168498
H	1.670649	-7.080875	3.656769
H	1.494223	-5.344377	3.362138
H	0.061654	-6.345937	3.632319
C	-2.185333	-6.406301	1.688892
H	-1.627867	-7.29504	1.988965
H	-3.005845	-6.755599	1.061304
C	-2.774719	-5.725872	2.935443
H	-3.406592	-6.426985	3.486995
H	-1.983595	-5.380661	3.605556
H	-3.387609	-4.864354	2.658277
C	-2.877803	-3.715049	-0.037111
C	-4.092811	-4.286458	-0.505548
C	-5.055674	-3.273169	-0.535743
C	-4.419797	-2.06066	-0.113697
C	-4.238587	-5.657552	-1.098708
H	-3.431909	-6.298527	-0.748498
H	-5.174635	-6.109816	-0.761204
C	-4.197941	-5.631826	-2.636515
H	-4.28399	-6.647664	-3.03092
H	-5.013894	-5.036673	-3.051718
H	-3.25375	-5.208889	-2.990457
C	-6.438727	-3.437011	-1.089263
H	-7.175489	-3.211675	-0.316697

H	-6.583179	-4.488702	-1.338016
C	-6.711854	-2.581413	-2.335102
H	-7.717409	-2.781286	-2.71321
H	-6.63955	-1.514568	-2.114092
H	-5.997644	-2.814162	-3.129152
C	-4.856215	-0.73422	0.027696
C	-6.116779	-0.129993	0.353745
C	-5.943655	1.25406	0.324769
C	-4.576397	1.517626	-0.00197
C	-7.350894	-0.815295	0.8596
H	-7.81203	-1.39761	0.059294
H	-8.079566	-0.050526	1.125419
C	-7.104843	-1.704442	2.088181
H	-8.052203	-2.118724	2.441523
H	-6.436555	-2.53779	1.8641
H	-6.664178	-1.123521	2.902874
C	-6.96871	2.262857	0.752711
H	-6.48057	3.222107	0.914499
H	-7.358874	1.954757	1.72834
C	-8.14995	2.430228	-0.215772
H	-8.833601	3.195635	0.159622
H	-7.816669	2.732127	-1.210136
H	-8.711731	1.499994	-0.322842
C	-3.828526	2.714879	-0.143147
C	-4.126414	3.991271	-0.683328
C	-2.956644	4.771663	-0.589358
C	-1.962514	3.977382	0.015248
C	-5.371841	4.408813	-1.409333
H	-5.075893	4.792063	-2.391074
H	-5.989917	3.533371	-1.60277
C	-6.200026	5.490745	-0.697887

H	-7.100949	5.709113	-1.276785
H	-6.508369	5.175792	0.300508
H	-5.636927	6.420105	-0.592667
C	-2.720606	6.094149	-1.2608
H	-3.636858	6.685124	-1.244154
H	-1.967406	6.664496	-0.717264
C	-2.248481	5.915691	-2.714005
H	-2.096397	6.88943	-3.1872
H	-1.301885	5.369084	-2.748737
H	-2.982734	5.362085	-3.304526
C	-0.615194	4.335607	0.360329
C	-0.141454	5.494005	1.004245
C	1.270835	5.449867	0.971172
C	1.63617	4.245771	0.32588
C	-0.995464	6.448342	1.78975
H	-0.612889	7.464738	1.689222
H	-2.009043	6.45257	1.388137
C	-1.067984	6.07152	3.27958
H	-1.700593	6.78234	3.817998
H	-1.496882	5.073354	3.405609
H	-0.080867	6.077737	3.746875
C	2.197203	6.420617	1.648149
H	1.916374	6.484236	2.703808
H	3.212675	6.022266	1.631767
C	2.185515	7.840076	1.05574
H	2.947429	8.452311	1.544904
H	2.392836	7.835248	-0.016814
H	1.221077	8.329132	1.201541
C	2.902684	3.656274	0.05778
C	4.142241	4.225572	-0.353775
C	5.091251	3.202532	-0.391233
C	4.43015	1.98479	-0.022124
C	4.331739	5.613252	-0.887833
H	3.555359	6.266167	-0.502821
H	5.285122	6.016985	-0.538678
C	4.281416	5.651069	-2.424861
H	4.402384	6.677463	-2.780868
H	5.073061	5.041806	-2.86677
H	3.320577	5.276293	-2.788416
C	6.488756	3.36937	-0.907094
H	6.643962	4.425575	-1.127714
H	7.20619	3.121976	-0.123119
C	6.7869	2.545212	-2.168257
H	7.799893	2.755171	-2.520241
H	6.711448	1.472746	-1.977952
H	6.089324	2.799236	-2.970534
C	4.856423	0.653879	0.100834
C	6.110331	0.043647	0.443729
C	5.943849	-1.338875	0.370339
C	4.585487	-1.597884	0.006893
C	7.327249	0.715777	1.006341
H	8.045925	-0.055279	1.280637
H	7.815639	1.313346	0.233913
C	7.039694	1.579012	2.244301
H	7.972711	1.996762	2.630157
H	6.366648	2.408341	2.019355
H	6.584827	0.977339	3.035897
C	6.967266	-2.355347	0.782332
H	6.484355	-3.323135	0.901804
H	7.340456	-2.080294	1.774323
C	8.162277	-2.476892	-0.175921
H	8.845056	-3.254368	0.175371

H	7.842776	-2.737111	-1.186442
H	8.719762	-1.53966	-0.235838
C	3.846689	-2.795391	-0.171916
C	4.155544	-4.05985	-0.734974
C	2.987863	-4.846748	-0.669359
C	1.984694	-4.069659	-0.057103
C	5.410715	-4.457244	-1.456381
H	5.12835	-4.815783	-2.451385
H	6.030125	-3.576568	-1.619172
C	2.763421	-6.155868	-1.370459
H	3.678974	-6.747843	-1.349815
C	2.315273	-5.949877	-2.827718
H	2.170435	-6.914426	-3.321505
H	1.369613	-5.40201	-2.867777
H	3.059229	-5.385981	-3.395868
N	-0.440987	-3.630433	-0.039895
H	-0.390692	-2.897316	-0.736379
N	-3.075104	-2.364591	0.09783
H	-2.474287	-1.795506	0.682248
N	-3.939693	0.301208	-0.096933
H	-3.063045	0.191179	-0.590512
N	-2.478786	2.718877	0.20395
H	-2.080262	2.059258	0.861344
N	0.468532	3.552468	0.036898
H	0.425381	2.816711	-0.657157
N	3.084172	2.299883	0.158515
H	2.451666	1.709445	0.684832
N	3.945243	-0.381043	-0.071083
H	3.086458	-0.264317	-0.594026
N	2.493897	-2.813562	0.161943
H	2.086989	-2.172356	0.83201

H	2.00144	-6.736379	-0.850464
C	6.23274	-5.554179	-0.760811
H	5.67374	-6.489316	-0.691157
H	7.145047	-5.751083	-1.329492
H	6.52138	-5.264867	0.251165
Sum of electronic and thermal Free Energies= -2929.302836			
**H_32^{3+}**•$CF_3CO_2^-$			
F	-6.141762	-2.071361	-2.250577
F	-8.817149	-2.377706	-2.21681
F	-10.30332	-1.164479	-0.294562
F	-9.105471	0.355776	1.610131
F	-6.427942	0.658908	1.609544
F	3.044308	5.388886	2.471954
F	4.143231	7.863603	2.506686
F	4.046448	9.421175	0.281002
F	2.85519	8.502279	-1.982545
F	1.760743	6.030595	-2.028242
F	4.884238	-4.336804	1.471395
F	6.385362	-6.560976	1.241117
F	5.972134	-8.276866	-0.822703
F	4.051189	-7.76086	-2.672365
F	2.549737	-5.528323	-2.472117
N	0.436092	-2.910222	-0.473297
H	0.577096	-1.995559	-0.872729
N	-2.54868	-1.690084	-0.489084
H	-1.996232	-0.906129	-0.801149
N	-2.910729	1.139089	-0.137552
H	-2.356618	0.62941	0.586638
N	-0.357794	2.987733	0.087766
H	-0.047938	2.021958	0.155267

N	2.446588	1.877676	0.273526
H	1.601598	1.405341	0.59046
N	2.830221	-1.202979	-0.059217
H	1.908735	-1.055317	0.350792
C	1.42295	-3.863434	-0.263653
C	0.793673	-5.045269	0.151754
H	1.313838	-5.947943	0.42507
C	-0.591346	-4.815657	0.205477
C	-0.787446	-3.46984	-0.199194
C	-2.085669	-2.932908	-0.209105
C	-3.191991	-3.761466	0.188293
C	-4.320369	-2.957566	0.132245
H	-5.327597	-3.230165	0.400591
C	-3.93171	-1.649893	-0.269278
C	-1.689575	-5.674859	0.594237
C	-1.491839	-7.008957	0.979197
H	-0.491735	-7.424163	0.990569
C	-2.566397	-7.804146	1.343111
H	-2.401356	-8.832836	1.63773
C	-3.864152	-7.28138	1.328639
H	-4.701848	-7.906204	1.611655
C	-4.080865	-5.965885	0.950963
H	-5.09019	-5.573178	0.940123
C	-3.007148	-5.143915	0.580873
C	-4.735172	-0.513558	-0.339335
C	-6.19975	-0.693543	-0.321805
C	-6.848726	-1.484215	-1.280111
C	-8.226894	-1.64242	-1.284188
C	-8.992349	-1.013855	-0.303526
C	-8.376076	-0.226885	0.668429
C	-6.998225	-0.070213	0.64717

C	-4.234482	0.817188	-0.414736
C	-4.921489	1.991205	-0.768303
H	-5.951612	2.031192	-1.08251
C	-4.019734	3.067637	-0.666099
C	-2.783321	2.496439	-0.275375
C	-1.654267	3.311524	-0.127872
C	-1.786099	4.735178	-0.242723
C	-0.519534	5.25029	-0.026432
H	-0.233503	6.288514	-0.01843
C	0.402306	4.167907	0.144951
C	-4.175121	4.49855	-0.842531
C	-5.397219	5.073919	-1.216997
H	-6.252295	4.438926	-1.413583
C	-5.524845	6.449451	-1.333467
H	-6.475049	6.880776	-1.622129
C	-4.430194	7.280293	-1.075585
H	-4.533393	8.354366	-1.164842
C	-3.210565	6.732107	-0.708536
H	-2.368289	7.385344	-0.515639
C	-3.058521	5.344769	-0.586967
C	1.78568	4.280934	0.220116
C	2.361769	5.654446	0.228441
C	2.994307	6.14592	1.371776
C	3.560684	7.413335	1.403661
C	3.508926	8.213772	0.264231
C	2.89317	7.743627	-0.893897
C	2.327976	6.475333	-0.899665
C	2.739925	3.224651	0.197885
C	4.116858	3.34559	-0.106837
H	4.625359	4.281809	-0.26536
C	4.653002	2.053713	-0.241921

C	3.576529	1.169642	0.005651
C	3.738331	-0.20954	-0.153159
C	5.010108	-0.739745	-0.549467
C	4.823802	-2.109177	-0.669652
H	5.54721	-2.842075	-0.987554
C	3.461447	-2.4162	-0.357529
C	5.965902	1.548772	-0.605474
C	7.057414	2.402349	-0.810309
H	6.932428	3.471329	-0.68838
C	8.299688	1.89346	-1.159374
H	9.134647	2.565889	-1.310983
C	8.476077	0.515438	-1.311875
H	9.44734	0.120377	-1.581616
C	7.409479	-0.348562	-1.113954
H	7.558454	-1.415378	-1.229105
C	6.145698	0.142623	-0.76136
C	2.832476	-3.652117	-0.379737
C	3.67197	-4.862212	-0.497092
C	4.67916	-5.151616	0.432787
C	5.452513	-6.299851	0.335472
C	5.236223	-7.186324	-0.718027
C	4.244656	-6.92351	-1.662411
C	3.474373	-5.777142	-1.539757
F	1.081204	-1.996351	2.382935
F	1.540156	-0.00944	3.156256
F	-0.179763	-1.104727	3.916357
O	0.348528	0.099613	0.640946
O	-1.527587	-0.06045	1.866695
C	-0.322344	-0.203593	1.685302
C	0.522487	-0.835278	2.8303
Sum of electronic and thermal Free Energies= -4768.155200			

**H_32^{3+}**$\bullet(CF_3CO_2^-)_2$			
F	4.40166	-4.689518	1.901271
F	6.603648	-6.233993	1.804754
F	8.368511	-5.970745	-0.247717
F	7.913145	-4.152394	-2.219986
F	5.711066	-2.609191	-2.153769
F	1.533842	5.92626	-2.630222
F	1.949666	8.58136	-2.822391
F	1.22738	10.228358	-0.780943
F	0.085669	9.202224	1.465541
F	-0.317013	6.549057	1.687063
F	-5.937928	-1.694882	-1.694183
F	-8.392044	-2.817574	-1.750013
F	-9.333429	-4.145678	0.432767
F	-7.805546	-4.346238	2.675042
F	-5.34974	-3.224868	2.744645
N	-1.791246	-2.394852	0.606364
H	-1.354571	-1.584727	1.065938
N	1.331233	-2.785659	0.307532
H	1.203893	-1.913248	0.833525
N	2.945722	-0.419058	-0.292401
H	2.118647	-0.606459	-0.908024
N	1.605913	2.495029	-0.447674
H	0.853841	1.854948	-0.723109
N	-1.250355	2.912811	0.086724
H	-0.652174	2.251506	0.610952
N	-3.008144	0.362568	0.425509
H	-2.103544	0.067018	0.049691
C	-3.144235	-2.703875	0.48096
C	-3.237596	-4.056505	0.053278

H	-4.16175	-4.563065	-0.174169
C	-1.94695	-4.54979	-0.10971
C	-1.070247	-3.487018	0.257471
C	0.321253	-3.659232	0.115716
C	0.838496	-4.884084	-0.400153
C	2.220747	-4.711393	-0.489595
H	2.944876	-5.411854	-0.874031
C	2.520838	-3.394488	-0.060626
C	-1.441404	-5.819164	-0.601828
C	-2.298358	-6.875946	-0.943885
H	-3.368482	-6.750898	-0.829706
C	-1.787998	-8.072756	-1.422606
H	-2.461258	-8.881119	-1.682084
C	-0.405321	-8.23777	-1.571182
H	-0.008627	-9.173859	-1.945925
C	0.458841	-7.205668	-1.239342
H	1.528033	-7.337064	-1.356361
C	-0.037063	-5.986897	-0.751792
C	3.769438	-2.746027	-0.07387
C	4.979443	-3.595536	-0.123397
C	5.236863	-4.550792	0.864159
C	6.371047	-5.345992	0.833787
C	7.277901	-5.20813	-0.208756
C	7.047383	-4.273617	-1.209439
C	5.913815	-3.478499	-1.156284
C	3.949144	-1.356087	-0.040719
C	5.125899	-0.656612	0.320918
H	6.05108	-1.121554	0.621411
C	4.838781	0.704384	0.290961
C	3.469779	0.817531	-0.113192
C	2.883449	2.102277	-0.204647
C	3.683156	3.250679	0.076699
C	2.838908	4.35167	-0.033691
H	3.089845	5.384151	0.146697
C	1.538515	3.882517	-0.334175
C	5.657113	1.862714	0.588504
C	6.998235	1.7466	0.986063
H	7.441288	0.762855	1.084514
C	7.754309	2.87721	1.25153
H	8.788571	2.775368	1.558181
C	7.183872	4.150302	1.123042
H	7.77721	5.033009	1.330289
C	5.860531	4.284988	0.733445
H	5.424433	5.272099	0.636458
C	5.077981	3.151633	0.462012
C	0.3798	4.685967	-0.351985
C	0.58876	6.150463	-0.468035
C	1.179755	6.709009	-1.602669
C	1.394232	8.073213	-1.718441
C	1.021438	8.916256	-0.68018
C	0.433736	8.390699	0.462434
C	0.221283	7.024097	0.556911
C	-0.933706	4.229406	-0.219674
C	-2.15314	4.948008	-0.375612
H	-2.225147	5.988046	-0.651612
C	-3.202729	4.059497	-0.142201
C	-2.589117	2.799735	0.120441
C	-3.377733	1.661143	0.345634
C	-4.788224	1.784247	0.448135
C	-5.267602	0.48493	0.621658
H	-6.293237	0.183433	0.760298
C	-4.161228	-0.405873	0.57145

C	-4.647492	4.206885	-0.073933
C	-5.278731	5.439124	-0.299528
H	-4.678698	6.303283	-0.560032
C	-6.655523	5.560996	-0.187002
H	-7.128336	6.520006	-0.362674
C	-7.433285	4.449268	0.156868
H	-8.50868	4.546865	0.246666
C	-6.830996	3.220268	0.381333
H	-7.437778	2.361111	0.641974
C	-5.440505	3.072937	0.271488
C	-4.21722	-1.807455	0.56029
C	-5.576862	-2.417336	0.531169
C	-6.383626	-2.329499	-0.601667
C	-7.64284	-2.907636	-0.646733
C	-8.1225	-3.589699	0.463373
C	-7.340035	-3.69391	1.6053
C	-6.080101	-3.115037	1.626703
F	1.324286	0.151922	4.403343
F	2.55715	-0.787386	2.876232
F	2.519914	1.374163	3.058885
O	0.06575	1.463257	1.96104
O	0.132009	-0.782831	1.738464
C	0.522971	0.330877	2.155268
C	1.752458	0.269816	3.12107
F	-2.50036	-0.719845	-2.406284
F	-1.096132	-0.665638	-4.064356
F	-1.219048	-2.428154	-2.792885
O	-0.494069	0.402705	-1.057248
O	0.995427	-0.929441	-2.096245
C	-0.139545	-0.470829	-1.875443
C	-1.256001	-1.077927	-2.784491

Sum of electronic and thermal Free Energies= -5294.732969			
**$H_3$2$^{\bullet 2+}$**$\bullet CF_3CO_2^-$			
F	-5.884199	-2.384441	-2.370015
F	-8.508436	-3.004246	-2.429516
F	-10.139688	-2.227241	-0.396927
F	-9.131116	-0.833366	1.708823
F	-6.504787	-0.227859	1.796762
F	2.198531	5.855331	2.454082
F	3.001729	8.440786	2.402355
F	3.033944	9.805892	0.049837
F	2.265407	8.580464	-2.253624
F	1.467156	5.993964	-2.208473
F	5.028539	-3.93528	1.713822
F	6.80763	-5.959708	1.584817
F	6.844028	-7.60663	-0.578781
F	5.097212	-7.213942	-2.625867
F	3.327017	-5.179235	-2.52169
N	0.70284	-2.856237	-0.40601
H	0.746817	-1.880765	-0.655119
N	-2.373678	-1.909227	-0.368099
H	-1.887846	-1.058055	-0.604773
N	-3.023366	0.87282	-0.020053
H	-2.410837	0.429599	0.693883
N	-0.656549	2.959712	0.150577
H	-0.255648	2.030553	0.201335
N	2.248352	2.106198	0.179759
H	1.446375	1.556109	0.473614
N	2.926042	-0.927223	-0.090463
H	2.005885	-0.857091	0.337503
C	1.784906	-3.723383	-0.268673

C	1.251226	-5.004936	0.006215
H	1.850604	-5.881318	0.18786
C	-0.138675	-4.911748	0.053464
C	-0.462158	-3.54737	-0.20927
C	-1.796048	-3.135121	-0.193556
C	-2.826279	-4.082505	0.091689
C	-4.027703	-3.375951	0.082811
H	-5.012323	-3.763026	0.285715
C	-3.750266	-2.01574	-0.190829
C	-1.166723	-5.896153	0.327631
C	-0.862966	-7.243186	0.575477
H	0.169375	-7.570923	0.560301
C	-1.868555	-8.158549	0.837196
H	-1.619311	-9.195307	1.026422
C	-3.206049	-7.745817	0.85603
H	-3.991085	-8.463449	1.05981
C	-3.527648	-6.421043	0.612961
H	-4.565195	-6.110195	0.626913
C	-2.524474	-5.477089	0.346551
C	-4.668088	-0.948281	-0.244096
C	-6.11067	-1.288328	-0.283077
C	-6.660567	-2.008339	-1.347195
C	-8.010538	-2.328023	-1.396531
C	-8.847947	-1.926239	-0.360315
C	-8.330941	-1.208103	0.713478
C	-6.977879	-0.897141	0.740845
C	-4.318405	0.412109	-0.263781
C	-5.133711	1.529787	-0.56451
H	-6.172892	1.469298	-0.844485
C	-4.345295	2.681603	-0.480097
C	-3.036734	2.23779	-0.138621

C	-1.995838	3.154867	-0.029007
C	-2.251058	4.549182	-0.166933
C	-1.01817	5.183189	-0.032148
H	-0.82182	6.240911	-0.083393
C	-0.008986	4.194777	0.135471
C	-4.636001	4.092946	-0.651664
C	-5.920741	4.556059	-0.971331
H	-6.724307	3.844113	-1.116253
C	-6.174903	5.911927	-1.097702
H	-7.172784	6.253862	-1.342875
C	-5.145069	6.839475	-0.905198
H	-5.346035	7.899266	-1.00162
C	-3.867497	6.404643	-0.591951
H	-3.075539	7.12921	-0.445555
C	-3.586581	5.037121	-0.460973
C	1.377131	4.430863	0.159639
C	1.807034	5.859955	0.125956
C	2.210585	6.511937	1.289323
C	2.62457	7.838305	1.277196
C	2.640428	8.538032	0.074651
C	2.245125	7.910461	-1.102866
C	1.835577	6.58374	-1.064326
C	2.41196	3.482181	0.124912
C	3.792288	3.724925	-0.135626
H	4.21841	4.706374	-0.262392
C	4.442951	2.496222	-0.263809
C	3.444498	1.506106	-0.054626
C	3.743943	0.156672	-0.182253
C	5.057979	-0.263476	-0.525216
C	5.001774	-1.656331	-0.616693
H	5.800452	-2.326845	-0.88849

C	3.671226	-2.073358	-0.337218
C	5.805303	2.104305	-0.587008
C	6.823803	3.048757	-0.775476
H	6.598438	4.103263	-0.670376
C	8.11388	2.651064	-1.089022
H	8.888905	3.394392	-1.229106
C	8.415563	1.291971	-1.222406
H	9.424306	0.982159	-1.465835
C	7.425257	0.339686	-1.040504
H	7.667462	-0.711464	-1.141641
C	6.113208	0.717253	-0.72253
C	3.148638	-3.376469	-0.337769
C	4.12362	-4.494318	-0.400598
C	5.038303	-4.719412	0.631101
C	5.954921	-5.761492	0.582294
C	5.97143	-6.608607	-0.52147
C	5.073365	-6.409293	-1.565639
C	4.164351	-5.362356	-1.494311
F	1.313673	-1.837771	2.462596
F	1.679348	0.221958	3.077235
F	0.088441	-0.92902	4.013296
O	0.330967	0.089943	0.640066
O	-1.450285	-0.110169	1.996886
C	-0.25331	-0.189219	1.740741
C	0.702924	-0.690212	2.863584
Sum of electronic and thermal Free Energies= -4768.364601			

$H_3 2^{\bullet 2+}$ $\bullet(CF_3CO_2^-)_2$

F	4.256004	-4.840577	1.927218
F	6.390061	-6.47477	1.803133
F	8.122066	-6.31037	-0.290109
F	7.695126	-4.49499	-2.275954
F	5.552523	-2.871253	-2.18705
F	1.747364	5.877233	-2.618483
F	2.241801	8.516754	-2.825281
F	1.601123	10.194051	-0.777191
F	0.462185	9.205489	1.489931
F	-0.016353	6.566225	1.725247
F	-5.986703	-1.547704	-1.756169
F	-8.4822	-2.573536	-1.814174
F	-9.514687	-3.779916	0.398316
F	-8.035694	-3.945751	2.678846
F	-5.543943	-2.910676	2.754742
N	-1.905152	-2.315915	0.567746
H	-1.445883	-1.524709	1.032805
N	1.209699	-2.828717	0.28309
H	1.120991	-1.948742	0.797867
N	2.932261	-0.531117	-0.244238
H	2.114925	-0.6739	-0.871819
N	1.720523	2.437672	-0.407403
H	0.938754	1.829234	-0.661124
N	-1.12693	2.963692	0.089722
H	-0.55988	2.283088	0.613793
N	-3.003807	0.480494	0.425395
H	-2.115918	0.142654	0.049554
C	-3.264986	-2.576295	0.447032
C	-3.406287	-3.933046	0.049362
H	-4.348173	-4.41046	-0.167813
C	-2.133562	-4.479187	-0.097932
C	-1.213201	-3.446249	0.243272
C	0.158159	-3.669027	0.11087
C	0.639626	-4.926035	-0.367566

C	2.025875	-4.806419	-0.455535
H	2.724917	-5.544299	-0.815973
C	2.37777	-3.489067	-0.05986
C	-1.67487	-5.778081	-0.557815
C	-2.569264	-6.810846	-0.879542
H	-3.634355	-6.641311	-0.774708
C	-2.104256	-8.038532	-1.325447
H	-2.807332	-8.826542	-1.568739
C	-0.727884	-8.260445	-1.461399
H	-0.365277	-9.220229	-1.810452
C	0.173098	-7.253599	-1.150048
H	1.237135	-7.429156	-1.257611
C	-0.27608	-6.003538	-0.695718
C	3.652441	-2.895097	-0.062497
C	4.827278	-3.794937	-0.125074
C	5.073393	-4.744689	0.868697
C	6.172588	-5.588131	0.824067
C	7.062063	-5.500786	-0.237451
C	6.84598	-4.570077	-1.24384
C	5.742066	-3.734078	-1.178589
C	3.894421	-1.510124	-0.010813
C	5.106896	-0.862124	0.326942
H	6.016745	-1.367351	0.609844
C	4.884685	0.51253	0.28791
C	3.516635	0.692937	-0.087108
C	2.988997	1.986254	-0.177534
C	3.836537	3.108823	0.075535
C	3.035866	4.241007	-0.027285
H	3.331986	5.264405	0.135587
C	1.708278	3.82524	-0.298627
C	5.759089	1.636634	0.557551

C	7.103737	1.466711	0.924334
H	7.505269	0.464732	1.019324
C	7.915598	2.564403	1.16246
H	8.951856	2.419031	1.444556
C	7.398191	3.860708	1.037422
H	8.034341	4.718225	1.223208
C	6.072904	4.049611	0.677617
H	5.677824	5.054314	0.582661
C	5.23355	2.950528	0.432975
C	0.581174	4.67267	-0.324708
C	0.843572	6.130205	-0.441527
C	1.431026	6.67163	-1.585189
C	1.687465	8.028371	-1.708929
C	1.356878	8.885898	-0.669282
C	0.772106	8.379921	0.482712
C	0.52406	7.019384	0.584961
C	-0.754532	4.264468	-0.203387
C	-1.943427	5.033782	-0.352477
H	-1.970693	6.078899	-0.616474
C	-3.03109	4.190089	-0.123961
C	-2.480165	2.899656	0.129567
C	-3.310727	1.803962	0.33264
C	-4.719944	1.979606	0.416819
C	-5.257045	0.704679	0.582861
H	-6.297159	0.447077	0.702989
C	-4.189688	-0.237292	0.547419
C	-4.468829	4.40022	-0.07044
C	-5.046069	5.661305	-0.285974
H	-4.406614	6.503196	-0.525083
C	-6.41807	5.840441	-0.191522
H	-6.84715	6.82142	-0.359072

C	-7.247561	4.757306	0.123521
H	-8.319362	4.898862	0.198838
C	-6.699899	3.501151	0.337362
H	-7.345205	2.6636	0.576072
C	-5.314927	3.295717	0.24581
C	-4.305986	-1.634746	0.536351
C	-5.689952	-2.188727	0.504304
C	-6.473334	-2.120974	-0.645878
C	-7.754767	-2.649352	-0.692757
C	-8.281751	-3.26791	0.432117
C	-7.525369	-3.35329	1.592279
C	-6.246246	-2.817316	1.615631
F	1.318004	0.085663	4.450476
F	2.491701	-0.932493	2.927626
F	2.575084	1.228667	3.092368
O	0.110421	1.446135	2.019648
O	0.072106	-0.801637	1.785827
C	0.513956	0.291543	2.202742
C	1.745576	0.16894	3.162179
F	-2.50573	-0.638436	-2.419633
F	-1.110438	-0.598262	-4.084761
F	-1.251588	-2.36387	-2.819707
O	-0.48251	0.39628	-1.028273
O	0.999505	-0.858754	-2.16922
C	-0.135134	-0.428383	-1.896488
C	-1.267537	-1.011982	-2.802588
Sum of electronic and thermal Free Energies= -5294.927370			
**H**$_3$**2**$^{2+}$•$CF_3CO_2^-$			
C	4.303016	-0.391251	-0.303705
C	5.313668	0.565132	-0.587549

H	6.328505	0.324658	-0.862517
C	4.75041	1.838196	-0.490535
C	3.377464	1.651471	-0.155361
C	2.534114	2.739462	-0.03852
C	3.038656	4.067459	-0.17446
C	1.950556	4.922845	-0.037012
H	1.960139	5.999026	-0.098905
C	0.771118	4.13919	0.139697
C	-0.547965	4.625424	0.169587
C	-1.738993	3.881892	0.122103
C	-3.058485	4.364431	-0.135982
H	-3.312409	5.40471	-0.262537
C	-3.911273	3.268461	-0.267299
C	-3.108704	2.109358	-0.066146
C	-3.639592	0.843384	-0.196051
C	-5.013347	0.66251	-0.524352
C	-5.217069	-0.713524	-0.623788
H	-6.133112	-1.218824	-0.88491
C	-3.979883	-1.37237	-0.365112
C	-3.713355	-2.751498	-0.363465
C	-2.443812	-3.351321	-0.280557
C	-2.153925	-4.711995	0.001969
H	-2.900885	-5.466126	0.190318
C	-0.771616	-4.870168	0.054354
C	-0.195787	-3.590888	-0.215086
C	1.180239	-3.432366	-0.206027
C	2.029514	-4.54609	0.082831
C	3.338801	-4.072655	0.066689
H	4.234787	-4.635219	0.275351
C	3.311397	-2.680907	-0.214093
C	4.401919	-1.792287	-0.281125

C	5.298932	3.173002	-0.657818
C	6.649461	3.390436	-0.975952
H	7.304586	2.539212	-1.12044
C	7.152371	4.67624	-1.101274
H	8.197797	4.825952	-1.344809
C	6.311898	5.780587	-0.909928
H	6.706581	6.785475	-1.005292
C	4.974317	5.590245	-0.599291
H	4.326484	6.446841	-0.453319
C	4.442388	4.297233	-0.469531
C	-0.704458	6.109403	0.133504
C	-0.758436	6.811323	-1.067794
C	-0.915124	8.188888	-1.110788
C	-1.023002	8.901128	0.074981
C	-0.973513	8.23239	1.289516
C	-0.816855	6.853913	1.304291
C	-5.323181	3.128099	-0.586768
C	-6.157288	4.241267	-0.776108
H	-5.740688	5.237203	-0.678142
C	-7.5006	4.081312	-1.081278
H	-8.129907	4.952396	-1.222126
C	-8.043806	2.796537	-1.205833
H	-9.094031	2.671936	-1.442847
C	-7.239256	1.681661	-1.024675
H	-7.662857	0.688435	-1.120143
C	-5.876825	1.817702	-0.715095
C	-4.880653	-3.669579	-0.422558
C	-5.775011	-3.788102	0.641065
C	-6.858775	-4.652617	0.602147
C	-7.072701	-5.433809	-0.524344
C	-6.20356	-5.340649	-1.601654

C	-5.127997	-4.467664	-1.539047
C	0.061874	-6.021267	0.348339
C	-0.482498	-7.286849	0.622626
H	-1.55848	-7.41557	0.615678
C	0.339385	-8.367399	0.901435
H	-0.096243	-9.337489	1.110711
C	1.731455	-8.2065	0.913888
H	2.373118	-9.052023	1.133016
C	2.289671	-6.966365	0.64743
H	3.366466	-6.844508	0.659212
C	1.475577	-5.857922	0.361094
C	5.762156	-2.382695	-0.326792
C	6.685804	-2.180396	0.700043
C	7.964508	-2.716023	0.661847
C	8.353392	-3.486829	-0.423969
C	7.459944	-3.713301	-1.46102
C	6.188572	-3.162948	-1.402622
N	3.116785	0.301783	-0.060117
H	2.431707	-0.034236	0.63324
N	1.179012	2.809023	0.156836
H	0.608339	1.974988	0.224009
N	-1.821503	2.50021	0.170534
H	-1.143495	1.836264	0.530903
N	-3.03637	-0.38334	-0.121824
H	-2.113945	-0.496601	0.288999
N	-1.222401	-2.693373	-0.398945
H	-1.093656	-1.784525	-0.818241
N	1.977265	-2.329045	-0.377537
H	1.653888	-1.435703	-0.717993
F	-0.660114	6.152292	-2.233805
F	-0.966005	8.835696	-2.283488

F	-1.174333	10.229079	0.046845
F	-1.078179	8.921309	2.434068
F	-0.772091	6.237873	2.496243
F	-5.589875	-3.067017	1.757838
F	-7.691233	-4.750086	1.647846
F	-8.112021	-6.273196	-0.571139
F	-6.414134	-6.086578	-2.69486
F	-4.319246	-4.394396	-2.609041
F	6.344129	-1.461629	1.780914
F	8.821797	-2.50503	1.670034
F	9.583644	-4.007536	-0.471864
F	7.836112	-4.449851	-2.515762
F	5.365088	-3.385431	-2.440325
C	-0.80442	-0.56873	2.955366
C	0.219781	-0.206489	1.835102
F	-0.226896	-1.002244	4.084921
F	-1.655078	-1.537837	2.538247
F	-1.561521	0.509719	3.273229
O	-0.316032	0.218072	0.771356
O	1.414011	-0.362082	2.088867
Sum of electronic and thermal Free Energies= - 4768.544794			
[**1**$^{\bullet+}$•$CF_3CO_2^-$]			
S	-0.417489	-0.282504	-1.166426
O	-1.072548	-0.663586	0.163428
O	1.008229	-0.767521	-1.193536
O	-1.245112	-0.952534	-2.241452
O	-0.434349	1.2139	-1.325216
F	-0.483092	-1.483633	2.913049
F	-0.086763	0.096286	4.35059
F	-1.628237	0.361452	2.842349

O	1.678378	-0.233571	1.897541
O	0.490296	1.689383	1.833057
C	0.699585	0.501218	2.131868
C	-0.394616	-0.145678	3.041124
C	-3.233607	-3.53391	-0.815094
C	-4.526919	-3.954906	-0.37658
C	-5.334407	-2.817889	-0.328433
C	-4.513884	-1.708672	-0.738263
C	-4.842598	-5.295007	0.222879
H	-4.240485	-6.068826	-0.250869
H	-5.886282	-5.554676	0.024702
C	-4.574866	-5.329432	1.738057
H	-4.78232	-6.3223	2.146328
H	-3.53081	-5.084708	1.946549
H	-5.198532	-4.606118	2.267661
C	-6.68683	-2.772639	0.327806
H	-7.449605	-2.397602	-0.357857
H	-6.983266	-3.797605	0.559056
C	-6.71886	-1.942785	1.623112
H	-7.701121	-2.014427	2.09745
H	-5.969502	-2.302687	2.331704
H	-6.511312	-0.889577	1.429837
C	-4.69653	-0.319201	-0.615324
C	-5.858899	0.495231	-0.784164
C	-5.533962	1.778481	-0.371512
C	-4.147996	1.762404	0.022864
C	-7.093514	0.085057	-1.531089
H	-7.154617	-1.000183	-1.563128
H	-7.988707	0.437876	-1.00961
C	-7.092162	0.601903	-2.981426
H	-7.982196	0.254814	-3.512847

H	-7.077213	1.692402	-3.020178
H	-6.210751	0.237288	-3.513724
C	-6.430339	2.97283	-0.517953
H	-6.627558	3.419098	0.457913
H	-7.400048	2.623476	-0.878142
C	-5.895714	4.048367	-1.478086
H	-6.621839	4.858716	-1.583134
H	-4.958645	4.474022	-1.117805
H	-5.710503	3.625641	-2.468338
C	-3.32205	2.833942	0.431067
C	-3.61926	4.031845	1.150949
C	-2.489978	4.851721	1.059648
C	-1.510218	4.132792	0.328005
C	-4.792514	4.313798	2.04827
H	-4.49475	5.113987	2.730451
H	-5.633247	4.724471	1.478957
C	-5.254839	3.114587	2.887911
H	-6.04576	3.417374	3.579107
H	-5.640307	2.302935	2.269695
H	-4.422757	2.714598	3.471554
C	-3.206764	7.295574	1.232678
H	-2.995718	8.233378	1.753474
H	-3.074789	7.46703	0.16288
H	-4.257496	7.048735	1.398364
C	-2.284664	6.174535	1.736137
H	-2.437678	6.047864	2.814662
H	-1.24024	6.469729	1.619747
C	-0.183633	4.508576	-0.077562
C	0.278337	5.649025	-0.775767
C	1.646997	5.450969	-1.029416
C	1.994258	4.202017	-0.449287

C	-0.566881	6.777547	-1.289414
H	-1.618997	6.523608	-1.146082
H	-0.428113	6.864464	-2.37327
C	-0.264836	8.137384	-0.640148
H	-0.915961	8.915716	-1.0474
H	-0.415089	8.102088	0.440287
H	0.770398	8.437915	-0.816301
C	2.470661	6.275574	-1.97845
H	3.337145	6.715424	-1.480646
H	1.866682	7.120397	-2.314715
C	2.932129	5.477387	-3.208924
H	3.496885	6.113573	-3.896222
H	3.567544	4.637091	-2.922497
H	2.072035	5.070376	-3.74569
C	3.21637	3.482756	-0.34958
C	4.552102	3.9228	-0.122972
C	5.355494	2.788913	0.008748
C	4.513615	1.645625	-0.149156
C	4.992029	5.33663	0.123685
H	5.537447	5.372231	1.073159
H	4.111625	5.963795	0.267735
C	5.887447	5.917067	-0.983441
H	6.156292	6.952794	-0.75934
H	6.812028	5.343941	-1.082114
H	5.386924	5.89599	-1.952156
C	6.782561	2.811749	0.474271
H	7.445411	2.417135	-0.299543
H	7.080433	3.852845	0.606628
C	7.010009	2.065548	1.798805
H	8.055306	2.146375	2.108217
H	6.386368	2.491456	2.588315

H	6.758948	1.007231	1.71745
C	4.746602	0.262933	-0.077844
C	5.887075	-0.560516	-0.390379
C	5.511462	-1.877559	-0.159751
C	4.145784	-1.857363	0.281676
C	7.166034	-0.140951	-1.05347
H	7.756537	0.485643	-0.381119
H	7.77026	-1.034212	-1.222753
C	6.95884	0.582528	-2.393807
H	7.921353	0.831243	-2.848288
H	6.405349	-0.050613	-3.091123
H	6.391735	1.506251	-2.268844
C	6.25841	-3.10508	-0.593945
H	7.323078	-2.994603	-0.369088
H	5.910687	-3.971871	-0.03419
C	6.066734	-3.389518	-2.094714
H	6.596206	-4.300388	-2.386435
H	5.006817	-3.520349	-2.32465
H	6.4409	-2.565993	-2.706802
C	3.274256	-2.940734	0.542751
C	3.509755	-4.162523	1.225845
C	2.359953	-4.945317	1.067725
C	1.439263	-4.178313	0.298606
C	4.622576	-4.37996	2.211504
H	4.946287	-5.423285	2.206136
H	5.490625	-3.779711	1.934987
C	4.195556	-3.973618	3.634982
H	5.018941	-4.116183	4.34026
H	3.89648	-2.923494	3.656598
H	3.345276	-4.56484	3.98074
C	2.082132	-6.265607	1.728709

H	2.168195	-6.140297	2.813629
H	1.041026	-6.540022	1.549062
C	3.01246	-7.410708	1.294217
H	2.701483	-8.353246	1.752902
H	3.008687	-7.540321	0.210802
H	4.044209	-7.222079	1.595494
C	0.152486	-4.528623	-0.223419
C	-0.286601	-5.720275	-0.855008
C	-1.627332	-5.521763	-1.22885
C	-1.987706	-4.225806	-0.812115
C	0.551776	-6.902601	-1.248686
H	0.940923	-7.411906	-0.366101
H	-0.093821	-7.631092	-1.74426
C	1.717252	-6.541191	-2.183908
H	2.268324	-7.437999	-2.480707
H	1.349876	-6.049685	-3.087778
H	2.415694	-5.858669	-1.696397
C	-2.442837	-6.401382	-2.132549
H	-2.39292	-7.442678	-1.7974
H	-3.49171	-6.1062	-2.08187
C	-1.991481	-6.30512	-3.601014
H	-2.623244	-6.927062	-4.241207
H	-2.056327	-5.272307	-3.950786
H	-0.957367	-6.63236	-3.724358
N	-3.264022	-2.198345	-1.021952
H	-2.483133	-1.664513	-1.505361
N	-3.669881	0.495915	-0.192216
H	-2.734115	0.138542	0.09497
N	-2.010128	2.912882	0.013387
H	-1.477123	2.196964	-0.546907
N	0.854618	3.647705	0.075074

H	0.810355	2.802501	0.691062
N	3.224026	2.116358	-0.328488
H	2.430405	1.533841	-0.588088
N	3.714196	-0.571905	0.277433

H	2.844858	-0.285221	0.807432
N	1.992123	-2.956851	0.05428

Table S7.

Optimized geometries and energies (*E*) for species involved in the electron transport chain comprised of **1**, I_2, and **2**.

References

(1) Sheldrick, G.M. SHELXT-integrated space-group and crystal-structure determination. *Acta Cryst. A.* **2015**, *71*, 3–8. https://doi.org/10.1107/S2053273314026370.

(2) Rw(F2) = {w(|Fo|2 - |Fc|2)2/w(|Fo|)4}1/2 where w is the weight given each reflection. R(F) = (|Fo| - |Fc|)/ |Fo|} for reflections with Fo > 4((Fo)). S = [w(|Fo|2 - |Fc|2)2/(n - p)]1/2, where n is the number of reflections and p is the number of refined parameters.

(3) Wilson, A.J.C. International tables for X-ray crystallography. Vol. C, Tables 4.2.6.8 and 6.1.1.4,; Kluwer Academic Press, **1992**.

(4) Sheldrick, G.M.. SHELXTL/PC (Version 5.03).; Siemens Analytical X-ray Instruments, Inc., Wisconsin, **1994**.

(5) Becke, A.D. Density-functional thermochemistry. III. The role of exact exchange. *J. Chem. Phys.* **1993**, *98*, 5648–5652. https://doi.org/10.1063/1.464913.

(6) Grimme, S., Antony, J., Ehrlich S. and Krieg, H. A consistent and accurate ab initio parametrization of density functional dispersion correction (DFT-D) for the 94 elements H-Pu. *J. Chem. Phys.* **2010**, *132*, 154104. https://doi.org/10.1063/1.3382344.

(7) Hay, P.J., and Wadt, W.R. Ab initio effective core potentials for molecular calculations. Potentials for K to Au including the outermost core orbitals. *J. Chem. Phys.* **1985**, *82*, 299–310. https://doi.org/10.1063/1.448975.

(8) Ehlers, A.W., Böhme, M., Dapprich, S., Gobbi, A., Höllwarth, A., Jonas, V., Köhler, K.F., Stegmann, R., Veldkamp, A., and Frenking, G. A set of f-polarization functions for pseudo-potential basis sets of the transition metals Sc Cu, Y Ag and La Au. *Chem. Phys. Lett.* **1993**, *208*, 111–114. https://doi.org/10.1016/0009-2614(93)80086-5.

(9) Weigend, F., and Ahlrichs, R. Balanced basis sets of split valence, triple zeta valence and quadruple zeta valence quality for H to Rn: Design and assessment of accuracy. Phys. *Chem. Chem. Phys.* **2005**, *7*, 3297–3305. https://doi.org/10.1039/B508541A.

(10) Pritchard, B.P., Altarawy, D., Didier, B., Gibson, T.D., and Windus, T.L. New basis set exchange: an open, up-to-date resource for the molecular sciences community. *J. Chem. Inf. Model.* **2019**, *59*, 4814–4820. https://doi.org/10.1021/acs.jcim.9b00725.

(11) Marenich, A.V., Cramer, C.J., and Truhlar, D.G. Universal solvation model based on solute electron density and on a continuum model of the solvent defined by the bulk dielectric constant and atomic surface tensions. *J. Phys. Chem. B* **2009**, *113*, 6378–6393. https://doi.org/10.1021/jp810292n.

(12) Job, P. Study of new selective reagent acetophenone 2', 4'- dihydroxy semicarbazone for extractive spectrophotometric determination of vanadium. *Ann. Chim.* **1928**, *9*, 113–203. https://doi.org/10.12691/wjac-2-1-3.

(13) Connors, K.A. Binding Constants.; John Wiley and Sons, New York, **1987**.

(14) Gans, P., Sabatini, A., and Vacca. A. Investigation of equilibria in solution. Determination of equilibrium constants with the HYPERQUAD suite of programs. Talanta. **1996**, *43*, 1739–1753. https://doi.org/10.1016/0039-9140(96)01958-3.
